# Whole genome sequencing reveals the genetic diversity and structure of *Leptosphaeria maculans* populations from the Western Cape province of South Africa

**DOI:** 10.1186/s12864-025-11413-3

**Published:** 2025-04-03

**Authors:** Huibrecht Maria Schreuder, Beatrix Coetzee, Gerhardus Johannes van Coller, Diane Mostert

**Affiliations:** 1 Department of Plant Pathology, Faculty of AgriSciences, Stellenbosch University, Stellenbosch, 7602 South Africa; 2 School for Data Science and Computational Thinking, Stellenbosch University, Stellenbosch, 7602 South Africa; 3 Directorate: Plant Sciences, Western Cape Dept. of Agriculture, Elsenburg, 7607 South Africa

**Keywords:** Whole genome sequencing, Single nucleotide polymorphisms, Population genomics, Population structure, *Leptosphaeria maculans*, Blackleg, Canola

## Abstract

**Background:**

*Leptosphaeria maculans* is the causal agent of blackleg, a globally important disease of canola. Investigating the genetic diversity and structure of *L. maculans* populations can provide insight into its evolutionary potential and genetic variability, which is important to develop effective blackleg management strategies. In this study, whole genome sequence data was generated for 230 *L. maculans* isolates collected between 2020 and 2022 across the canola production regions of the Western Cape of South Africa. A total of 27 419 informative single nucleotide polymorphisms was used to investigate the genetic diversity and structure of the pathogen population.

**Results:**

Mating type distribution did not deviate statistically from a 1:1 ratio at any location, indicating no restriction on sexual reproduction. Genetic statistics calculated showed high genotypic diversity and evenness (Lambda and E.5 ≥ 0.98) and low linkage disequilibrium ( ≤ 2.71E-04) which is also associated with sexual reproduction. Discriminative analysis of principal components and sparse nonnegative matrix factorisation revealed genetic differentiation between the Swartland and Southern Cape canola production regions in the Western Cape. Analysis of molecular variance also indicated regions as the most important factor for population differentiation but suggested shallow population structure with only 3,71% of the total variation occurring between regions. To assess the phylogenomic position of South African isolates in the global context, data for 171 international isolates was included, and the clustering analyses repeated. Results showed a high similarity between Australian and Swartland isolates, while isolates from the Southern Cape formed a unique genetic cluster.

**Conclusion:**

The results from this study provide the basis for blackleg research in South Africa and enhances understanding of the pathogen, which will assist in developing improved blackleg management strategies.

**Supplementary Information:**

The online version contains supplementary material available at 10.1186/s12864-025-11413-3.

## Background

*Leptosphaeria maculans* Ces. and De Not. and *L. biglobosa* Shoemaker and H. Brun are two closely related fungal species causing blackleg, a globally important disease on canola (*Brassica napus* L.) [1, 2]. They co-occur everywhere brassicas are grown, except for China, where only *L. biglobosa* has been reported [2, 3]. These pathogens can infect its host at any stage of its life cycle and cause symptoms such as root rot, stem and crown cankers, and lesions on stems, branches, inflorescences and pods [4, 5]. *Leptosphaeria maculans* is considered the more virulent of the two species and is associated with damaging crown cankers that causes severe blackleg epidemics [4]. Therefore, research on blackleg of canola has primarily focused on *L. maculans*.

*Leptosphaeria maculans* has both sexual and asexual reproduction cycles [6]. It is a heterothallic species in which a cross between its two mating types, MAT1-1 and MAT1-2, results in the formation of pseudothecia on stubble [7]. It also produces large quantities of air-borne ascospores that can be dispersed over large distances [4]. This gives *L. maculans* a high evolutionary potential and allows it to adapt in response to environmental pressures [8], which can present challenges for the management of blackleg disease. Therefore, genetic analysis to monitor changes in *L. maculans* populations is vital to ensure effective pathogen management. It can allow for the detection of the molecular foundations of adaptation and enable predictions of host resistance breakdown or development of fungicide resistance [9, 10]. Population genetic studies can also provide insights into the evolutionary potential of pathogens in different regions, elucidate pathogen dispersal patterns and allow for inferences to be made on the environmental and geographic factors influencing genetic differentiation between regions [9, 10].

In previous genetic studies on *L. maculans* populations, DNA markers such as random amplified polymorphic DNA (RAPD) [11], amplified fragment length polymorphism (AFLP) [12–14], micro- and minisatellite markers [15–17] and single nucleotide polymorphisms (SNPs) [18] were employed to explore the genetic diversity and population structure of the pathogen. These studies mostly revealed unstructured populations with high levels of genetic variation at small spatial scales. The sequencing of the genome of a *L. maculans* isolate (JN3) was an important advancement to study the pathogen, and provided a reference to map molecular markers and whole genome resequencing data [19]. Genome wide single nucleotide polymorphisms are useful molecular markers in fungal population genetic studies due to its abundance, ease of use in high-throughput assessments and the wealth of information it holds with regards to mutation, recombination, and dispersal.

Canola in South Africa is produced almost exclusively in the Western Cape Province, which consists of the Swartland and Southern Cape production regions. Given the importance of blackleg in South African canola production, it is important that the genetic diversity and structure of the local *L. maculans* populations are investigated, as little knowledge exist about the local population. In this study the genomes of 230 *L. maculans* isolates, collected from locations across the Western Cape province of South Africa between 2020 and 2022, were sequenced. Genome data of an additional 34 South African *L. maculans* isolates was obtained from GenBank and included in the study. Genome wide SNPs was identified and used to investigate the diversity and structure of the local *L. maculans* populations. Whole genome sequence data generated by Van de Wouw et al. [20] of an additional 171 isolates of international origin was also exploited to determine the phylogenomic position of the South African *L. maculans* populations in a global context. The results offer a valuable resource for blackleg management, and the genomic data generated will serve as a basis for comparison in future studies.

## Materials and methods

### Fungal isolates

**Fig. 1:**
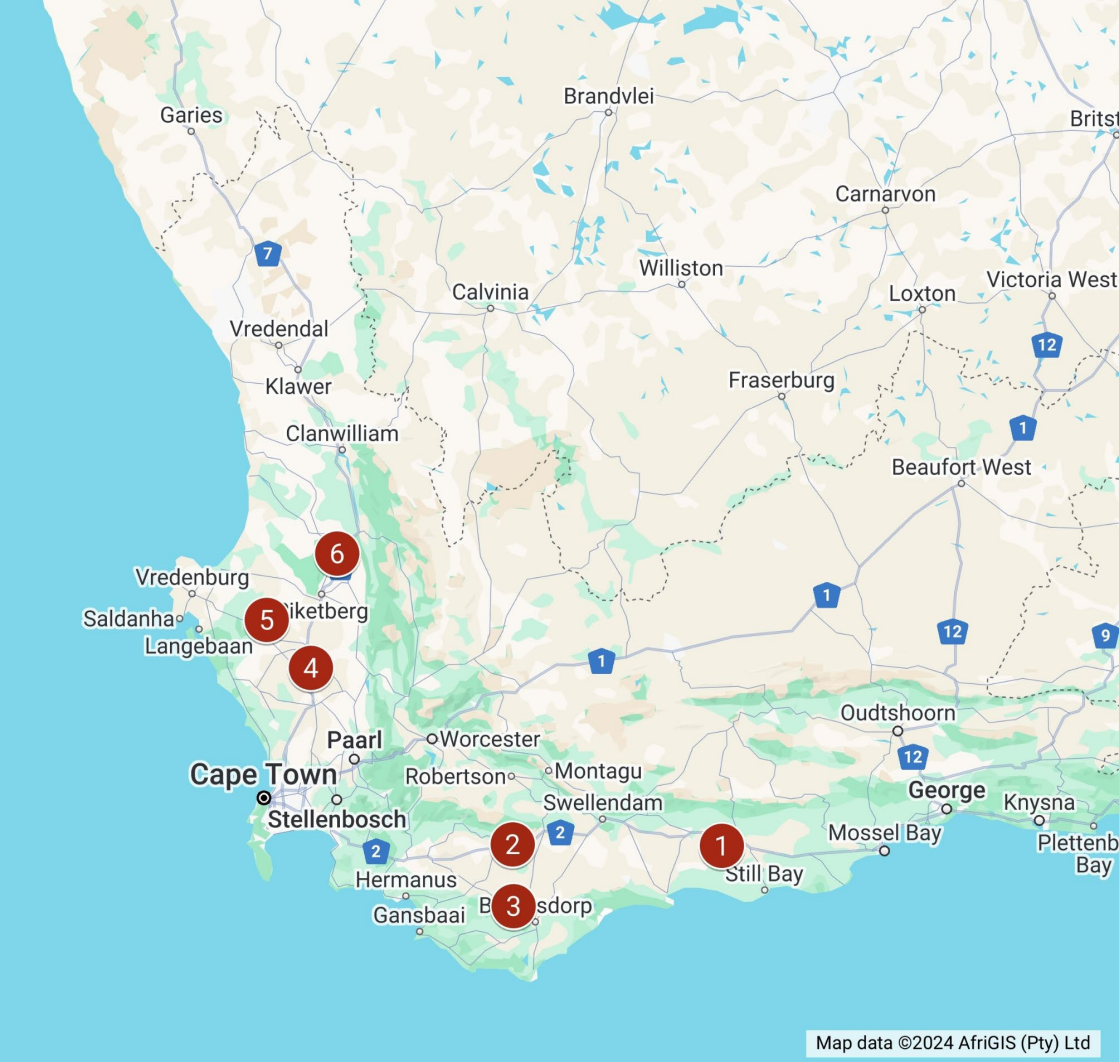
Map indicating the locations in the Western Cape of South Africa where canola stubble samples were collected for the isolation of *Leptosphaeria* species used in this study. Locations in the Southern Cape production area: (1) Uitkyk farm near Riversdale (2) Tygerhoek research farm near Riviersonderend (3) Syngenta® research farm near Napier. Locations in the Swartland production region: (4) Langgewens research farm near Moorreesburg (5) Waterboerskraal farm near Hopefield (6) Baviaanskloof farm near Eendekuil. Map created with Google My Maps

Canola stubble was collected from cultivar trials conducted between 2020 and 2022 after harvest, at six locations. Three locations were selected in the Southern Cape production region: Uitkyk farm near Riversdale (-34.16026, 21.15369), Tygerhoek research farm near Riviersonderend (-34.16861, 19.91516) and Syngenta® research farm near Napier (-34.456944, 19.913056). In the Swartland production region, Langgewens research farm near Moorreesburg (-33.27463, 18.70643), Waterboerskraal farm near Hopefield (-33.03349, 18.43882) and Baviaanskloof farm near Eendekuil (-32.706111, 18.861944) were selected (Fig. 1). These sites are used for cultivar trials on an annual basis and the same cultivars were present across locations each year. Stubble from each cultivar at each location were kept separate as samples. Stubble samples were thoroughly washed with water to remove soil before rinsing with clean water. The tap roots and tops of the stubble were trimmed with pruning shears to approximately 40 cm. Twenty stubble pieces per sample (location-cultivar) were placed on a tray lined with wet paper towels before sealing the tray in a clear plastic bag to form a moisture chamber. Moisture chambers was incubated at room temperature until pycnidia with cirrhi (mucus bound conidial exudate) was visible under a stereo microscope after approximately five days.

To obtain single spore isolates, one cirrhus per stubble piece was picked up using a sterile scalpel blade and transferred into a 13 mL McCartney bottle containing 5 mL of sterile deionised water. The bottle was shaken to separate and distribute conidia, and the contents poured over a water agar dish. Spores were given 5 min to settle on the surface of the water agar after which the water was carefully poured off and petri dishes incubated in a slanted position at room temperature. After two days, single germinating spores were cut from the media under a stereo microscope and transferred to Petri dishes containing quarter strength potato dextrose agar (¼ PDA). Petri dishes were sealed with parafilm and incubated at 22 °C with a 12/12 h photoperiod for approximately ten days.

### Identification of isolates

The morphology of ten-day old cultures grown on ¼ PDA was used to identify isolates as *Leptosphaeria* species. Colony characteristics included growth rate (diameter of approximately 6 cm), thin and felty mycelia ranging between white, grey-brown and dark brown, black globose or sub-globose pycnidia and in some cases sectoring and small white tufts of thick white mycelia. Isolates identified as *Leptosphaeria* were assigned numbers and preserved in sterile water at 4 °C and in 30% glycerol at -80 °C.

For the isolates identified as *Leptosphaeria* species, mycelia were scraped from the surface of the media and lyophilized in a VirTis BenchTop Pro (SP Scientific, Warminster, USA) freeze drier. DNA extractions were done using the method described by González-Mendoza et al. [21]. The pellets obtained from the extractions were resuspended in 60 μL TE buffer (10 mL L^− 1^ 1 M Tris at pH 8.0- and 2-mL L^− 1^ 0.5 M EDTA). Three microlitres of RNase (10 mg mL^− 1^) were added to the tubes before incubating in a 65 °C water bath for 10 min. The quantity and quality of DNA was checked with a NanoDrop Nd-1000 (Thermo Scientific, Waltham, USA) spectrophotometer. DNA was stored at 4 °C until use.

To identify isolates as *L. maculans*, a region within the internal transcribed spacer region was amplified using the species specific primers LmacR (5′ -GCAAAATGTGCTGCGCTCCAGG- 3′) and LmacF (5′ -CTTGCCCACCAATTGGATCCCCTA-3′) [22]. Amplifications were performed in a total volume of 25 μL containing 50 ng of DNA, 80 μM of each deoxyribonucleotide triphosphate, 0.2 μM of each primer, 1 unit of BIOTAQ™ DNA polymerase in 1X NH_4_ reaction buffer (Meridian Bioscience, Cincinnati, USA), 2.8 mM MgCl_2_ and 0.8 μg/μL bovine serum albumin. PCR reactions were performed in a 96-well Veriti® Thermal Cycler (Applied Biosystems, Foster City, USA) with a initial denaturation step of 2 min at 95 °C, followed by 37 cycles of 15 s at 95 °C, 30 s at 70 °C, and 1 min at 72 °C and a final elongation step of 10 min at 72 °C. Amplicons were electrophoresed on 1% agarose gel with 0.01% SYBR™ safe (Invitrogen, Waltham, USA) DNA gel stain and then visualised using a Gel Doc™ XR + Gel Documentation System (Invitrogen, Waltham, USA). A fragment size of 331 bp served as a positive identification of *L. maculans*.

### Genome sequencing and SNP detection

A sub-population of 230 *L. maculans* isolates were selected from the larger collection of isolates to represent all sampling locations, sampling years and resistance gene (*R*-gene) groups [23] of cultivars from which isolations were done (Table S1). Mycelia of these isolates were scraped from the surface of ten-day old cultures grown on ¼ PDA and inoculated into 75 mL of 20% clarified V8 broth (360 ml V8 original and 5 g CaCO_3_ mixed thoroughly, centrifuged at 1 700 RCF for 20 min, supernatant diluted 1:4 with sterile deionised water, sterilised by autoclaving) and shake-incubated in a LOM-150 orbital shaker-incubator (MRC laboratory-instruments, Harlow, UK) at 22 °C and 100 revolutions per minute (RPM) for 10 days. Mycelia was harvested with sterilised cheese cloth, rinsed with sterile water and lyophilised. Approximately 50 mg of freeze-dried mycelia of each isolate was added to a 2 mL Eppendorf tube and grinded to a fine powder using a pellet pestle. DNA was extracted using the E.Z.N.A Plant and Fungal DNA Mini Kit (Omega Bio-Tek, Norcross, USA) as per manufacturer’s instructions. DNA quality and quantity were checked using a spectrophotometer and a Qubit™ 4 Fluorometer (Invitrogen, Waltham, USA). DNA fragments were electrophoresed on 1% agarose and visualised using a Gel Doc™ XR + to assess the integrity. DNA was stored at -20 °C until use.

DNA was sent to NovogeneAIT genomics (Singapore) where the genomes of the *L. maculans* isolates were sequenced in four lanes of an Illumina NovaSeq6000 machine (Illumina, San Diego, California, USA) resulting in 150 bp paired end reads. Sequencing libraries were prepared according to the manufacturer’s instructions. High throughput sequencing (HTS) data for 205 additional isolates (including 34 South African isolates) generated during a recently published global population study [20] were downloaded from NCBI’s Sequence Read Archive (SRA) (BioProject PRJNA902499). Details on these isolates are provided in Table S2. Quality trimming was performed with TrimGalore version 0.4.1 [24], removing adapters, clipping the first five nucleotides from the 5’ end of both reads and removing nucleotides at read ends below Phred 20. Only reads that were 100 nucleotides in length after trimming and remained paired, were retained.

High throughput sequencing reads from each of the isolates were mapped against the *L. maculans* reference genome JN3 (GenBank: GCA_900538235.1) [25] using BWA version 0.7.13 [26]. The resulting sam files were converted to bam, sorted and duplicates marked using Genome Analysis Toolkit version 4.2.5.0 (GATK, Broad Institute, https://software.broadinstitute.org/gatk/). The variants were then called with GATK’s HaplotypeCaller mode to create a vcf file for each isolate. The vcf files were combined with GATK’s GenomicsDBImport and variants recalibrated with GenotypeGVCFs. The variants were filtered using the VariantFiltration mode and SelectVariants, selecting only SNPs with the same parameters as previously described by Van de Wouw et al. [20]. The remaining variants were pruned for SNPs in linkage disequilibrium in Plink v2.00a5 [27]. Scrips for sequence analysis can be obtained from GitHub: https://github.com/beatrixcoetzee/population_genomics.

### Mating type designation

The whole genome sequence from each isolate was assembled using Spades v.3.13.0 [28] with default parameters. Mating types were determined by means of in silico PCR available in iPCRess 2.2.0 (part of the Exonorate package) [29] by using the *L. maculans* mating type multiplex primer set, MAT-Locus (TGGCGAATTAAGGGATTGCTG), MAT1-1 (CTCGATGCAATGTACTTGG) and MAT1-2 (AGCCGGAGGTGAAGTTGAAGCCG) [7]. Deviation from a 1:1 ratio of isochores was tested using Pearson’s Chi square (Chi^2^) tests and two sided binom.test functions in R. *P*-values were adjusted with Benjamini-Hochberg [30] correction method for multiple testing. The scripts for mating type sequence extractions can be obtained from GitHub: https://github.com/beatrixcoetzee/population_genomics.

### Population analysis

The single vcf file containing the SNP information of the 264 South African *L. maculans* isolates was read into R (version 4.0.5, R Core Team, 2021) using the package vcfR [31]. Analysis of molecular variance (AMOVA) was performed on the data using the R package poppr [32, 33] to test differentiation by grouping (locality, region, collection year, cultivar type). Pairwise distance (PhiST) values were calculated for populations using the haplotypes package [34]. To assess genetic diversity and the level of sexual reproduction, descriptive population statistics were calculated using poppr. This included Nei’s unbiased gene diversity (Hexp) that measures expected heterozygosity in a population, Simpson’s index (lambda) that assesses genetic diversity, evenness (E.5) that indicates how evenly individuals are distributed among genotypes and standardised index of association $\:\:({\stackrel{-}{r}}_{d}$) that measures linkage disequilibrium in a population [35–39]. The significance of $\:{\stackrel{-}{r}}_{d}$ was tested based on 999 permutations. To evaluate genotypic diversity in terms of genotypic richness and evenness of distribution, the number of multilocus genotypes (MLGs) and expected multilocus genotypes (eMLG) in each population was assessed with poppr. Closely related genotypes were merged into multilocus lineages (MLLs), using the nearest neighbour algorithm [33] and a distance threshold of 0.01. Isolates that collapsed into the same MLL were treated as clones, and lambda, E.5 and $\:{\stackrel{-}{r}}_{d}$ were recalculated using a clone corrected dataset.

K-means clustering, as implemented in the discriminant analysis of principal components (DAPC) method [40] in the adegenet package [41, 42] was used to detect the optimal number of subpopulations that has the lowest Bayesian information criterion (BIC). The optimal number of principal components (PCs) was assessed with cross-validation [40] and selected based on lowest root mean squared error (RMSE) with 30 replicates. The DAPC was conducted with the first 140 principal components and all (two) discriminant functions. The clone corrected dataset was used to perform sparse nonnegative matrix factorisation (SNMF). The SNMF function contained in the LEA package [43] was used to estimate the possible number of clusters, running 10 replicates over a range of K values (1 to 20), using the best estimate of K, and extracting individual admixture coefficients from the replicate with the lowest cross-entropy.

Individual vcf files for 171 *L. maculans* isolates from other countries [20], were combined with those from South African isolates. This collection contained isolates from Argentina, Australia, Canada, Czechia, France, Germany, Iran, New Zealand and the USA. It also included two isolates generated through in vitro crossing between European isolates. After recalibration, filtering, selection and pruning for linkage disequilibrium, the combined vcf file were further analysed as described above to access the phylogenomic relationship of South African isolates to the global population. The DAPC was constructed using the first 100 PCs and four discriminant functions.

## Results

### Establishing the South African *L. maculans* culture collection

A total of 2 744 of the isolates obtained from stubble sampled between 2020 and 2022 were morphologically identified as *Leptosphaeria* species. Of these, 1 996 were identified as *L. maculans* with species specific PCRs (Table 1). Most isolates collected at locations in the Swartland as well as Riversdale were identified as *L. maculans*, ranging from 63.3% at Hopefield 2021 to 99.04% at Eendekuil in 2022. Similarly, more than 85% of isolates collected in 2020 at Napier and Tygerhoek respectively was *L. maculans*, while less than 50% of isolates collected at these locations in 2021 and 2022 were identified as *L. maculans* (Table 1). Because the remaining isolates were already identified as *Leptosphaeria* species, they are likely *L. biglobosa*. Further identification will be required to confirm this species identity which was outside the scope of this study.

**Table 1:** Number of *Leptosphaeria maculans* isolates identified in a *Leptosphaeria* collection collected at six locations across the canola production regions in the Western cape between 2020 and 2022

Location		2020			2021			2022			Grand Total	Total LM
		Total	LM^a^		Total	LM		Total	LM			
Eendekuil					103	93		104	103		207	196
Hopefield		34	33		109	69		171	149		314	251
Langgewens		248	234		177	145		222	167		647	546
Napier		134	114		120	16		149	44		403	174
Riversdale		199	197		133	99		207	157		539	453
Tygerhoek		193	168		199	97		242	111		634	376
Grand Total		808	746		841	519		1095	731		2744	1996

^a^Number of isolates identified as *L. maculans*

### Genome sequencing and SNP detection

Whole genome sequencing produced between 6 515 041 and 19 340 869 read pairs (average 8 928 467) per isolate amounting to 1.95 to 5.8 Gbases (average 2.68 Gbases) of data. After quality trimming, more than 96% of the data was retained. Given the genome length of 45 Mb, this equates to a theoretical genome coverage of between 41.03 and 121.93 times (average 56.24 times) per isolate. Mapping and filtering of variants extracted 27 419 high quality and informative SNPs, present in all isolates, relative to the *L. maculans* JN3 [25] reference genome. Breadth of coverage across the genome ranged from 91.3 to 95.72% (average 94.59%). Sequencing statistics for each isolate can be found in Table S3.

The HTS dataset of 205 international isolates downloaded from NCBI had between 3 648 280 and 19 858 120 read pairs per isolates. After quality filtering and trimming, 1.06 Gbases to 5.75 Gbases were retained per isolate, with an average theoretical genome coverage of 55.14 times. Data for individual isolates is provided in Table S4. Combined variant calling and filtering for the complete isolate set of 435 isolates, identified 54 864 informative SNPs.

### Distribution of mating types

Across the collection 51.15% of isolates were of MAT1-1 mating type and 48.85% of MAT1-2. Idiomorphs did not deviate significantly from a 1:1 ratio at any of the sampling locations (Table 2). Mating type information for individual isolates are provided in Table S5.

**Table 2:** Leptosphaeria maculans mating type distributions at the six locations where isolates were collected

Location	MAT1-1	MAT1-2	Ratio (MAT1-1/MAT1-2)	Binomial *P*-value^a^	Chi	Chi *P*-value^a^
Eendekuil	15	14	1,071428571	1	0,0345	0,8527
Hopefield	29	21	1,380952381	0.38664	1,28	0,30948
Langgewens	20	30	0,666666667	0.3645	2	0,28395
Napier	23	14	1,642857143	0.3645	2,1892	0,28395
Riversdale	28	19	1,473684211	0.3645	1,7234	0,28395
Tygerhoek	19	30	0,633333333	0.3645	2,4694	0,28395

^a^ Benjamini-Hochberg corrected

### Population analysis

#### South African isolates

The population structure of South African *L. maculans* isolates was investigated using descriptive populations genetic statistics, AMOVA, DAPC and SNMF. Each isolate represented a unique MLG. At a genetic distance threshold of 0.01, the 264 MLGs collapsed into 252 MLLs. Overall, the population had high genotypic diversity with 243 isolates (92%) representing unique MLLs. Genotypic richness was the highest at Riversdale, Napier and Eendekuil, where each isolate represented a unique MLL. Tygerhoek and Langgewens had 50 MLGs each which collapsed into 49 and 48 MLLs, respectively. Hopefield had the lowest genotypic richness with 50 MLGs collapsing into 41 MLLs. There were no shared MLLs between locations. Simpson and evenness indices followed the same patterns, with the highest values found at Riversdale, Napier and Eendekuil (lambda and E.5 values of 1) followed by Tygerhoek (0.9992 and 0.998), Langgewens (0.9984 and 0.996) and Hopefield (0.9894 and 0.976). Nei’s unbiased gene diversity index showed that isolates from the Southern Cape are more heterozygous (0.095) than isolates from the Swartland (0.086). There was a decrease in gene diversity from east to west (Hexp values gradually decreasing from 0.094 at Riversdale and Napier to 0.0837 at Eendekuil). The standardised index of association was close to zero (< 0.001) at all locations, suggesting sexual reproduction occurs. Linkage disequilibrium, which indicates clonal reproduction, however, was significant at all locations except Napier (Table 3).

AMOVA results revealed low, but significant (*P* = 0,01) variation between locations, regions and years, with region as the most important contributor to between population variation. Variation between locations, regions and years was 2.5%, 3.71% and 0.16% respectively, with the remainder of variation occurring within respective groups. Pairwise PhiST between locations, regions and years did not show significant spatial or temporal distinction (*P* > 0.05).

**Table 3:** Summary of population statistics for the genetic diversity in *Leptosphaeria maculans* populations in the Western cape Province

Location	*N*	Hexp	MLG	eMLG	MLL	Lambda^a, b^	E.5^b^	${\bar r_d}$ ^b^	*p*.rD
Eendekuil	30	0.0837	30	30	30	1	1	2.71E-04	0,01
Hopefield	50	0.0857	50	30	41	0.9894	0.976	0.00075428	0,01
Langgewens	50	0.0847	50	30	48	0.9984	0.996	8.34E-05	0,01
Napier	37	0.0944	37	30	37	1	1	7.61E-06	0,3
Riversdale	47	0.0943	47	30	47	1	1	3.05E-05	0,01
Tygerhoek	50	0.0939	50	30	49	0.9992	0.998	9.38E-05	0,01

*N* = number of isolates (sample size); Hexp = Nei’s unbiased gene diversity index; MLG = number of observed multilocus genotypes; eMLG = number of expected MLG at a sample size of 30; MLL = number of observed multilocus lineages using a bitwise cutoff distance of 0.01; lambda = Simpson’s index; E.5 = Evenness; $\:{\stackrel{-}{r}}_{d}$ = Standarised index of association; p.rD = *P*-value for $\:\:{\stackrel{-}{r}}_{d}$, *P* < 0.05 indicates significant linkage disequilibrium

^a^Corrected for sample size; ^b^ Calculated using clone corrected data

**Fig. 2:**
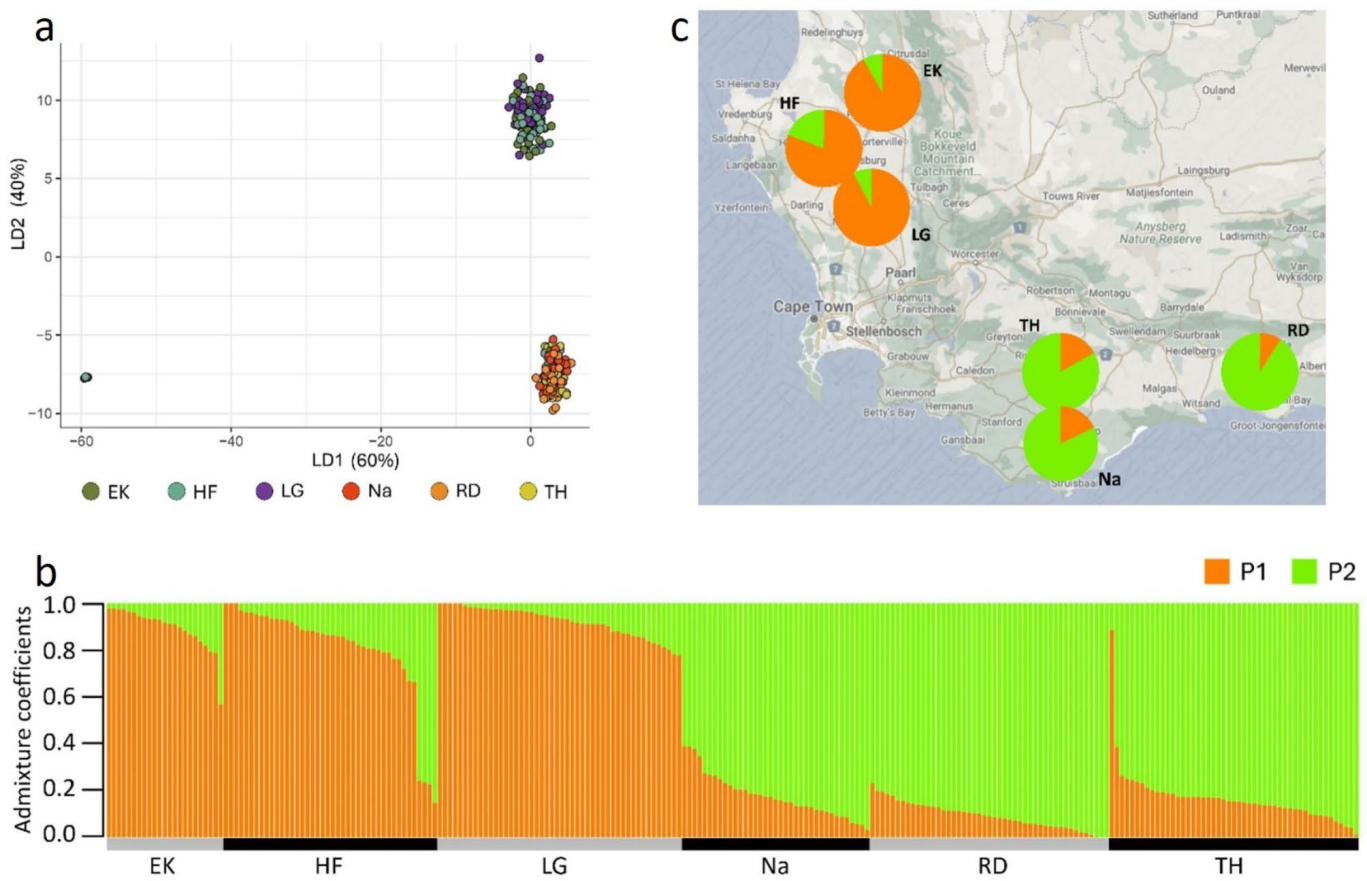
Visualisations of population analyses conducted using 27 419 informative single nucleotide polymorphisms (SNPs) for 264 *Leptosphaeria maculans* isolates collected at six locations in the Western Cape province. EK = Eendekuil, HF = Hopefield, LG = Langgewens, Na = Napier, RD = Riversdale and TH = Tygerhoek. (**a**) Scatterplot generated from the discriminant analysis of principal components (DAPC) using the first 140 principal components (PCs) and all (two) discriminant functions (or linear discriminants, LD). Each point represents an individual isolate, and colours denote their location of origin. (**b**) Admixture plot for K = 2 showing admixture coefficients for subpopulations P1 and P2 as determined by sparse nonnegative matrix factorisation (SNMF) (R package LEA). Each column represents an individual isolate with the length of each colour segment denoting the proportions of the subpopulations assigned to the isolate with no prior on location. Origin of isolates are shown under the horizontal axis. (**c**) Map showing the geographical distributions of the two subpopulations (P1 and P2), based on average admixture proportions. Colour codes are shared by **b** and **c**

The DAPC clustered the South African isolates into two subpopulations (lowest BIC value = 1812.237). The two subpopulations corresponded to the two production regions, with isolates from the Swartland (Langgewens, Hopefield and Eendekuil) clustering separately from isolates from locations in the Southern Cape (Riversdale, Tygerhoek and Napier) (Fig. 2a). This was supported by the AMOVA results that identified the two production regions as the most important factor for population differentiation. There was one isolate from Tygerhoek clustering with the Swartland isolates and four isolates from Hopefield clustering with Southern Cape isolates. Information on DAPC clustering for individual isolates is provided in Supplementary Table S5.

In the admixture analysis, the lowest cross entropy was also found for two subpopulations (K = 2), hereafter referred to as P1 and P2. Population one was the dominant subpopulation in the Swartland (average admixture = 88.05%), while isolates in the Southern Cape mainly contained admixture from P2 (average 85.55%) (Fig. 2b, c). The distinct admixture compositions in isolates collected in the Southern Cape versus isolates collected in the Swartland supports the AMOVA and DAPC results. Admixture coefficients for individual isolates are provided in Table S5.

#### International isolate collection

**Fig. 3:**
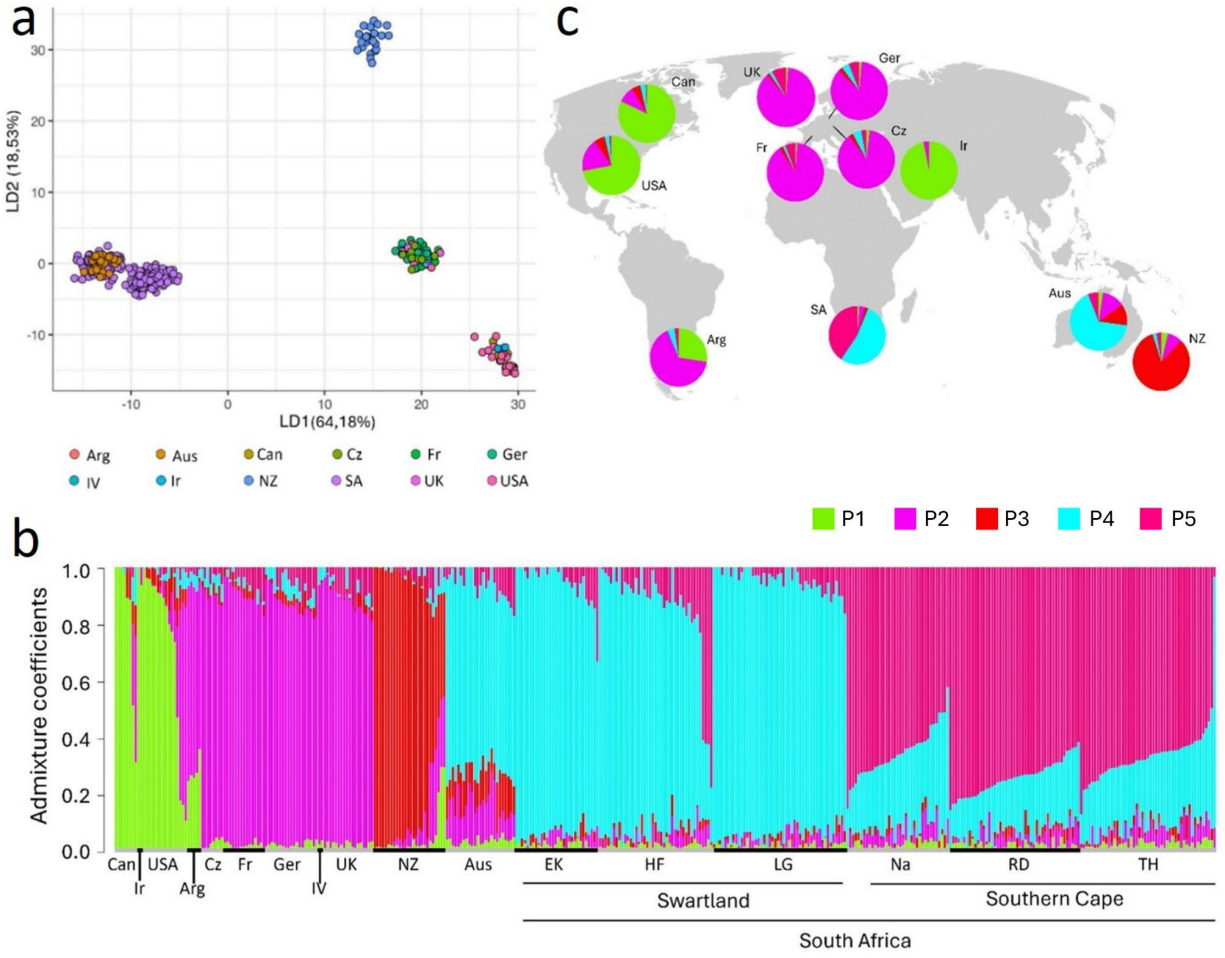
Visualisations of population analyses conducted using 54 864 informative single nucleotide polymorphisms (SNPs) in 435 *Leptosphaeria maculans* isolates collected across 12 countries. Arg = Argentina; Aus = Australia; Can = Canada; Cz = Czechia; Fr = France; Ger = Germany; IV = In vitro; Ir = Iran; Nz = New Zealand; SA = South Africa; UK = United Kingdom; USA = United States of America (**a**) Scatterplot generated from the discriminant analysis of principal components (DAPC) using the first 100 principal components (PC’s) and four discriminant functions (LD). Each point represents an individual isolate, and colours denote their country-of-origin (**b**) Admixture plot for K = 5 showing admixture coefficients for different subpopulations (P1-P5) as determined by sparse nonnegative matrix factorisation (SNMF) on clone corrected data (R package LEA). Each column represents an individual isolate with the length of each colour segment denoting the proportions of a subpopulation assigned to the isolate with no prior on location. (**c**) Map showing the geographical distributions of the five subpopulations (P1-P5) based on mean admixture proportions. Colour codes for P1-P5 is shared by **b** and **c**

To determine the phylogenomic position of South African *L. maculans* isolates within the global population, the newly sequenced isolates were combined with the international isolate collection [20], and DAPC and SNMF repeated on this dataset. The DAPC supported five subpopulations (lowest BIC value = 3173.816) (Fig. 3a). Australian isolates formed a cluster with South African isolates from the Swartland, while isolates from the Southern Cape formed a unique cluster. Isolates from New Zealand were also separated into a unique cluster. European isolates (France, Germany, Czech Republic and in vitro) formed a cluster with UK and Argentine isolates. Two isolates from Canada, three from New Zealand and five from the USA were also included in this cluster. North American (USA and Canadian) isolates clustered with Iranian isolates (Fig. 3a). Information on DAPC clustering for individual isolates are provided in Table S6.

In the SNMF for the international collection of *L. maculans*, the lowest cross entropy (0.1044772) was found for five subpopulations (K = 5), hereafter referred to as P1-P5 (Fig. 3b). Population 1 was predominant in isolates from Canada, Iran and USA (average admixture coefficients 81.97%, 97.65% and 72.13% respectively). Isolates from Argentina (65.99%), Czech (87.86%), France (89.54%), Germany (85.93%) and the UK (87.35%) mainly contained admixture from P2. New Zealand isolates primarily contained admixture from P3 (82.9%). Population 4 was predominant in Australian isolates and isolates from the Swartland (66.86% and 84.54% respectively), while isolates from the Southern Cape mainly contained admixture from P5 (69,49%) (Fig. 3b, c). Both the DAPC and SNMF supports the Southern Cape isolates as a unique cluster, while isolates from the Swartland and Australia clustered together.

## Discussion

This study reports on the population structure of 264 *L. maculans* isolates collected across the canola production regions of South Africa isolated between 2020 and 2022. Analysis of whole genome SNP data revealed the isolates from the two canola production regions, Swartland and Southern Cape, as separate genetic clusters. The AMOVA indicated only 3.71% variation between these regions, with the remainder occurring within the regions. The shallow structure was also supported by the lack of significant pairwise distance (PhiST). This suggests recent geneflow between these regions whereafter differentiation occurred. The two production regions are more than 80 km apart and are separated by the Hottentots Holland and Boland mountains that creates a natural barrier of over 40 km in diameter. This barrier could have restricted gene flow between the regions. Genetic differences between regions could be the result of genetic drift and/or different selection pressures. The higher intensity of canola cultivation in the Southern Cape would sustain a larger pathogen population size which provides higher pathogen genetic diversity [44]. Furthermore, the wet season is also longer in the Southern Cape than in the Swartland, with areas in the Southern Cape receiving between 50% and 70% of its rainfall from late autumn to mid-spring and areas in the Swartland receiving 80% of its rainfall in the winter [45, 46]. Infected canola stubble (crop residues) from the previous season is the primary source of inoculum for the development of blackleg [2, 47]. When sufficient moisture is available, the development of pseudothecia, as a result of sexual reproduction on infected stubble, and the subsequent release of ascospores can occur as long as infected stubble remains on the field [47]. The longer duration of the rain season in the Southern Cape will, therefore, allow for sexual reproduction to occur over a longer period. This can increase genetic diversity and drive differentiation from the Swartland population. In support of this, Nei’s gene diversity index showed a decrease in gene diversity from east to west, a direction which also encompasses reduction in canola cultivation intensity and a decrease in the length of the rain season. The longer rain season in the Southern Cape allows producers to plant crops over summer months. In the Swartland, rotation systems consist of wheat, medics, cover crops, oats and lupine, while in the Southern Cape, pastures are grown for 5 years followed by a 5-year period of cereal crop such as wheat, barley and canola. The difference in cropping systems could have shaped the agroecosystems within regions and contributed to genetic differentiation between regions.

The SNMF analysis on the international isolate collection assigned Australian and Swartland isolates to the same cluster, while isolates from the Southern Cape formed a separate unique cluster not represented prominently in other regions or countries. This subpopulation might have its origin from a region or country not included in the present study. However, the high evolutionary potential of *L. maculans* also presents the possibility that this subpopulation originated in the Southern Cape through genetic differentiation. A likely scenario is the introduction of *L. maculans* from Australia, and the subsequent differentiation between the two regions. *Leptosphaeria maculans* has been reported on cabbage in South Africa before the start of canola production [48]. Brassicas and their associated organisms might also have a natural history in South Africa that precedes the introduction of cultivated brassicas. South Africa has a high diversity of brassica species, many of which only occur locally [49, 50]. Brassicas are also exclusive hosts to diamondback moth (*Plutella xylostella* L.) [51]. There is an exceptionally high level of diversity and endemicity in diamondback moth parasitoids in South Africa, which suggests an association between brassicas and diamond back moth that exceeds the < 300 year period within which cultivated brassicas arrived in the country [50]. A similar theory regarding brassicas and *L. maculans* in South Africa might, therefore, explain the unique genetic cluster in the Southern Cape.

The population statistics calculated showed high genetic diversity (Simpsons index, MLG and MLL), an equal distribution of individuals among genotypes (evenness index) and low linkage disequilibrium. Mating type distributions did not deviate statistically from a 1:1 ratio at any location. These results are as expected from a sexually reproducing population. The consistent observation of pseudothecia in stubble on the field after the onset of autumn rain at the start of the new canola growth season further supports the existence of a sexually reproducing population. However, the low but significant $\:\:{\stackrel{-}{r}}_{d}\:$ values found at all locations except Napier, and the 21 isolates that did not represent unique MLLs, indicates that clonal reproduction plays a small role in the epidemiology of *L. maculans* at some locations.

Similar results were found between this study and that of Van de Wouw et al. [20]., where a principle component analysis (PCA) separated isolates into three clusters: Europe, UK and Argentina; USA, Canada and Iran; and Australia, New Zealand and South Africa. However, with the inclusion of 230 additional South African isolates in the current study, isolates from New Zealand were separated from South African and Australian isolates. The difference in results between the studies was initially solely attributed to sampling bias, with the number of South African isolates included being substantially higher than the number of isolates from other countries. However, when a DAPC with only the isolates included in Van de Wouw et al. [20]. and a PCA including the entire collection was conducted, the New Zealand isolates formed their own cluster in the DAPC and merged with the European cluster in the PCA (Fig. S1). The differing results between analyses suggests that the New Zealand cluster is not well defined and need further investigation.

An even distribution of mating types and shallow population structure with high genetic variation within small spatial scales was also found in European [52, 53], Canadian [11, 54] and Australian [13, 18] populations. However, idiomorph ratios diverging significantly from a 1:1 ratio, clonality, high linkage disequilibrium and high genetic differentiation between locations or regions have been reported in some Canadian populations [11, 17, 55]. This is likely the result of shorter cultivation periods in Canada and environmental conditions that are less favourable to sexual reproduction [17].

The genetic variation observed between South African production regions could influence virulence and it is, therefore, important that the effect of the SNP variation on isolate virulence should be determined. Major gene resistance in canola is governed by gene-for-gene interactions (Flor, 1955; Balesdent et al., 2005), whereby the presence of a resistance gene in the host allows it to recognise a pathogen with the corresponding avirulence gene and prevent infection (Delourme et al., 2006). This can lead to mutations within avirulence genes that can result in the rapid breakdown of major gene resistance in canola cultivars (Rouxel et al., 2003; Sprague et al., 2006; Rouxel and Balesdent, 2010). The cloning 12 of *L. maculans* avirulence genes (*AvrLm1-L3*, *Avrlm2*, *AvrLm3*, *AvrLm4-7*, *Avrlm5-9*, *Avrlm6*, *Avrlm10A*, *AvrLm10B*, *AvrLm14*, *AvrLmS-Lep2*, *AvrLep1*, *AvrLmSTEE98*) has enabled the determination of race structure in *L. maculans* populations using molecular markers, as was recently done with a global *L. maculans* population (Van de Wouw et al., 2024). Cornelsen et al. (2021) correlated the frequency of avirulence alleles with results of field trials assessing disease severity over multiple sites and years to determine the efficacy of deployed resistance genes. A similar approach can be considered to regularly phenotype virulence of the South African *L. maculans* isolates to monitor the efficacy of host resistance of canola cultivars planted.

## Conclusion

This is the first study to investigate the structure of South African *L. maculans* populations. It revealed population differentiation between the two canola production regions, the Swartland and Southern Cape. Results suggest that the Western Cape *L. maculans* populations are sexually reproducing populations with high genetic diversity. It is, therefore, essential that genetic changes in the South African *L. maculans* population are monitored to allow for the prediction and subsequent prevention or future breakdowns of host resistance and fungicide efficacy. The *Leptosphaeria* collection established and whole genome data generated in this study, therefore, forms an important basis for comparison in future studies.

## Electronic supplementary material

Below is the link to the electronic supplementary material.

Supplementary Material 1

Supplementary Material 2

Supplementary Material 3

Supplementary Material 4

Supplementary Material 5

Supplementary Material 6

Supplementary Material 7

## Data Availability

The data that support the findings of this study are available online as supplementary information and in GenBank BioProject at https://www.ncbi.nlm.nih.gov/bioproject/ with accession number PRJNA1191806.

## Abbreviations

RAPD: Random amplified polymorphic DNA
AFLP: Amplified fragment length polymorphism
SNP: Single nucleotide polymorphisms
NGS: Next generation sequencing
RCF: Relative centrifugal force
HTS: High throughput sequencing
SRA: Sequence Read Archive
AMOVA: Analysis of molecular variance
PhiST: Pairwise distance
Hexp: Nei’s unbiased gene diversity
E.5: Evenness index
r ®d: Standardised index of association
Lambda: Simpson’s index
MLG: Multilocus genotypes
eMLG: Expected multilocus genotypes
MLL: Multilocus lineages
DAPC: Discriminant analysis of principal components
BIC: Bayesian information criterion
PC: Principal component
RMSE: Root mean squared error
SNMF: Sparse nonnegative matrix factorisation
