# Supplementary material for: Whole genome sequencing reveals the genetic diversity and structure of Leptosphaeria maculans populations from the Western Cape province of South Africa
Source: BMC Genomics. 2025 Apr 3;26:334. doi: 10.1186/s12864-025-11413-3 (PMC11966903; doi:10.1186/s12864-025-11413-3)
Supplement: Supplementary file 1 — Supplementary Material 1 [file 12864_2025_11413_MOESM1_ESM.docx]

**Table S1** Information on the origin of 230 *Leptosphaeria maculans* isolates for which whole genome sequence data was generated

| **Isolate number** | **Year** | **Region** | **Location** | **Host cultivar** |
| --- | --- | --- | --- | --- |
| STE-U 9674 | 2020 | Southern Cape | Riversdale | Hyola® 50 |
| STE-U 9675 | 2020 | Southern Cape | Riversdale | Hyola® 580CT |
| STE-U 9679 | 2020 | Southern Cape | Riversdale | Pioneer® 45Y93 |
| STE-U 9680 | 2020 | Southern Cape | Riversdale | CB™ Tango C |
| STE-U 9681 | 2020 | Southern Cape | Riversdale | Alpha TT |
| STE-U 9682 | 2020 | Southern Cape | Riversdale | Alpha TT |
| STE-U 9683 | 2020 | Southern Cape | Riversdale | Nuseed® Quartz |
| STE-U 9684 | 2020 | Southern Cape | Riversdale | Nuseed® Quartz |
| STE-U 9685 | 2020 | Southern Cape | Riversdale | Pioneer® 45Y91 CL |
| STE-U 9687 | 2020 | Southern Cape | Riversdale | Hyola® 559TT |
| STE-U 9688 | 2020 | Southern Cape | Riversdale | Pioneer® 44Y90 CL |
| STE-U 9689 | 2020 | Southern Cape | Riversdale | Hyola® 350TT |
| STE-U 9690 | 2020 | Southern Cape | Riversdale | Hyola® 350TT |
| STE-U 9691 | 2020 | Southern Cape | Riversdale | Hyola® 350TT |
| STE-U 9692 | 2020 | Southern Cape | Riversdale | Hyola® 650TT |
| STE-U 9693 | 2020 | Southern Cape | Tygerhoek | Hyola® 50 |
| STE-U 9694 | 2020 | Southern Cape | Tygerhoek | Hyola® 580CT |
| STE-U 9695 | 2020 | Southern Cape | Tygerhoek | Nuseed® Diamond |
| STE-U 9696 | 2020 | Southern Cape | Tygerhoek | Nuseed® Diamond |
| STE-U 9697 | 2020 | Southern Cape | Tygerhoek | Pioneer® 45Y93 |
| STE-U 9698 | 2020 | Southern Cape | Tygerhoek | CB™ Tango C |
| STE-U 9699 | 2020 | Southern Cape | Tygerhoek | CB™ Tango C |
| STE-U 9700 | 2020 | Southern Cape | Tygerhoek | Alpha TT |
| STE-U 9701 | 2020 | Southern Cape | Tygerhoek | Alpha TT |
| STE-U 9702 | 2020 | Southern Cape | Tygerhoek | Nuseed® Quartz |
| STE-U 9703 | 2020 | Southern Cape | Tygerhoek | Pioneer® 45Y91 CL |
| STE-U 9704 | 2020 | Southern Cape | Tygerhoek | Hyola® 555TT |
| STE-U 9705 | 2020 | Southern Cape | Tygerhoek | Hyola® 555TT |
| STE-U 9706 | 2020 | Southern Cape | Tygerhoek | Hyola® 350TT |
| STE-U 9707 | 2020 | Southern Cape | Tygerhoek | Hyola® 350TT |
| STE-U 9708 | 2020 | Southern Cape | Tygerhoek | Hyola® 350TT |
| STE-U 9709 | 2020 | Swartland | Langgewens | Hyola® 50 |
| STE-U 9711 | 2020 | Swartland | Langgewens | Hyola® 580CT |
| STE-U 9712 | 2020 | Swartland | Langgewens | Nuseed® Diamond |
| STE-U 9715 | 2020 | Swartland | Langgewens | CB™ Tango C |
| STE-U 9717 | 2020 | Swartland | Langgewens | Nuseed® Quartz |
| STE-U 9718 | 2020 | Swartland | Langgewens | Nuseed® Quartz |
| STE-U 9719 | 2020 | Swartland | Langgewens | Pioneer® 45Y91 CL |
| STE-U 9720 | 2020 | Swartland | Langgewens | Hyola® 555TT |
| STE-U 9721 | 2020 | Swartland | Langgewens | Hyola® 559TT |
| STE-U 9722 | 2020 | Swartland | Langgewens | Hyola® 559TT |
| STE-U 9723 | 2020 | Swartland | Langgewens | Pioneer® 44Y90 CL |
| STE-U 9724 | 2020 | Swartland | Langgewens | Hyola® 350TT |
| STE-U 9725 | 2020 | Swartland | Langgewens | Hyola® 350TT |
| STE-U 9727 | 2020 | Swartland | Hopefield | Hyola® 50 |
| STE-U 9729 | 2020 | Swartland | Hopefield | Nuseed® Diamond |
| STE-U 9730 | 2020 | Swartland | Hopefield | Nuseed® Diamond |
| STE-U 9731 | 2020 | Swartland | Hopefield | Nuseed® Diamond |
| STE-U 9732 | 2020 | Swartland | Hopefield | Pioneer® 43Y92 CL |
| STE-U 9733 | 2020 | Swartland | Hopefield | Pioneer® 43Y92 CL |
| STE-U 9735 | 2020 | Swartland | Hopefield | CB™ Tango C |
| STE-U 9736 | 2020 | Swartland | Hopefield | CB™ Tango C |
| STE-U 9737 | 2020 | Swartland | Hopefield | CB™ Tango C |
| STE-U 9738 | 2020 | Swartland | Hopefield | Pioneer® 45Y91 CL |
| STE-U 9739 | 2020 | Swartland | Hopefield | Pioneer® 45Y91 CL |
| STE-U 9740 | 2020 | Swartland | Hopefield | Hyola® 559TT |
| STE-U 9741 | 2020 | Swartland | Hopefield | Hyola® 559TT |
| STE-U 9742 | 2020 | Swartland | Hopefield | Hyola® 559TT |
| STE-U 9743 | 2020 | Swartland | Hopefield | Hyola® 559TT |
| STE-U 9744 | 2020 | Swartland | Hopefield | Hyola® 650TT |
| STE-U 9745 | 2020 | Southern Cape | Napier | Hyola® 50 |
| STE-U 9746 | 2020 | Southern Cape | Napier | Hyola® 50 |
| STE-U 9747 | 2020 | Southern Cape | Napier | Hyola® 580CT |
| STE-U 9748 | 2020 | Southern Cape | Napier | Nuseed® Diamond |
| STE-U 9749 | 2020 | Southern Cape | Napier | Nuseed® Diamond |
| STE-U 9750 | 2020 | Southern Cape | Napier | Pioneer® 43Y92 CL |
| STE-U 9751 | 2020 | Southern Cape | Napier | Pioneer® 43Y92 CL |
| STE-U 9752 | 2020 | Southern Cape | Napier | Pioneer® 43Y92 CL |
| STE-U 9753 | 2020 | Southern Cape | Napier | Pioneer® 45Y93 |
| STE-U 9754 | 2020 | Southern Cape | Napier | CB™ Tango C |
| STE-U 9755 | 2020 | Southern Cape | Napier | Alpha TT |
| STE-U 9756 | 2020 | Southern Cape | Napier | Alpha TT |
| STE-U 9757 | 2020 | Southern Cape | Napier | Hyola® 555TT |
| STE-U 9758 | 2020 | Southern Cape | Napier | Pioneer® 44Y90 CL |
| STE-U 9759 | 2020 | Southern Cape | Napier | Pioneer® 44Y90 CL |
| STE-U 9760 | 2020 | Southern Cape | Napier | Hyola® 650TT |
| STE-U 9761 | 2021 | Southern Cape | Riversdale | Pioneer® 43Y92 CL |
| STE-U 9762 | 2021 | Southern Cape | Riversdale | Pioneer® 44Y90 CL |
| STE-U 9763 | 2021 | Southern Cape | Riversdale | Pioneer® 45Y95 CL |
| STE-U 9765 | 2021 | Southern Cape | Riversdale | Nuseed® Diamond |
| STE-U 9766 | 2021 | Southern Cape | Riversdale | Alpha TT |
| STE-U 9767 | 2021 | Southern Cape | Riversdale | Hyola® Blazer TT |
| STE-U 9768 | 2021 | Southern Cape | Riversdale | Hyola® Blazer TT |
| STE-U 9769 | 2021 | Southern Cape | Riversdale | Hyola® Blazer TT |
| STE-U 9770 | 2021 | Southern Cape | Riversdale | Pioneer® 44Y94 CL |
| STE-U 9771 | 2021 | Southern Cape | Riversdale | Pioneer® 44Y94 CL |
| STE-U 9772 | 2021 | Southern Cape | Riversdale | Nuseed® Quartz |
| STE-U 9773 | 2021 | Southern Cape | Riversdale | Hyola® 559TT |
| STE-U 9774 | 2021 | Southern Cape | Riversdale | Hyola® 650TT |
| STE-U 9775 | 2021 | Southern Cape | Riversdale | Hyola® 650TT |
| STE-U 9776 | 2021 | Southern Cape | Tygerhoek | Pioneer® 45Y93 |
| STE-U 9777 | 2021 | Southern Cape | Tygerhoek | Pioneer® 45Y93 |
| STE-U 9778 | 2021 | Southern Cape | Tygerhoek | Pioneer® 44Y90 CL |
| STE-U 9779 | 2021 | Southern Cape | Tygerhoek | Pioneer® 44Y90 CL |
| STE-U 9780 | 2021 | Southern Cape | Tygerhoek | Pioneer® 45Y95 CL |
| STE-U 9781 | 2021 | Southern Cape | Tygerhoek | Nuseed® Diamond |
| STE-U 9782 | 2021 | Southern Cape | Tygerhoek | Nuseed® Diamond |
| STE-U 9783 | 2021 | Southern Cape | Tygerhoek | Alpha TT |
| STE-U 9785 | 2021 | Southern Cape | Tygerhoek | Nuseed® Quartz |
| STE-U 9786 | 2021 | Southern Cape | Tygerhoek | Hyola® 559TT |
| STE-U 9787 | 2021 | Southern Cape | Tygerhoek | Hyola® 650TT |
| STE-U 9789 | 2021 | Swartland | Langgewens | Pioneer® 45Y93 |
| STE-U 9790 | 2021 | Swartland | Langgewens | Pioneer® 44Y90 CL |
| STE-U 9791 | 2021 | Swartland | Langgewens | Pioneer® 45Y95 CL |
| STE-U 9792 | 2021 | Swartland | Langgewens | Pioneer® 45Y95 CL |
| STE-U 9793 | 2021 | Swartland | Langgewens | Nuseed® Diamond |
| STE-U 9794 | 2021 | Swartland | Langgewens | Alpha TT |
| STE-U 9795 | 2021 | Swartland | Langgewens | Alpha TT |
| STE-U 9796 | 2021 | Swartland | Langgewens | Hyola® Blazer TT |
| STE-U 9797 | 2021 | Swartland | Langgewens | Pioneer® 44Y94 CL |
| STE-U 9798 | 2021 | Swartland | Langgewens | Pioneer® 44Y94 CL |
| STE-U 9799 | 2021 | Swartland | Langgewens | Nuseed® Quartz |
| STE-U 9800 | 2021 | Swartland | Langgewens | Nuseed® Quartz |
| STE-U 9801 | 2021 | Swartland | Langgewens | Hyola® 350TT |
| STE-U 9802 | 2021 | Swartland | Langgewens | Hyola® 559TT |
| STE-U 9803 | 2021 | Southern Cape | Napier | Pioneer® 43Y92 CL |
| STE-U 9804 | 2021 | Southern Cape | Napier | Pioneer® 43Y92 CL |
| STE-U 9805 | 2021 | Southern Cape | Napier | Pioneer® 43Y92 CL |
| STE-U 9806 | 2021 | Southern Cape | Napier | Pioneer® 44Y90 CL |
| STE-U 9807 | 2021 | Southern Cape | Napier | Pioneer® 44Y90 CL |
| STE-U 9808 | 2021 | Southern Cape | Napier | Pioneer® 44Y90 CL |
| STE-U 9809 | 2021 | Southern Cape | Napier | Pioneer® 44Y94 CL |
| STE-U 9810 | 2021 | Southern Cape | Napier | Hyola® 559TT |
| STE-U 9811 | 2021 | Swartland | Eendekuil | Pioneer® 43Y92 CL |
| STE-U 9812 | 2021 | Swartland | Eendekuil | Pioneer® 44Y90 CL |
| STE-U 9813 | 2021 | Swartland | Eendekuil | Pioneer® 45Y95 CL |
| STE-U 9814 | 2021 | Swartland | Eendekuil | Nuseed® Diamond |
| STE-U 9815 | 2021 | Swartland | Eendekuil | Alpha TT |
| STE-U 9816 | 2021 | Swartland | Eendekuil | Alpha TT |
| STE-U 9817 | 2021 | Swartland | Eendekuil | Hyola® Blazer TT |
| STE-U 9818 | 2021 | Swartland | Eendekuil | Hyola® Blazer TT |
| STE-U 9819 | 2021 | Swartland | Eendekuil | Pioneer® 44Y94 CL |
| STE-U 9820 | 2021 | Swartland | Eendekuil | Nuseed® Quartz |
| STE-U 9821 | 2021 | Swartland | Eendekuil | Nuseed® Quartz |
| STE-U 9822 | 2021 | Swartland | Eendekuil | Hyola® 350TT |
| STE-U 9823 | 2021 | Swartland | Eendekuil | Hyola® 650TT |
| STE-U 9824 | 2021 | Swartland | Hopefield | Pioneer® 43Y92 CL |
| STE-U 9825 | 2021 | Swartland | Hopefield | Pioneer® 45Y93 |
| STE-U 9827 | 2021 | Swartland | Hopefield | Pioneer® 44Y90 CL |
| STE-U 9828 | 2021 | Swartland | Hopefield | Pioneer® 45Y95 CL |
| STE-U 9829 | 2021 | Swartland | Hopefield | Nuseed® Diamond |
| STE-U 9830 | 2021 | Swartland | Hopefield | Nuseed® Diamond |
| STE-U 9831 | 2021 | Swartland | Hopefield | Alpha TT |
| STE-U 9832 | 2021 | Swartland | Hopefield | Hyola® Blazer TT |
| STE-U 9833 | 2021 | Swartland | Hopefield | Hyola® Blazer TT |
| STE-U 9834 | 2021 | Swartland | Hopefield | Hyola® Blazer TT |
| STE-U 9836 | 2021 | Swartland | Hopefield | Pioneer® 44Y94 CL |
| STE-U 9837 | 2021 | Swartland | Hopefield | Hyola® 559TT |
| STE-U 9838 | 2021 | Swartland | Hopefield | Hyola® 559TT |
| STE-U 9839 | 2021 | Swartland | Hopefield | Hyola® 559TT |
| STE-U 9840 | 2022 | Southern Cape | Riversdale | Pioneer® 43Y92 CL |
| STE-U 9841 | 2022 | Southern Cape | Riversdale | Pioneer® 45Y93 |
| STE-U 9842 | 2022 | Southern Cape | Riversdale | Pioneer® 45Y95 CL |
| STE-U 9843 | 2022 | Southern Cape | Riversdale | Nuseed® Diamond |
| STE-U 9844 | 2022 | Southern Cape | Riversdale | Nuseed® Diamond |
| STE-U 9845 | 2022 | Southern Cape | Riversdale | Alpha TT |
| STE-U 9846 | 2022 | Southern Cape | Riversdale | Pioneer® 44Y94 CL |
| STE-U 9847 | 2022 | Southern Cape | Riversdale | Nuseed® Quartz |
| STE-U 9848 | 2022 | Southern Cape | Riversdale | Hyola® 350TT |
| STE-U 9849 | 2022 | Southern Cape | Riversdale | Hyola® Enforcer CT |
| STE-U 9850 | 2022 | Southern Cape | Riversdale | Hyola® Enforcer CT |
| STE-U 9851 | 2022 | Southern Cape | Riversdale | CC91117 |
| STE-U 9852 | 2022 | Southern Cape | Riversdale | HyTTec® Trophy |
| STE-U 9854 | 2022 | Southern Cape | Riversdale | SF Dynatron™ TT |
| STE-U 9855 | 2022 | Southern Cape | Tygerhoek | Hyola® 90013 |
| STE-U 9856 | 2022 | Southern Cape | Tygerhoek | Pioneer® 43Y92 CL |
| STE-U 9857 | 2022 | Southern Cape | Tygerhoek | Pioneer® 45Y93 |
| STE-U 9858 | 2022 | Southern Cape | Tygerhoek | Pioneer® 45Y95 CL |
| STE-U 9859 | 2022 | Southern Cape | Tygerhoek | Nuseed® Diamond |
| STE-U 9860 | 2022 | Southern Cape | Tygerhoek | Alpha TT |
| STE-U 9861 | 2022 | Southern Cape | Tygerhoek | Alpha TT |
| STE-U 9862 | 2022 | Southern Cape | Tygerhoek | Pioneer® 44Y94 CL |
| STE-U 9863 | 2022 | Southern Cape | Tygerhoek | Nuseed® Quartz |
| STE-U 9864 | 2022 | Southern Cape | Tygerhoek | Nuseed® Quartz |
| STE-U 9865 | 2022 | Southern Cape | Tygerhoek | HyTTec® Trifecta |
| STE-U 9866 | 2022 | Southern Cape | Tygerhoek | HyTTec® Trifecta |
| STE-U 9867 | 2022 | Southern Cape | Tygerhoek | SF Dynatron™ TT |
| STE-U 9868 | 2022 | Swartland | Langgewens | Hyola® 90013 |
| STE-U 9869 | 2022 | Swartland | Langgewens | Pioneer® 43Y92 CL |
| STE-U 9870 | 2022 | Swartland | Langgewens | Pioneer® 43Y92 CL |
| STE-U 9871 | 2022 | Swartland | Langgewens | Pioneer® 45Y93 |
| STE-U 9872 | 2022 | Swartland | Langgewens | Pioneer® 45Y95 CL |
| STE-U 9873 | 2022 | Swartland | Langgewens | Nuseed® Diamond |
| STE-U 9874 | 2022 | Swartland | Langgewens | Nuseed® Diamond |
| STE-U 9875 | 2022 | Swartland | Langgewens | Alpha TT |
| STE-U 9876 | 2022 | Swartland | Langgewens | Hyola® Blazer TT |
| STE-U 9880 | 2022 | Swartland | Langgewens | Hyola® Enforcer CT |
| STE-U 9881 | 2022 | Swartland | Langgewens | CC91117 |
| STE-U 9882 | 2022 | Swartland | Langgewens | HyTTec® Trophy |
| STE-U 9883 | 2022 | Swartland | Langgewens | HyTTec® Trifecta |
| STE-U 9884 | 2022 | Swartland | Langgewens | SF Dynatron™ TT |
| STE-U 9885 | 2022 | Southern Cape | Napier | Pioneer® 45Y93 |
| STE-U 9886 | 2022 | Southern Cape | Napier | Pioneer® 45Y95 CL |
| STE-U 9887 | 2022 | Southern Cape | Napier | Pioneer® 45Y95 CL |
| STE-U 9888 | 2022 | Southern Cape | Napier | Nuseed® Diamond |
| STE-U 9889 | 2022 | Southern Cape | Napier | Alpha TT |
| STE-U 9890 | 2022 | Southern Cape | Napier | Hyola® Blazer TT |
| STE-U 9891 | 2022 | Southern Cape | Napier | Hyola® Blazer TT |
| STE-U 9892 | 2022 | Southern Cape | Napier | Pioneer® 44Y94 CL |
| STE-U 9893 | 2022 | Southern Cape | Napier | Nuseed® Quartz |
| STE-U 9894 | 2022 | Southern Cape | Napier | Nuseed® Quartz |
| STE-U 9896 | 2022 | Southern Cape | Napier | SF Dynatron™ TT |
| STE-U 9897 | 2022 | Swartland | Eendekuil | Hyola® 90013 |
| STE-U 9898 | 2022 | Swartland | Eendekuil | Pioneer® 43Y92 CL |
| STE-U 9899 | 2022 | Swartland | Eendekuil | Pioneer® 45Y93 |
| STE-U 9900 | 2022 | Swartland | Eendekuil | Pioneer® 45Y93 |
| STE-U 9901 | 2022 | Swartland | Eendekuil | Pioneer® 45Y95 CL |
| STE-U 9902 | 2022 | Swartland | Eendekuil | Nuseed® Diamond |
| STE-U 9903 | 2022 | Swartland | Eendekuil | Nuseed® Diamond |
| STE-U 9904 | 2022 | Swartland | Eendekuil | Alpha TT |
| STE-U 9905 | 2022 | Swartland | Eendekuil | Hyola® Blazer TT |
| STE-U 9906 | 2022 | Swartland | Eendekuil | Pioneer® 44Y94 CL |
| STE-U 9907 | 2022 | Swartland | Eendekuil | Nuseed® Quartz |
| STE-U 9908 | 2022 | Swartland | Eendekuil | Hyola® 350TT |
| STE-U 9910 | 2022 | Swartland | Eendekuil | HyTTec® Trophy |
| STE-U 9912 | 2022 | Swartland | Eendekuil | HyTTec® Trifecta |
| STE-U 9914 | 2022 | Swartland | Hopefield | Hyola® 90013 |
| STE-U 9915 | 2022 | Swartland | Hopefield | Pioneer® 43Y92 CL |
| STE-U 9916 | 2022 | Swartland | Hopefield | Pioneer® 45Y93 |
| STE-U 9918 | 2022 | Swartland | Hopefield | Nuseed® Diamond |
| STE-U 9919 | 2022 | Swartland | Hopefield | Alpha TT |
| STE-U 9920 | 2022 | Swartland | Hopefield | Alpha TT |
| STE-U 9921 | 2022 | Swartland | Hopefield | Hyola® Blazer TT |
| STE-U 9922 | 2022 | Swartland | Hopefield | Pioneer® 44Y94 CL |
| STE-U 9923 | 2022 | Swartland | Hopefield | Nuseed® Quartz |
| STE-U 9925 | 2022 | Swartland | Hopefield | Hyola® 350TT |
| STE-U 9927 | 2022 | Swartland | Hopefield | CC91117 |
| STE-U 9928 | 2022 | Swartland | Hopefield | HyTTec® Trophy |
| STE-U 9929 | 2022 | Swartland | Hopefield | SF Dynatron™ TT |
| STE-U 9930 | 2022 | Swartland | Hopefield | SF Dynatron™ TT |
