# Supplementary material for: Whole genome sequencing reveals the genetic diversity and structure of Leptosphaeria maculans populations from the Western Cape province of South Africa
Source: BMC Genomics. 2025 Apr 3;26:334. doi: 10.1186/s12864-025-11413-3 (PMC11966903; doi:10.1186/s12864-025-11413-3)
Supplement: Supplementary file 2 — Supplementary Material 2 [file 12864_2025_11413_MOESM2_ESM.docx]

**Table S2** Information on the origin of 205 international *Leptosphaeria maculans* isolates for which whole genome sequence data was downloaded from GenBank and included in the current study

| **Isolate number** | **Country** | **Region** | **Year** | **Closest town** | **Host cultivar** |
| --- | --- | --- | --- | --- | --- |
| IBCN094 | in vitro | NA | NA | NA | NA |
| IBCN095 | in vitro | N/A | NA | NA | NA |
| IBCN096 | Argentina | Buenos Aires | 2019 | Balcarce | unknown |
| IBCN097 | Argentina | Buenos Aires | 2019 | Balcarce | unknown |
| IBCN098 | Argentina | Buenos Aires | 2019 | General Madariaga | unknown |
| IBCN099 | Argentina | Buenos Aires | 2019 | General Madariaga | unknown |
| IBCN100 | Argentina | Buenos Aires | 2019 | General Madariaga | unknown |
| IBCN101 | Canada | Alberta | 2018 | Manning | Westar |
| IBCN102 | Canada | Alberta | 2018 | Manning | Westar |
| IBCN103 | Canada | Saskatchewan | 2018 | North Battleford | Westar |
| IBCN104 | Canada | Saskatchewan | 2018 | Watrous | Westar |
| IBCN105 | Canada | Manitoba | 2018 | Carman | Westar |
| IBCN106 | Canada | Manitoba | 2015 | Birtle | unknown |
| IBCN107 | Canada | Manitoba | 2015 | Dauphin | unknown |
| IBCN108 | Canada | Manitoba | 2015 | Pipestone | unknown |
| IBCN109 | Canada | Manitoba | 2015 | Hamiota | unknown |
| IBCN110 | Canada | Manitoba | 2015 | Plum Coulee | unknown |
| IBCN111 | Canada | Manitoba | 2015 | Dauphin | unknown |
| IBCN112 | Canada | Manitoba | 2015 | McCreary | unknown |
| IBCN113 | Canada | Manitoba | 2015 | Somerset | unknown |
| IBCN114 | Canada | Manitoba | 2015 | Dauphin | unknown |
| IBCN115 | Canada | Manitoba | 2009 | Stonewall | 9553 |
| IBCN116 | Canada | Saskatchewan | 2012 | Watrous | Westar |
| IBCN118 | Canada | Manitoba | 2012 | Miami | Westar |
| IBCN119 | Canada | Alberta | 2012 | Daysland | VT 500 G |
| IBCN120 | Canada | Alberta | 2012 | High River | 45S52 |
| IBCN125 | France | Hauts-de-France | 2017 | Foleville | Falcon |
| IBCN127 | France | Brittany | 2018 | Le Rheu | Westar |
| IBCN128 | France | Brittany | 2018 | Le Rheu | Westar |
| IBCN129 | France | Brittany | 2018 | Le Rheu | Westar |
| IBCN130 | France | Brittany | 2018 | Le Rheu | Westar |
| IBCN131 | France | Brittany | 2018 | Le Rheu | Westar |
| IBCN133 | France | Hauts-de-France | 2017 | Prémesque | Falcon |
| IBCN135 | France | Occitania | 2017 | Seilh | Falcon |
| IBCN136 | France | Hauts-de-France | 2017 | Prémesque | Falcon |
| IBCN137 | France | Centre-Val de Loire | 2017 | Blois | Falcon |
| IBCN138 | France | Pays de la Loire | 2013 | La Roche-sur-Yon | Pamela |
| IBCN139 | France | Ile de France | 2019 | Grignon | Topas |
| IBCN140 | France | Ile de France | 2019 | Grignon | Darmor-Rlm11 |
| IBCN141 | France | Ile de France | 2019 | Grignon | Darmor-Rlm11 |
| IBCN142 | France | Ile de France | 2019 | Grignon | Napoli |
| IBCN143 | New Zealand | Canterbury | 2011 | Makikihi | unknown |
| IBCN144 | New Zealand | Canterbury | 2011 | Makikihi | unknown |
| IBCN145 | New Zealand | Canterbury | 2011 | Lincoln | Ability |
| IBCN146 | New Zealand | Hawke's Bay | 2011 | Puketitiri | Dominion |
| IBCN147 | New Zealand | Hawke's Bay | 2011 | Puketitiri | Aparima |
| IBCN148 | New Zealand | Southland | 2011 | Gore | Aparima |
| IBCN149 | New Zealand | Southland | 2011 | Gore | Major Plus |
| IBCN150 | New Zealand | Canterbury | 2011 | Makikihi | unknown |
| IBCN151 | New Zealand | Canterbury | 2011 | Lincoln | Flash |
| IBCN152 | New Zealand | Canterbury | 2011 | Lincoln | Flash |
| IBCN153 | New Zealand | Canterbury | 2011 | Lincoln | Flash |
| IBCN154 | New Zealand | Canterbury | 2011 | Lincoln | unknown |
| IBCN155 | New Zealand | Canterbury | 2011 | Lincoln | Flash |
| IBCN156 | New Zealand | Canterbury | 2011 | Lincoln | Flash |
| IBCN157 | New Zealand | Canterbury | 2011 | Lincoln | Flash |
| IBCN158 | New Zealand | Southland | 2011 | Gore | Highlander |
| IBCN159 | New Zealand | Canterbury | 2011 | Darfield | Flash |
| IBCN160 | New Zealand | Hawke's Bay | 2011 | Puketitiri | Dominion |
| IBCN161 | New Zealand | Canterbury | 2011 | Darfield | unknown |
| IBCN162 | New Zealand | Canterbury | 2012 | Darfield | unknown |
| IBCN163 | New Zealand | Canterbury | 2012 | Darfield | unknown |
| IBCN164 | New Zealand | Canterbury | 2013 | Pleasant Point | Flash |
| IBCN165 | New Zealand | Canterbury | 2013 | Pleasant Point | Flash |
| IBCN166 | New Zealand | Canterbury | 2013 | Pleasant Point | Flash |
| IBCN167 | New Zealand | Canterbury | 2013 | Pleasant Point | Flash |
| IBCN168 | New Zealand | Canterbury | 2013 | Pleasant Point | Flash |
| IBCN169 | New Zealand | Canterbury | 2013 | Pleasant Point | Flash |
| IBCN170 | New Zealand | Canterbury | 2013 | Pleasant Point | Flash |
| IBCN172 | UK | Oxfordshire | 2011 | Banbury | Drakkar |
| IBCN173 | UK | Oxfordshire | 2011 | Banbury | Drakkar |
| IBCN174 | UK | Oxfordshire | 2011 | Banbury | Drakkar |
| IBCN175 | UK | Oxfordshire | 2011 | Banbury | Drakkar |
| IBCN176 | UK | Norfolk | 2011 | Wymondham | Drakkar |
| IBCN177 | UK | Norfolk | 2011 | Wymondham | Drakkar |
| IBCN178 | UK | Norfolk | 2010 | Wymondham | Drakkar |
| IBCN179 | UK | North Yorkshire | 2010 | Bainton | Drakkar |
| IBCN180 | UK | Hampshire | 2010 | Stockbridge | Drakkar |
| IBCN181 | UK | Hampshire | 2010 | Stockbridge | Drakkar |
| IBCN182 | UK | Suffolk | 2010 | Cowlinge | Drakkar |
| IBCN183 | UK | Hertfordshire | 2010 | Harpenden | Drakkar |
| IBCN184 | UK | Cambridgeshire | 2016 | Impington | Drakkar |
| IBCN185 | UK | Norfolk | 2016 | Wymondham | Harper |
| IBCN187 | UK | Norfolk | 2016 | Wymondham | Whisky |
| IBCN188 | UK | Norfolk | 2016 | Wymondham | Whisky |
| IBCN189 | UK | Norfolk | 2016 | Wymondham | Whisky |
| IBCN190 | UK | Cambridgeshire | 2016 | Wisbech | Harper |
| IBCN191 | UK | Hertfordshire | 2017 | Hertford | Angus |
| IBCN193 | UK | West Yorkshire | 2002 | Darrington | Apex |
| IBCN194 | USA | North Dakota | 2004 | Towner | unknown |
| IBCN195 | USA | North Dakota | 2004 | Cavalier | unknown |
| IBCN196 | USA | Georgia | 2005 | Griffin | unknown |
| IBCN197 | USA | Georgia | 2005 | Griffin | unknown |
| IBCN198 | USA | Georgia | 2005 | Plains | unknown |
| IBCN199 | USA | Georgia | 2006 | Plains | unknown |
| IBCN200 | USA | North Dakota | 2007 | Balfour | unknown |
| IBCN201 | USA | North Dakota | 2007 | Fesenden | unknown |
| IBCN202 | USA | North Dakota | 2007 | Fesenden | unknown |
| IBCN203 | USA | North Dakota | 2007 | Carrington | unknown |
| IBCN204 | USA | North Dakota | 2007 | Grand Forks | unknown |
| IBCN205 | USA | North Dakota | 2007 | Williston | unknown |
| IBCN206 | USA | North Dakota | 2007 | Towner | unknown |
| IBCN207 | USA | North Dakota | 2014 | Bottineau | unknown |
| IBCN208 | USA | North Dakota | 2014 | Walsh | unknown |
| IBCN209 | USA | North Dakota | 2016 | Cavalier | unknown |
| IBCN210 | USA | North Dakota | 2016 | Cavalier | unknown |
| IBCN211 | USA | North Dakota | 2016 | Cavalier | unknown |
| IBCN212 | USA | North Dakota | 2016 | Cavalier | unknown |
| IBCN213 | USA | North Dakota | 2016 | Cavalier | unknown |
| IBCN214 | USA | North Dakota | 2017 | Cavalier | unknown |
| IBCN215 | USA | North Dakota | 2017 | Cavalier | unknown |
| IBCN216 | USA | North Dakota | 2017 | Cavalier | unknown |
| IBCN217 | USA | North Dakota | 2018 | Rolette | unknown |
| IBCN218 | USA | North Dakota | 2017 | Walsh | unknown |
| IBCN224 | Iran | Mazandaran | 2021 | Behshahr 1 | unknown |
| IBCN237 | Iran | Mazandaran | 2021 | Rostamkola_Asiabsar 1 | unknown |
| IBCN238 | Iran | Mazandaran | 2021 | Rostamkola_Asiabsar 2 | unknown |
| IBCN239 | Iran | Mazandaran | 2021 | Behshahr 2 | unknown |
| IBCN240 | Iran | Mazandaran | 2021 | Behshahr 3 | unknown |
| IBCN241 | South Africa | Western Cape | 2020 | Moorreesburg | Hyola®50 |
| IBCN242 | South Africa | Western Cape | 2020 | Moorreesburg | Hyola®580 CT |
| IBCN243 | South Africa | Western Cape | 2020 | Moorreesburg | Hyola®580 CT |
| IBCN244 | South Africa | Western Cape | 2020 | Moorreesburg | NUSEED Diamond |
| IBCN245 | South Africa | Western Cape | 2020 | Moorreesburg | NUSEED Diamond |
| IBCN246 | South Africa | Western Cape | 2020 | Hopefield | Pioneer®43Y92CL |
| IBCN247 | South Africa | Western Cape | 2020 | Hopefield | Pioneer®43Y92CL |
| IBCN249 | South Africa | Western Cape | 2020 | Hopefield | Pioneer®45Y93CL |
| IBCN250 | South Africa | Western Cape | 2020 | Hopefield | CB-Tango |
| IBCN251 | South Africa | Western Cape | 2020 | Hopefield | CB Tango |
| IBCN252 | South Africa | Western Cape | 2020 | Riviersonderend | Alpha TT |
| IBCN253 | South Africa | Western Cape | 2020 | Riviersonderend | Alpha TT |
| IBCN254 | South Africa | Western Cape | 2020 | Riviersonderend | NUSEED Quartz |
| IBCN255 | South Africa | Western Cape | 2020 | Riviersonderend | NUSEED Quartz |
| IBCN256 | South Africa | Western Cape | 2020 | Riviersonderend | Pioneer®45Y91CL |
| IBCN257 | South Africa | Western Cape | 2020 | Riviersonderend | Pioneer®45Y91CL |
| IBCN258 | South Africa | Western Cape | 2020 | Riversdale | Pioneer®43Y92CL |
| IBCN259 | South Africa | Western Cape | 2020 | Riversdale | Pioneer®43Y92CL |
| IBCN260 | South Africa | Western Cape | 2020 | Riversdale | Hyola®559TT |
| IBCN261 | South Africa | Western Cape | 2020 | Riversdale | Hyola®559TT |
| IBCN262 | South Africa | Western Cape | 2021 | Moorreesburg | NUSEED Diamond |
| IBCN263 | South Africa | Western Cape | 2021 | Moorreesburg | NUSEED Diamond |
| IBCN264 | South Africa | Western Cape | 2021 | Moorreesburg | Pioneer®44Y94CL |
| IBCN265 | South Africa | Western Cape | 2021 | Moorreesburg | Pioneer®44Y94CL |
| IBCN266 | South Africa | Western Cape | 2021 | Eendekuil | Pioneer®45Y95CL |
| IBCN267 | South Africa | Western Cape | 2021 | Eendekuil | Pioneer®45Y95CL |
| IBCN268 | South Africa | Western Cape | 2021 | Eendekuil | Hyola®BlazerTT |
| IBCN269 | South Africa | Western Cape | 2021 | Eendekuil | Alpha TT |
| IBCN270 | South Africa | Western Cape | 2021 | Eendekuil | Alpha TT |
| IBCN271 | South Africa | Western Cape | 2021 | Eendekuil | NUSEED Quartz |
| IBCN272 | South Africa | Western Cape | 2021 | Eendekuil | NUSEED Quartz |
| IBCN273 | South Africa | Western Cape | 2021 | Napier | Hyola®650TT |
| IBCN274 | South Africa | Western Cape | 2021 | Napier | Hyola®650TT |
| IBCN275 | South Africa | Western Cape | 2021 | Hopefield | Pioneer®43Y92CL |
| IBCN276 | Germany | Lower Saxony | 2020 | Asendorf | Exocet |
| IBCN277 | Germany | Lower Saxony | 2019 | Peine | NK-Bravour |
| IBCN278 | Germany | Lower Saxony | 2018 | Einbeck | Exocet |
| IBCN279 | Germany | Lower Saxony | 2018 | Einbeck | NK-Bravour |
| IBCN280 | Germany | Lower Saxony | 2018 | Nienstädt | NK-Bravour |
| IBCN281 | Germany | Schleswig Holstein | 2019 | Sörup | NK-Bravour |
| IBCN282 | Germany | Schleswig Holstein | 2019 | Sörup | NK-Bravour |
| IBCN283 | Germany | Lower Saxony | 2020 | Asendorf | NK-Bravour |
| IBCN284 | Germany | Lower Saxony | 2018 | Nienstädt | NK-Bravour |
| IBCN285 | Germany | Lower Saxony | 2018 | Einbeck | NK-Bravour |
| IBCN286 | Germany | Lower Saxony | 2018 | Einbeck | NK-Bravour |
| IBCN288 | Germany | Lower Saxony | 2018 | Einbeck | NK-Bravour |
| IBCN289 | Germany | Lower Saxony | 2019 | Peine | NK-Bravour |
| IBCN290 | Germany | Mecklenburg-West Pomerania | 2019 | Groß Helle | NK-Bravour |
| IBCN291 | Germany | Mecklenburg-West Pomerania | 2018 | Groß Helle | NK-Bravour |
| IBCN292 | Germany | Mecklenburg-West Pomerania | 2018 | Groß Helle | NK-Bravour |
| IBCN293 | Germany | Lower Saxony | 2018 | Nienstädt | NK-Bravour |
| IBCN294 | Germany | Lower Saxony | 2018 | Nienstädt | NK-Bravour |
| IBCN295 | Germany | Lower Saxony | 2018 | Nienstädt | NK-Bravour |
| IBCN297 | Czech | Liberec | 2020 | Chrastava | Jet Neuf |
| IBCN299 | Czech | Karlovy Vary | 2019 | Krásné Údolí | Bristol |
| IBCN300 | Czech | Central Bohemian | 2017 | Velké Přílepy | unknown |
| IBCN301 | Czech | Olomouc | 2020 | Šumperk | Darmor |
| IBCN302 | Czech | Hradec Králové | 2017 | Nové Město u Chlumce nad Cidlinou | Westar |
| IBCN303 | Czech | Hradec Králové | 2020 | Trutnov | Jet Neuf |
| IBCN304 | Czech | Hradec Králové | 2020 | Hněvčeves | Darmor |
| IBCN305 | Czech | Opava | 2017 | Chvalíkovice | OP1 |
| IBCN306 | Australia | South Australia | 2005 | Yeelanna | Surpass603CL |
| IBCN307 | Australia | South Australia | 2006 | Yeelanna | ATR-Beacon |
| IBCN308 | Australia | South Australia | 2006 | Yeelanna | Surpass501TT |
| IBCN309 | Australia | South Australia | 2006 | Yeelanna | Surpass501TT |
| IBCN310 | Australia | Victoria | 2006 | Horsham | unknown |
| IBCN311 | Australia | South Australia | 2012 | Wangary | Hyola®575CL |
| IBCN312 | Australia | South Australia | 2013 | Wangary | CB-Tango |
| IBCN313 | Australia | South Australia | 2014 | Wangary | ThumperTT |
| IBCN314 | Australia | Western Australia | 2014 | Katanning | CB-AtomicHT |
| IBCN315 | Australia | Western Australia | 2016 | Katanning | CB-Telfer |
| IBCN316 | Australia | Western Australia | 2016 | Kojonup | CB-Telfer |
| IBCN317 | Australia | Victoria | 2016 | Kaniva | ATR-Marlin |
| IBCN318 | Australia | South Australia | 2018 | Bordertown | ATR-Marlin |
| IBCN319 | Australia | Victoria | 2018 | Laharum | ATR-Bonito |
| IBCN320 | Australia | New South Wales | 2018 | Marrar | Archer |
| IBCN321 | Australia | New South Wales | 2019 | Wagga Wagga | ATR-Bonito |
| IBCN322 | Australia | Western Australia | 2019 | Kojonup | BASF3000TR |
| IBCN323 | Australia | New South Wales | 2019 | Cowra | Hyola®559TT |
| IBCN324 | Australia | New South Wales | 2019 | Lockhart | ATR-Bonito |
| IBCN325 | Australia | New South Wales | 2020 | Wagga Wagga | ATR-Bonito |
| IBCN326 | Australia | Western Australia | 2020 | Gibson | BASF3000TR |
| IBCN327 | Australia | South Australia | 2020 | Eyre Peninsula | Hyola®50 |
| IBCN328 | Australia | Victoria | 2020 | Lake Bolac | Hyola®580 CT |
| IBCN329 | Australia | South Australia | 2020 | Cummins | DG BidgeeTT |
| IBCN330 | Australia | Victoria | 2020 | Corop | ATR-Bonito |

Information obtained from Van de Wouw *et al.* 2024 (20)
