# Supplementary material for: Whole genome sequencing reveals the genetic diversity and structure of Leptosphaeria maculans populations from the Western Cape province of South Africa
Source: BMC Genomics. 2025 Apr 3;26:334. doi: 10.1186/s12864-025-11413-3 (PMC11966903; doi:10.1186/s12864-025-11413-3)
Supplement: Supplementary file 3 — Supplementary Material 3 [file 12864_2025_11413_MOESM3_ESM.docx]

**Table S3** Whole genome sequencing statistics for 230 *Leptosphaeria maculans* isolates collected across the canola production regions of the Western Cape between 2020 and 2022

| **Isolate number** | **Number of raw read pairs** | **Amount of data (bp)** | **Number of read pairs after trimming** | **Data after trimming (bp)** | **Data retained (%)** | **Theoretical coverage (times)** | **Number of mapped reads** | **Mapped reads (%)** | **Number of reference genome bases covered** | **Breadth of coverage (%)** | **Number of variants called** |
| --- | --- | --- | --- | --- | --- | --- | --- | --- | --- | --- | --- |
| STE-U 9674 | 8 089 526 | 2 426 857 800 | 8 050 017 | 2 341 903 390 | 96,50 | 50,93 | 15 789 892 | 97,59 | 43 608 692 | 94,83 | 14119 |
| STE-U 9675 | 7 915 323 | 2 374 596 900 | 7 884 314 | 2 293 721 243 | 96,59 | 49,88 | 15 660 768 | 98,93 | 43 604 152 | 94,82 | 14055 |
| STE-U 9679 | 6 610 506 | 1 983 151 800 | 6 584 586 | 1 915 595 047 | 96,59 | 41,66 | 12 916 259 | 97,69 | 43 570 203 | 94,75 | 14021 |
| STE-U 9680 | 8 426 627 | 2 527 988 100 | 8 387 080 | 2 439 862 867 | 96,51 | 53,06 | 16 442 145 | 97,56 | 43 529 817 | 94,66 | 14067 |
| STE-U 9681 | 8 488 897 | 2 546 669 100 | 8 451 569 | 2 458 563 487 | 96,54 | 53,46 | 16 581 892 | 97,67 | 43 733 331 | 95,10 | 14066 |
| STE-U 9682 | 6 624 086 | 1 987 225 800 | 6 592 805 | 1 917 924 530 | 96,51 | 41,71 | 12 990 996 | 98,06 | 43 487 898 | 94,57 | 13992 |
| STE-U 9683 | 7 837 162 | 2 351 148 600 | 7 804 643 | 2 270 563 500 | 96,57 | 49,37 | 15 393 745 | 98,21 | 43 697 545 | 95,02 | 14140 |
| STE-U 9684 | 8 610 726 | 2 583 217 800 | 8 565 111 | 2 491 536 839 | 96,45 | 54,18 | 16 967 502 | 98,53 | 43 494 710 | 94,58 | 14071 |
| STE-U 9685 | 13 173 821 | 3 952 146 300 | 13 141 245 | 3 822 263 410 | 96,71 | 83,12 | 25 868 267 | 98,18 | 43 779 711 | 95,20 | 14095 |
| STE-U 9687 | 7 827 459 | 2 348 237 700 | 7 790 276 | 2 266 269 120 | 96,51 | 49,28 | 15 355 857 | 98,09 | 43 719 960 | 95,07 | 14111 |
| STE-U 9688 | 6 790 953 | 2 037 285 900 | 6 759 345 | 1 966 356 641 | 96,52 | 42,76 | 13 286 227 | 97,82 | 43 482 409 | 94,55 | 14076 |
| STE-U 9689 | 8 293 411 | 2 488 023 300 | 8 253 863 | 2 401 168 599 | 96,51 | 52,21 | 16 350 751 | 98,58 | 43 535 036 | 94,67 | 14110 |
| STE-U 9690 | 6 720 682 | 2 016 204 600 | 6 694 627 | 1 947 664 872 | 96,60 | 42,35 | 13 243 123 | 98,53 | 43 701 358 | 95,03 | 14045 |
| STE-U 9691 | 7 734 026 | 2 320 207 800 | 7 696 819 | 2 239 076 211 | 96,50 | 48,69 | 15 157 148 | 97,99 | 43 262 985 | 94,08 | 14076 |
| STE-U 9692 | 8 012 930 | 2 403 879 000 | 7 979 872 | 2 321 493 603 | 96,57 | 50,48 | 15 658 585 | 97,71 | 43 622 343 | 94,86 | 14021 |
| STE-U 9693 | 9 681 053 | 2 904 315 900 | 9 636 690 | 2 803 398 399 | 96,53 | 60,96 | 19 107 219 | 98,68 | 43 671 068 | 94,97 | 14041 |
| STE-U 9694 | 6 739 951 | 2 021 985 300 | 6 709 121 | 1 951 757 041 | 96,53 | 42,44 | 13 167 477 | 97,68 | 43 579 310 | 94,77 | 14057 |
| STE-U 9695 | 7 741 249 | 2 322 374 700 | 7 705 152 | 2 241 504 911 | 96,52 | 48,74 | 15 156 868 | 97,90 | 43 489 007 | 94,57 | 14022 |
| STE-U 9696 | 7 094 827 | 2 128 448 100 | 7 054 920 | 2 052 201 970 | 96,42 | 44,63 | 14 062 222 | 99,10 | 43 198 880 | 93,94 | 14011 |
| STE-U 9697 | 9 179 242 | 2 753 772 600 | 8 987 127 | 2 613 591 681 | 94,91 | 56,83 | 17 712 258 | 96,48 | 43 405 076 | 94,39 | 14050 |
| STE-U 9698 | 6 972 501 | 2 091 750 300 | 6 938 842 | 2 018 591 819 | 96,50 | 43,90 | 13 606 454 | 97,57 | 43 495 268 | 94,58 | 13999 |
| STE-U 9699 | 7 661 581 | 2 298 474 300 | 7 625 027 | 2 218 200 201 | 96,51 | 48,24 | 15 003 924 | 97,92 | 43 368 975 | 94,31 | 14037 |
| STE-U 9700 | 7 842 462 | 2 352 738 600 | 7 804 625 | 2 270 452 542 | 96,50 | 49,37 | 15 354 051 | 97,89 | 43 545 412 | 94,69 | 14020 |
| STE-U 9701 | 7 557 582 | 2 267 274 600 | 7 522 179 | 2 188 262 496 | 96,52 | 47,58 | 14 727 479 | 97,44 | 43 307 906 | 94,18 | 14174 |
| STE-U 9702 | 6 635 008 | 1 990 502 400 | 6 596 570 | 1 918 354 042 | 96,38 | 41,72 | 12 952 003 | 97,60 | 43 344 479 | 94,25 | 14111 |
| STE-U 9703 | 8 229 214 | 2 468 764 200 | 8 195 581 | 2 384 249 434 | 96,58 | 51,85 | 16 088 916 | 97,75 | 43 503 408 | 94,60 | 14105 |
| STE-U 9704 | 6 955 667 | 2 086 700 100 | 6 925 360 | 2 014 687 354 | 96,55 | 43,81 | 13 688 891 | 98,40 | 43 536 691 | 94,67 | 14039 |
| STE-U 9705 | 9 279 722 | 2 783 916 600 | 9 228 071 | 2 684 468 166 | 96,43 | 58,38 | 18 306 693 | 98,64 | 43 532 512 | 94,66 | 14041 |
| STE-U 9706 | 7 597 812 | 2 279 343 600 | 7 557 061 | 2 198 365 828 | 96,45 | 47,80 | 14 975 543 | 98,55 | 43 391 140 | 94,36 | 14026 |
| STE-U 9707 | 6 975 196 | 2 092 558 800 | 6 935 479 | 2 017 496 159 | 96,41 | 43,87 | 13 821 060 | 99,07 | 43 511 611 | 94,62 | 13991 |
| STE-U 9708 | 6 758 627 | 2 027 588 100 | 6 725 410 | 1 956 377 541 | 96,49 | 42,54 | 13 302 890 | 98,41 | 43 390 398 | 94,35 | 14027 |
| STE-U 9709 | 7 851 539 | 2 355 461 700 | 7 810 182 | 2 271 977 804 | 96,46 | 49,41 | 15 416 462 | 98,17 | 43 384 937 | 94,34 | 13941 |
| STE-U 9711 | 8 000 025 | 2 400 007 500 | 7 956 244 | 2 314 441 403 | 96,43 | 50,33 | 15 715 533 | 98,22 | 43 305 840 | 94,17 | 13998 |
| STE-U 9712 | 9 367 332 | 2 810 199 600 | 9 316 671 | 2 710 208 221 | 96,44 | 58,93 | 18 502 926 | 98,76 | 43 408 820 | 94,39 | 14023 |
| STE-U 9715 | 8 406 023 | 2 521 806 900 | 8 367 347 | 2 434 145 785 | 96,52 | 52,93 | 16 503 744 | 98,17 | 43 172 413 | 93,88 | 13947 |
| STE-U 9717 | 6 868 584 | 2 060 575 200 | 6 836 603 | 1 988 819 070 | 96,52 | 43,25 | 13 317 890 | 96,95 | 43 570 337 | 94,75 | 14046 |
| STE-U 9718 | 7 265 234 | 2 179 570 200 | 7 235 077 | 2 104 685 546 | 96,56 | 45,77 | 14 051 084 | 96,70 | 43 182 369 | 93,90 | 13969 |
| STE-U 9719 | 8 383 855 | 2 515 156 500 | 8 344 358 | 2 427 620 835 | 96,52 | 52,79 | 16 468 342 | 98,21 | 43 450 495 | 94,49 | 14014 |
| STE-U 9720 | 7 451 150 | 2 235 345 000 | 7 419 200 | 2 158 469 955 | 96,56 | 46,94 | 14 561 271 | 97,71 | 43 205 184 | 93,95 | 14038 |
| STE-U 9721 | 8 875 667 | 2 662 700 100 | 8 833 732 | 2 569 955 066 | 96,52 | 55,89 | 17 472 318 | 98,43 | 43 197 141 | 93,93 | 14051 |
| STE-U 9722 | 7 261 383 | 2 178 414 900 | 7 225 235 | 2 101 975 680 | 96,49 | 45,71 | 14 287 023 | 98,38 | 42 917 431 | 93,33 | 14045 |
| STE-U 9723 | 7 258 869 | 2 177 660 700 | 7 226 703 | 2 102 459 801 | 96,55 | 45,72 | 14 283 049 | 98,38 | 43 349 094 | 94,26 | 14034 |
| STE-U 9724 | 7 732 772 | 2 319 831 600 | 7 696 271 | 2 238 944 673 | 96,51 | 48,69 | 15 071 040 | 97,45 | 43 341 538 | 94,25 | 13975 |
| STE-U 9725 | 9 584 195 | 2 875 258 500 | 9 547 221 | 2 777 565 671 | 96,60 | 60,40 | 18 894 265 | 98,57 | 43 503 816 | 94,60 | 13973 |
| STE-U 9727 | 8 057 868 | 2 417 360 400 | 8 021 638 | 2 333 820 610 | 96,54 | 50,75 | 14 700 722 | 91,22 | 43 330 511 | 94,22 | 14074 |
| STE-U 9729 | 7 652 357 | 2 295 707 100 | 7 615 075 | 2 215 413 850 | 96,50 | 48,18 | 15 109 277 | 98,72 | 43 343 982 | 94,25 | 14056 |
| STE-U 9730 | 6 848 471 | 2 054 541 300 | 6 817 582 | 1 983 471 404 | 96,54 | 43,13 | 13 543 074 | 98,88 | 43 130 234 | 93,79 | 14063 |
| STE-U 9731 | 8 731 383 | 2 619 414 900 | 8 707 321 | 2 532 714 517 | 96,69 | 55,08 | 17 167 791 | 98,31 | 43 512 740 | 94,62 | 14061 |
| STE-U 9732 | 11 108 680 | 3 332 604 000 | 11 078 914 | 3 222 591 413 | 96,70 | 70,08 | 21 775 534 | 98,01 | 43 653 818 | 94,93 | 14000 |
| STE-U 9733 | 7 910 406 | 2 373 121 800 | 7 881 464 | 2 292 764 689 | 96,61 | 49,86 | 15 331 460 | 96,91 | 43 505 628 | 94,61 | 14021 |
| STE-U 9735 | 9 477 724 | 2 843 317 200 | 9 441 164 | 2 746 680 676 | 96,60 | 59,73 | 12 326 627 | 65,03 | 43 331 226 | 94,23 | 14045 |
| STE-U 9736 | 10 509 206 | 3 152 761 800 | 10 460 857 | 3 042 972 480 | 96,52 | 66,17 | 20 672 632 | 98,35 | 43 624 369 | 94,86 | 14125 |
| STE-U 9737 | 8 875 588 | 2 662 676 400 | 8 834 028 | 2 569 721 746 | 96,51 | 55,88 | 17 462 198 | 98,37 | 43 114 967 | 93,76 | 14130 |
| STE-U 9738 | 8 544 447 | 2 563 334 100 | 8 499 015 | 2 472 261 106 | 96,45 | 53,76 | 16 818 127 | 98,42 | 43 198 809 | 93,94 | 13963 |
| STE-U 9739 | 9 250 146 | 2 775 043 800 | 9 204 077 | 2 677 350 260 | 96,48 | 58,22 | 18 174 145 | 98,24 | 43 282 137 | 94,12 | 13969 |
| STE-U 9740 | 9 247 927 | 2 774 378 100 | 9 213 426 | 2 680 233 177 | 96,61 | 58,28 | 18 087 520 | 97,79 | 43 754 765 | 95,15 | 14022 |
| STE-U 9741 | 7 803 576 | 2 341 072 800 | 7 772 482 | 2 260 975 341 | 96,58 | 49,17 | 15 322 599 | 98,18 | 43 568 052 | 94,74 | 14026 |
| STE-U 9742 | 9 629 174 | 2 888 752 200 | 9 576 481 | 2 785 658 650 | 96,43 | 60,58 | 18 844 932 | 97,85 | 43 649 887 | 94,92 | 14026 |
| STE-U 9743 | 8 379 987 | 2 513 996 100 | 8 338 695 | 2 425 649 389 | 96,49 | 52,75 | 16 548 804 | 98,74 | 43 404 658 | 94,39 | 14020 |
| STE-U 9744 | 7 582 672 | 2 274 801 600 | 7 546 534 | 2 195 233 476 | 96,50 | 47,74 | 14 908 072 | 98,30 | 43 332 169 | 94,23 | 14024 |
| STE-U 9745 | 9 167 755 | 2 750 326 500 | 9 122 050 | 2 653 589 734 | 96,48 | 57,70 | 17 996 972 | 98,15 | 43 605 205 | 94,82 | 13965 |
| STE-U 9746 | 7 961 459 | 2 388 437 700 | 7 924 544 | 2 305 383 715 | 96,52 | 50,13 | 15 622 895 | 98,12 | 43 550 534 | 94,70 | 13998 |
| STE-U 9747 | 7 309 215 | 2 192 764 500 | 7 274 731 | 2 116 306 306 | 96,51 | 46,02 | 14 359 335 | 98,23 | 43 406 343 | 94,39 | 14033 |
| STE-U 9748 | 7 817 003 | 2 345 100 900 | 7 777 207 | 2 262 444 465 | 96,48 | 49,20 | 15 382 577 | 98,39 | 43 396 204 | 94,37 | 14056 |
| STE-U 9749 | 10 036 941 | 3 011 082 300 | 9 999 699 | 2 909 419 699 | 96,62 | 63,27 | 11 771 393 | 58,64 | 43 221 186 | 93,99 | 14058 |
| STE-U 9750 | 8 284 392 | 2 485 317 600 | 8 243 827 | 2 397 936 518 | 96,48 | 52,14 | 16 172 739 | 97,61 | 43 657 863 | 94,94 | 13947 |
| STE-U 9751 | 6 652 349 | 1 995 704 700 | 6 627 291 | 1 927 940 640 | 96,60 | 41,92 | 13 089 828 | 98,39 | 43 360 863 | 94,29 | 14041 |
| STE-U 9752 | 8 420 898 | 2 526 269 400 | 8 381 956 | 2 438 388 730 | 96,52 | 53,02 | 16 663 791 | 98,94 | 43 593 914 | 94,80 | 13986 |
| STE-U 9753 | 8 450 316 | 2 535 094 800 | 8 410 636 | 2 446 682 192 | 96,51 | 53,20 | 16 033 467 | 94,87 | 43 561 899 | 94,73 | 14073 |
| STE-U 9754 | 7 244 729 | 2 173 418 700 | 7 204 412 | 2 095 604 170 | 96,42 | 45,57 | 14 220 523 | 98,14 | 43 382 387 | 94,34 | 14071 |
| STE-U 9755 | 7 542 451 | 2 262 735 300 | 7 506 875 | 2 183 820 080 | 96,51 | 47,49 | 14 827 470 | 98,29 | 43 435 826 | 94,45 | 14045 |
| STE-U 9756 | 6 835 569 | 2 050 670 700 | 6 813 778 | 1 982 350 077 | 96,67 | 43,11 | 6 791 058 | 49,67 | 43 249 455 | 94,05 | 14013 |
| STE-U 9757 | 9 714 328 | 2 914 298 400 | 9 667 669 | 2 812 313 363 | 96,50 | 61,16 | 19 075 345 | 98,18 | 43 535 885 | 94,67 | 14006 |
| STE-U 9758 | 10 043 360 | 3 013 008 000 | 9 998 403 | 2 908 540 323 | 96,53 | 63,25 | 19 766 026 | 98,40 | 43 679 119 | 94,98 | 14059 |
| STE-U 9759 | 12 199 619 | 3 659 885 700 | 12 136 764 | 3 530 519 991 | 96,47 | 76,77 | 23 948 762 | 98,15 | 43 656 495 | 94,93 | 14011 |
| STE-U 9760 | 9 354 642 | 2 806 392 600 | 9 320 203 | 2 711 398 603 | 96,62 | 58,96 | 17 590 785 | 94,02 | 43 736 422 | 95,11 | 14067 |
| STE-U 9761 | 8 119 177 | 2 435 753 100 | 8 080 434 | 2 350 618 238 | 96,50 | 51,12 | 15 910 908 | 97,98 | 43 603 672 | 94,82 | 14074 |
| STE-U 9762 | 7 989 130 | 2 396 739 000 | 7 949 678 | 2 312 588 468 | 96,49 | 50,29 | 15 687 660 | 98,18 | 43 398 174 | 94,37 | 13966 |
| STE-U 9763 | 7 553 986 | 2 266 195 800 | 7 519 962 | 2 187 754 476 | 96,54 | 47,57 | 14 745 000 | 97,60 | 43 642 226 | 94,90 | 14085 |
| STE-U 9765 | 8 278 253 | 2 483 475 900 | 8 243 211 | 2 398 231 163 | 96,57 | 52,15 | 16 229 700 | 98,03 | 43 575 663 | 94,76 | 14115 |
| STE-U 9766 | 9 709 351 | 2 912 805 300 | 9 666 182 | 2 812 140 973 | 96,54 | 61,15 | 19 038 466 | 98,04 | 43 779 539 | 95,20 | 14079 |
| STE-U 9767 | 7 274 619 | 2 182 385 700 | 7 242 886 | 2 107 131 048 | 96,55 | 45,82 | 14 245 268 | 97,91 | 43 610 871 | 94,83 | 14035 |
| STE-U 9768 | 9 359 862 | 2 807 958 600 | 9 309 209 | 2 708 234 875 | 96,45 | 58,89 | 18 444 565 | 98,53 | 43 497 226 | 94,59 | 14014 |
| STE-U 9769 | 7 094 550 | 2 128 365 000 | 7 064 010 | 2 055 083 659 | 96,56 | 44,69 | 13 994 066 | 98,63 | 43 424 351 | 94,43 | 14051 |
| STE-U 9770 | 9 004 664 | 2 701 399 200 | 8 963 209 | 2 607 514 441 | 96,52 | 56,70 | 17 668 905 | 98,11 | 43 677 807 | 94,98 | 13993 |
| STE-U 9771 | 9 741 280 | 2 922 384 000 | 9 702 374 | 2 822 720 570 | 96,59 | 61,38 | 19 029 533 | 97,67 | 43 685 178 | 95,00 | 14092 |
| STE-U 9772 | 8 272 440 | 2 481 732 000 | 8 232 012 | 2 394 779 794 | 96,50 | 52,08 | 16 324 405 | 98,67 | 43 513 273 | 94,62 | 14029 |
| STE-U 9773 | 8 322 703 | 2 496 810 900 | 8 290 774 | 2 411 859 845 | 96,60 | 52,45 | 16 355 560 | 98,26 | 43 600 292 | 94,81 | 14082 |
| STE-U 9774 | 9 239 034 | 2 771 710 200 | 9 195 780 | 2 675 176 778 | 96,52 | 58,17 | 18 238 913 | 98,71 | 43 582 174 | 94,77 | 14078 |
| STE-U 9775 | 8 939 563 | 2 681 868 900 | 8 897 923 | 2 588 538 790 | 96,52 | 56,29 | 17 572 700 | 98,29 | 43 583 300 | 94,77 | 14079 |
| STE-U 9776 | 12 016 072 | 3 604 821 600 | 11 970 746 | 3 482 611 151 | 96,61 | 75,73 | 14 187 498 | 59,04 | 43 166 501 | 93,87 | 13984 |
| STE-U 9777 | 8 157 822 | 2 447 346 600 | 8 143 364 | 2 369 600 816 | 96,82 | 51,53 | 3 505 064 | 21,48 | 41 983 445 | 91,30 | 14034 |
| STE-U 9778 | 8 602 562 | 2 580 768 600 | 8 558 287 | 2 489 677 672 | 96,47 | 54,14 | 17 007 050 | 98,85 | 43 293 428 | 94,14 | 14025 |
| STE-U 9779 | 6 851 442 | 2 055 432 600 | 6 818 755 | 1 983 659 803 | 96,51 | 43,14 | 13 553 605 | 98,91 | 43 398 693 | 94,37 | 14060 |
| STE-U 9780 | 9 512 006 | 2 853 601 800 | 9 465 707 | 2 753 568 531 | 96,49 | 59,88 | 18 751 485 | 98,57 | 43 621 866 | 94,86 | 14069 |
| STE-U 9781 | 9 089 974 | 2 726 992 200 | 9 046 186 | 2 631 414 993 | 96,50 | 57,22 | 17 884 733 | 98,38 | 43 616 507 | 94,85 | 14082 |
| STE-U 9782 | 9 342 314 | 2 802 694 200 | 9 305 978 | 2 707 141 952 | 96,59 | 58,87 | 18 355 819 | 98,24 | 43 740 597 | 95,12 | 14058 |
| STE-U 9783 | 8 802 022 | 2 640 606 600 | 8 760 261 | 2 548 187 733 | 96,50 | 55,41 | 17 171 151 | 97,54 | 43 740 022 | 95,11 | 14009 |
| STE-U 9785 | 9 276 822 | 2 783 046 600 | 9 231 732 | 2 685 296 322 | 96,49 | 58,39 | 18 163 780 | 97,90 | 43 384 482 | 94,34 | 14010 |
| STE-U 9786 | 8 693 185 | 2 607 955 500 | 8 651 368 | 2 516 486 917 | 96,49 | 54,72 | 17 135 659 | 98,56 | 43 521 498 | 94,64 | 13988 |
| STE-U 9787 | 8 850 698 | 2 655 209 400 | 8 809 319 | 2 562 628 350 | 96,51 | 55,73 | 17 442 230 | 98,54 | 43 571 760 | 94,75 | 14048 |
| STE-U 9789 | 10 406 601 | 3 121 980 300 | 10 358 486 | 3 013 383 249 | 96,52 | 65,53 | 20 570 444 | 98,83 | 43 372 908 | 94,32 | 13978 |
| STE-U 9790 | 10 781 725 | 3 234 517 500 | 10 732 151 | 3 122 053 226 | 96,52 | 67,89 | 21 245 968 | 98,53 | 43 571 991 | 94,75 | 14049 |
| STE-U 9791 | 16 402 303 | 4 920 690 900 | 16 348 231 | 4 756 078 690 | 96,65 | 103,42 | 32 280 491 | 98,40 | 43 466 409 | 94,52 | 13984 |
| STE-U 9792 | 7 509 643 | 2 252 892 900 | 7 476 089 | 2 174 905 240 | 96,54 | 47,29 | 14 780 623 | 98,41 | 43 345 262 | 94,26 | 14003 |
| STE-U 9793 | 7 569 834 | 2 270 950 200 | 7 534 476 | 2 191 911 602 | 96,52 | 47,66 | 14 841 140 | 98,03 | 43 325 886 | 94,21 | 14011 |
| STE-U 9794 | 8 482 723 | 2 544 816 900 | 8 447 643 | 2 457 619 698 | 96,57 | 53,44 | 16 719 930 | 98,55 | 43 268 715 | 94,09 | 13989 |
| STE-U 9795 | 7 896 030 | 2 368 809 000 | 7 862 664 | 2 287 425 804 | 96,56 | 49,74 | 15 603 785 | 98,81 | 43 268 856 | 94,09 | 14015 |
| STE-U 9796 | 7 340 711 | 2 202 213 300 | 7 312 218 | 2 127 283 388 | 96,60 | 46,26 | 14 499 119 | 98,76 | 43 066 553 | 93,65 | 13976 |
| STE-U 9797 | 9 832 552 | 2 949 765 600 | 9 798 524 | 2 850 550 737 | 96,64 | 61,99 | 19 413 385 | 98,72 | 43 546 994 | 94,70 | 14033 |
| STE-U 9798 | 7 746 630 | 2 323 989 000 | 7 720 998 | 2 246 250 136 | 96,65 | 48,85 | 15 266 945 | 98,54 | 43 429 132 | 94,44 | 14030 |
| STE-U 9799 | 10 637 367 | 3 191 210 100 | 10 592 763 | 3 081 682 009 | 96,57 | 67,01 | 21 017 459 | 98,79 | 43 338 476 | 94,24 | 14119 |
| STE-U 9800 | 10 640 394 | 3 192 118 200 | 10 597 774 | 3 083 146 689 | 96,59 | 67,04 | 20 937 594 | 98,39 | 43 075 503 | 93,67 | 13969 |
| STE-U 9801 | 8 688 171 | 2 606 451 300 | 8 649 334 | 2 516 109 705 | 96,53 | 54,71 | 17 121 190 | 98,53 | 43 405 608 | 94,39 | 14009 |
| STE-U 9802 | 8 391 036 | 2 517 310 800 | 8 242 339 | 2 397 089 133 | 95,22 | 52,13 | 16 122 421 | 96,07 | 43 427 628 | 94,44 | 14031 |
| STE-U 9803 | 9 673 977 | 2 902 193 100 | 9 630 316 | 2 801 603 746 | 96,53 | 60,92 | 18 931 452 | 97,85 | 43 438 633 | 94,46 | 13944 |
| STE-U 9804 | 9 786 256 | 2 935 876 800 | 9 744 916 | 2 834 892 452 | 96,56 | 61,65 | 19 205 776 | 98,13 | 43 582 721 | 94,77 | 14059 |
| STE-U 9805 | 10 055 229 | 3 016 568 700 | 10 010 051 | 2 911 996 642 | 96,53 | 63,32 | 19 773 882 | 98,33 | 43 669 881 | 94,96 | 14013 |
| STE-U 9806 | 11 207 846 | 3 362 353 800 | 11 160 981 | 3 246 889 698 | 96,57 | 70,61 | 21 991 853 | 98,11 | 43 561 194 | 94,73 | 14061 |
| STE-U 9807 | 8 114 958 | 2 434 487 400 | 8 016 102 | 2 331 052 286 | 95,75 | 50,69 | 15 782 495 | 97,24 | 43 415 981 | 94,41 | 14027 |
| STE-U 9808 | 11 357 727 | 3 407 318 100 | 11 310 640 | 3 290 324 168 | 96,57 | 71,55 | 22 283 095 | 98,10 | 43 686 159 | 95,00 | 14082 |
| STE-U 9809 | 9 511 229 | 2 853 368 700 | 9 473 504 | 2 755 978 449 | 96,59 | 59,93 | 18 824 336 | 98,96 | 43 615 313 | 94,84 | 14018 |
| STE-U 9810 | 9 255 688 | 2 776 706 400 | 9 214 572 | 2 680 612 913 | 96,54 | 58,29 | 18 224 006 | 98,45 | 43 611 556 | 94,84 | 14076 |
| STE-U 9811 | 9 998 342 | 2 999 502 600 | 9 954 139 | 2 895 698 919 | 96,54 | 62,97 | 19 645 949 | 98,25 | 43 506 732 | 94,61 | 14005 |
| STE-U 9812 | 10 058 472 | 3 017 541 600 | 10 007 881 | 2 911 262 516 | 96,48 | 63,31 | 19 765 618 | 98,25 | 43 270 795 | 94,09 | 14032 |
| STE-U 9813 | 8 913 982 | 2 674 194 600 | 8 875 397 | 2 581 893 724 | 96,55 | 56,14 | 17 529 210 | 98,32 | 43 418 284 | 94,42 | 14045 |
| STE-U 9814 | 10 268 780 | 3 080 634 000 | 10 223 049 | 2 973 950 160 | 96,54 | 64,67 | 20 235 030 | 98,53 | 43 394 348 | 94,36 | 14042 |
| STE-U 9815 | 10 359 191 | 3 107 757 300 | 10 319 973 | 3 002 224 610 | 96,60 | 65,28 | 20 402 687 | 98,48 | 43 559 272 | 94,72 | 14004 |
| STE-U 9816 | 7 309 854 | 2 192 956 200 | 7 286 470 | 2 119 910 802 | 96,67 | 46,10 | 14 345 702 | 98,13 | 43 547 999 | 94,70 | 14015 |
| STE-U 9817 | 10 892 708 | 3 267 812 400 | 10 848 468 | 3 155 966 047 | 96,58 | 68,63 | 21 429 205 | 98,36 | 43 447 273 | 94,48 | 13971 |
| STE-U 9818 | 9 685 014 | 2 905 504 200 | 9 644 134 | 2 805 579 703 | 96,56 | 61,01 | 18 994 152 | 98,06 | 43 446 767 | 94,48 | 13987 |
| STE-U 9819 | 7 637 551 | 2 291 265 300 | 7 615 325 | 2 215 631 626 | 96,70 | 48,18 | 14 889 352 | 97,47 | 43 434 987 | 94,45 | 14012 |
| STE-U 9820 | 8 013 660 | 2 404 098 000 | 7 983 875 | 2 322 696 179 | 96,61 | 50,51 | 15 747 169 | 98,25 | 43 400 799 | 94,38 | 14087 |
| STE-U 9821 | 6 945 217 | 2 083 565 100 | 6 922 045 | 2 013 873 115 | 96,66 | 43,79 | 13 641 998 | 98,21 | 43 355 662 | 94,28 | 14027 |
| STE-U 9822 | 9 024 288 | 2 707 286 400 | 8 988 777 | 2 614 931 204 | 96,59 | 56,86 | 17 736 744 | 98,27 | 43 384 437 | 94,34 | 14092 |
| STE-U 9823 | 6 639 854 | 1 991 956 200 | 6 613 923 | 1 924 172 494 | 96,60 | 41,84 | 13 079 260 | 98,49 | 42 983 338 | 93,47 | 14021 |
| STE-U 9824 | 9 525 569 | 2 857 670 700 | 9 484 096 | 2 758 907 807 | 96,54 | 59,99 | 18 668 092 | 97,99 | 43 379 433 | 94,33 | 14027 |
| STE-U 9825 | 10 444 424 | 3 133 327 200 | 10 393 159 | 3 023 268 964 | 96,49 | 65,74 | 20 400 836 | 97,66 | 43 051 373 | 93,62 | 14011 |
| STE-U 9827 | 12 433 710 | 3 730 113 000 | 12 382 058 | 3 602 084 556 | 96,57 | 78,33 | 24 524 024 | 98,62 | 43 658 568 | 94,94 | 13978 |
| STE-U 9828 | 10 219 505 | 3 065 851 500 | 10 172 172 | 2 959 115 704 | 96,52 | 64,35 | 20 100 882 | 98,35 | 43 417 725 | 94,41 | 14062 |
| STE-U 9829 | 9 166 915 | 2 750 074 500 | 9 126 136 | 2 654 870 560 | 96,54 | 57,73 | 18 097 945 | 98,71 | 43 448 614 | 94,48 | 14003 |
| STE-U 9830 | 8 551 897 | 2 565 569 100 | 8 513 594 | 2 476 659 835 | 96,53 | 53,86 | 16 744 441 | 97,90 | 43 344 692 | 94,26 | 14011 |
| STE-U 9831 | 10 607 439 | 3 182 231 700 | 10 558 894 | 3 071 606 824 | 96,52 | 66,79 | 20 837 999 | 98,22 | 43 653 589 | 94,93 | 14098 |
| STE-U 9832 | 10 775 994 | 3 232 798 200 | 10 728 301 | 3 120 931 816 | 96,54 | 67,87 | 21 224 861 | 98,48 | 43 577 991 | 94,76 | 14048 |
| STE-U 9833 | 10 325 843 | 3 097 752 900 | 10 285 649 | 2 992 248 455 | 96,59 | 65,07 | 20 359 718 | 98,59 | 43 545 906 | 94,69 | 14073 |
| STE-U 9834 | 9 943 374 | 2 983 012 200 | 9 904 071 | 2 881 138 737 | 96,58 | 62,65 | 19 560 312 | 98,36 | 43 586 095 | 94,78 | 14070 |
| STE-U 9836 | 8 594 648 | 2 578 394 400 | 8 556 609 | 2 489 148 760 | 96,54 | 54,13 | 16 951 485 | 98,62 | 43 317 635 | 94,20 | 14068 |
| STE-U 9837 | 9 445 003 | 2 833 500 900 | 9 405 377 | 2 736 077 120 | 96,56 | 59,50 | 18 586 105 | 98,39 | 43 410 874 | 94,40 | 14009 |
| STE-U 9838 | 10 291 871 | 3 087 561 300 | 10 236 506 | 2 977 812 973 | 96,45 | 64,75 | 20 314 497 | 98,69 | 43 238 849 | 94,03 | 14026 |
| STE-U 9839 | 9 887 083 | 2 966 124 900 | 9 842 128 | 2 863 111 578 | 96,53 | 62,26 | 19 320 417 | 97,71 | 43 410 367 | 94,40 | 14034 |
| STE-U 9840 | 9 846 539 | 2 953 961 700 | 9 806 744 | 2 852 898 483 | 96,58 | 62,04 | 19 377 257 | 98,40 | 43 649 336 | 94,92 | 14045 |
| STE-U 9841 | 6 849 839 | 2 054 951 700 | 6 825 771 | 1 985 800 842 | 96,63 | 43,18 | 13 454 390 | 98,21 | 43 582 458 | 94,77 | 14032 |
| STE-U 9842 | 6 515 041 | 1 954 512 300 | 6 485 524 | 1 886 774 867 | 96,53 | 41,03 | 12 817 615 | 98,37 | 43 354 731 | 94,28 | 14049 |
| STE-U 9843 | 8 844 242 | 2 653 272 600 | 8 814 430 | 2 564 300 945 | 96,65 | 55,76 | 17 455 546 | 98,68 | 43 726 767 | 95,09 | 14073 |
| STE-U 9844 | 9 166 022 | 2 749 806 600 | 9 131 878 | 2 656 619 494 | 96,61 | 57,77 | 18 027 921 | 98,34 | 43 637 363 | 94,89 | 13990 |
| STE-U 9845 | 7 625 279 | 2 287 583 700 | 7 597 035 | 2 210 105 188 | 96,61 | 48,06 | 14 795 331 | 97,02 | 43 444 459 | 94,47 | 14047 |
| STE-U 9846 | 7 855 072 | 2 356 521 600 | 7 827 047 | 2 277 142 918 | 96,63 | 49,52 | 15 401 919 | 98,04 | 43 427 079 | 94,43 | 14018 |
| STE-U 9847 | 9 337 602 | 2 801 280 600 | 9 305 379 | 2 707 059 289 | 96,64 | 58,87 | 18 337 305 | 98,19 | 43 636 356 | 94,89 | 14039 |
| STE-U 9848 | 8 261 327 | 2 478 398 100 | 8 228 928 | 2 393 886 941 | 96,59 | 52,06 | 16 135 281 | 97,66 | 43 581 263 | 94,77 | 14057 |
| STE-U 9849 | 10 523 848 | 3 157 154 400 | 10 491 820 | 3 052 504 788 | 96,69 | 66,38 | 20 655 234 | 98,14 | 43 726 519 | 95,09 | 14043 |
| STE-U 9850 | 9 793 116 | 2 937 934 800 | 9 756 880 | 2 838 571 261 | 96,62 | 61,73 | 19 217 766 | 98,12 | 43 605 930 | 94,82 | 14045 |
| STE-U 9851 | 9 822 697 | 2 946 809 100 | 9 787 041 | 2 847 193 707 | 96,62 | 61,91 | 19 265 991 | 98,07 | 43 631 636 | 94,88 | 14112 |
| STE-U 9852 | 7 926 827 | 2 378 048 100 | 7 895 657 | 2 297 027 965 | 96,59 | 49,95 | 15 609 420 | 98,46 | 43 444 469 | 94,47 | 14037 |
| STE-U 9854 | 8 123 848 | 2 437 154 400 | 8 092 632 | 2 354 358 584 | 96,60 | 51,20 | 16 003 455 | 98,50 | 43 481 092 | 94,55 | 14025 |
| STE-U 9855 | 10 540 115 | 3 162 034 500 | 10 499 958 | 3 054 717 562 | 96,61 | 66,43 | 20 686 785 | 98,13 | 43 696 404 | 95,02 | 14060 |
| STE-U 9856 | 8 365 685 | 2 509 705 500 | 8 334 785 | 2 424 877 605 | 96,62 | 52,73 | 16 453 177 | 98,34 | 43 512 108 | 94,62 | 14148 |
| STE-U 9857 | 6 999 430 | 2 099 829 000 | 6 974 414 | 2 029 094 548 | 96,63 | 44,12 | 13 745 982 | 98,19 | 43 360 761 | 94,29 | 14063 |
| STE-U 9858 | 7 703 680 | 2 311 104 000 | 7 676 837 | 2 233 103 224 | 96,62 | 48,56 | 15 092 943 | 97,96 | 43 754 454 | 95,15 | 14073 |
| STE-U 9859 | 7 319 965 | 2 195 989 500 | 7 293 049 | 2 121 738 747 | 96,62 | 46,14 | 14 404 574 | 98,39 | 43 536 075 | 94,67 | 14048 |
| STE-U 9860 | 8 798 692 | 2 639 607 600 | 8 765 584 | 2 550 110 601 | 96,61 | 55,45 | 17 330 799 | 98,49 | 43 521 241 | 94,64 | 13962 |
| STE-U 9861 | 11 388 015 | 3 416 404 500 | 11 352 258 | 3 302 836 670 | 96,68 | 71,82 | 22 376 345 | 98,25 | 43 643 922 | 94,91 | 14040 |
| STE-U 9862 | 10 264 224 | 3 079 267 200 | 10 226 670 | 2 975 185 979 | 96,62 | 64,70 | 19 888 734 | 96,88 | 43 668 440 | 94,96 | 14070 |
| STE-U 9863 | 11 851 438 | 3 555 431 400 | 11 805 092 | 3 434 391 193 | 96,60 | 74,68 | 23 428 619 | 98,84 | 44 017 203 | 95,72 | 13981 |
| STE-U 9864 | 7 039 719 | 2 111 915 700 | 7 016 232 | 2 041 222 669 | 96,65 | 44,39 | 13 250 573 | 94,11 | 43 398 156 | 94,37 | 14057 |
| STE-U 9865 | 9 038 499 | 2 711 549 700 | 9 007 130 | 2 620 430 607 | 96,64 | 56,98 | 17 742 430 | 98,15 | 43 440 877 | 94,46 | 14057 |
| STE-U 9866 | 9 981 621 | 2 994 486 300 | 9 945 131 | 2 893 134 018 | 96,62 | 62,91 | 19 612 877 | 98,24 | 43 806 105 | 95,26 | 14089 |
| STE-U 9867 | 7 938 732 | 2 381 619 600 | 7 913 641 | 2 302 089 256 | 96,66 | 50,06 | 15 453 056 | 97,33 | 43 700 757 | 95,03 | 13964 |
| STE-U 9868 | 7 687 583 | 2 306 274 900 | 7 664 327 | 2 229 682 143 | 96,68 | 48,49 | 14 919 498 | 97,04 | 43 468 761 | 94,53 | 13978 |
| STE-U 9869 | 7 726 465 | 2 317 939 500 | 7 702 164 | 2 240 631 532 | 96,66 | 48,72 | 14 998 890 | 97,06 | 43 605 802 | 94,82 | 14001 |
| STE-U 9870 | 8 231 721 | 2 469 516 300 | 8 202 757 | 2 386 321 655 | 96,63 | 51,89 | 16 140 266 | 98,04 | 43 477 071 | 94,54 | 14032 |
| STE-U 9871 | 8 367 167 | 2 510 150 100 | 8 340 048 | 2 426 302 526 | 96,66 | 52,76 | 16 351 054 | 97,71 | 43 643 776 | 94,91 | 14044 |
| STE-U 9872 | 9 891 733 | 2 967 519 900 | 9 856 795 | 2 867 480 588 | 96,63 | 62,35 | 19 309 876 | 97,61 | 43 698 672 | 95,03 | 14043 |
| STE-U 9873 | 7 615 444 | 2 284 633 200 | 7 588 084 | 2 207 520 057 | 96,62 | 48,00 | 14 952 414 | 98,17 | 43 362 335 | 94,29 | 14026 |
| STE-U 9874 | 9 008 390 | 2 702 517 000 | 8 974 808 | 2 610 981 524 | 96,61 | 56,78 | 17 668 059 | 98,06 | 43 360 298 | 94,29 | 14061 |
| STE-U 9875 | 6 516 307 | 1 954 892 100 | 6 495 318 | 1 889 603 598 | 96,66 | 41,09 | 12 741 831 | 97,77 | 43 494 907 | 94,58 | 13981 |
| STE-U 9876 | 11 073 560 | 3 322 068 000 | 11 038 918 | 3 211 479 285 | 96,67 | 69,84 | 21 401 020 | 96,63 | 43 747 015 | 95,13 | 14011 |
| STE-U 9880 | 9 260 786 | 2 778 235 800 | 9 229 739 | 2 684 917 077 | 96,64 | 58,38 | 18 005 044 | 97,21 | 43 627 254 | 94,87 | 14047 |
| STE-U 9881 | 11 115 326 | 3 334 597 800 | 11 081 120 | 3 223 761 735 | 96,68 | 70,10 | 21 731 071 | 97,75 | 43 808 847 | 95,26 | 14109 |
| STE-U 9882 | 11 250 781 | 3 375 234 300 | 11 216 178 | 3 263 077 873 | 96,68 | 70,96 | 21 949 381 | 97,55 | 43 640 337 | 94,90 | 14028 |
| STE-U 9883 | 13 892 236 | 4 167 670 800 | 13 853 228 | 4 030 344 037 | 96,70 | 87,64 | 27 320 926 | 98,33 | 43 757 174 | 95,15 | 14021 |
| STE-U 9884 | 10 314 377 | 3 094 313 100 | 10 274 731 | 2 988 898 846 | 96,59 | 65,00 | 20 272 476 | 98,27 | 43 263 511 | 94,08 | 14091 |
| STE-U 9885 | 12 403 962 | 3 721 188 600 | 12 361 551 | 3 596 222 162 | 96,64 | 78,20 | 24 343 239 | 98,13 | 43 780 963 | 95,20 | 13997 |
| STE-U 9886 | 7 430 380 | 2 229 114 000 | 7 405 374 | 2 154 271 820 | 96,64 | 46,85 | 14 783 331 | 99,48 | 43 446 737 | 94,48 | 14094 |
| STE-U 9887 | 6 650 487 | 1 995 146 100 | 6 631 619 | 1 929 337 538 | 96,70 | 41,95 | 13 010 431 | 97,82 | 43 627 955 | 94,87 | 14012 |
| STE-U 9888 | 9 794 843 | 2 938 452 900 | 9 762 884 | 2 840 025 458 | 96,65 | 61,76 | 19 058 399 | 97,29 | 43 576 892 | 94,76 | 14118 |
| STE-U 9889 | 7 171 546 | 2 151 463 800 | 7 148 531 | 2 079 592 651 | 96,66 | 45,22 | 13 859 089 | 96,63 | 43 706 636 | 95,04 | 14137 |
| STE-U 9890 | 6 809 183 | 2 042 754 900 | 6 786 440 | 1 974 340 345 | 96,65 | 42,93 | 13 244 669 | 97,26 | 43 273 448 | 94,10 | 14082 |
| STE-U 9891 | 7 263 414 | 2 179 024 200 | 7 240 600 | 2 106 439 088 | 96,67 | 45,81 | 14 246 048 | 98,07 | 43 680 951 | 94,99 | 14071 |
| STE-U 9892 | 10 259 996 | 3 077 998 800 | 10 221 707 | 2 973 449 444 | 96,60 | 64,66 | 20 198 103 | 98,43 | 43 553 453 | 94,71 | 14030 |
| STE-U 9893 | 19 340 869 | 5 802 260 700 | 19 273 154 | 5 607 032 756 | 96,64 | 121,93 | 37 979 713 | 98,19 | 43 845 550 | 95,34 | 14096 |
| STE-U 9894 | 6 880 163 | 2 064 048 900 | 6 860 235 | 1 995 786 309 | 96,69 | 43,40 | 13 467 688 | 97,87 | 43 422 803 | 94,43 | 14057 |
| STE-U 9896 | 10 296 244 | 3 088 873 200 | 10 259 265 | 2 984 601 636 | 96,62 | 64,90 | 20 169 756 | 97,95 | 43 650 544 | 94,92 | 14054 |
| STE-U 9897 | 10 848 123 | 3 254 436 900 | 10 821 099 | 3 148 083 649 | 96,73 | 68,46 | 21 022 055 | 96,89 | 43 732 808 | 95,10 | 14007 |
| STE-U 9898 | 11 129 127 | 3 338 738 100 | 11 092 836 | 3 227 139 597 | 96,66 | 70,18 | 21 764 284 | 97,78 | 43 978 501 | 95,63 | 14032 |
| STE-U 9899 | 8 863 753 | 2 659 125 900 | 8 837 188 | 2 570 937 803 | 96,68 | 55,91 | 17 191 089 | 96,97 | 43 366 474 | 94,30 | 14031 |
| STE-U 9900 | 10 335 322 | 3 100 596 600 | 10 295 999 | 2 995 069 768 | 96,60 | 65,13 | 20 171 069 | 97,58 | 43 493 594 | 94,58 | 13920 |
| STE-U 9901 | 7 101 730 | 2 130 519 000 | 7 082 280 | 2 060 369 506 | 96,71 | 44,80 | 13 836 682 | 97,42 | 43 624 361 | 94,86 | 14055 |
| STE-U 9902 | 9 211 810 | 2 763 543 000 | 9 176 387 | 2 669 516 159 | 96,60 | 58,05 | 18 066 636 | 98,06 | 43 421 031 | 94,42 | 14019 |
| STE-U 9903 | 8 418 642 | 2 525 592 600 | 8 394 110 | 2 442 078 360 | 96,69 | 53,10 | 16 461 239 | 97,77 | 43 420 181 | 94,42 | 14040 |
| STE-U 9904 | 13 260 360 | 3 978 108 000 | 13 215 046 | 3 844 246 313 | 96,64 | 83,60 | 25 774 504 | 97,19 | 43 745 829 | 95,13 | 14033 |
| STE-U 9905 | 9 226 848 | 2 768 054 400 | 9 192 469 | 2 674 164 147 | 96,61 | 58,15 | 17 949 597 | 97,27 | 43 641 259 | 94,90 | 14055 |
| STE-U 9906 | 7 463 798 | 2 239 139 400 | 7 439 148 | 2 164 199 521 | 96,65 | 47,06 | 14 569 450 | 97,60 | 43 562 179 | 94,73 | 13997 |
| STE-U 9907 | 9 508 144 | 2 852 443 200 | 9 475 665 | 2 756 579 983 | 96,64 | 59,94 | 18 586 102 | 97,74 | 43 617 292 | 94,85 | 13999 |
| STE-U 9908 | 8 383 373 | 2 515 011 900 | 8 354 222 | 2 430 299 916 | 96,63 | 52,85 | 16 260 414 | 96,98 | 43 540 804 | 94,68 | 14089 |
| STE-U 9910 | 7 334 617 | 2 200 385 100 | 7 307 488 | 2 125 724 816 | 96,61 | 46,22 | 14 355 162 | 97,86 | 43 544 055 | 94,69 | 14024 |
| STE-U 9912 | 11 001 548 | 3 300 464 400 | 10 969 186 | 3 191 195 262 | 96,69 | 69,39 | 21 424 451 | 97,37 | 43 726 419 | 95,09 | 13961 |
| STE-U 9914 | 8 806 409 | 2 641 922 700 | 8 778 799 | 2 553 753 046 | 96,66 | 55,53 | 17 177 292 | 97,53 | 43 694 611 | 95,02 | 13989 |
| STE-U 9915 | 8 064 180 | 2 419 254 000 | 8 039 939 | 2 339 008 670 | 96,68 | 50,86 | 15 742 932 | 97,61 | 43 631 664 | 94,88 | 14053 |
| STE-U 9916 | 10 550 112 | 3 165 033 600 | 10 514 563 | 3 058 804 446 | 96,64 | 66,52 | 20 873 548 | 98,93 | 43 637 143 | 94,89 | 14007 |
| STE-U 9918 | 10 289 297 | 3 086 789 100 | 10 249 413 | 2 981 689 426 | 96,60 | 64,84 | 20 462 840 | 99,44 | 43 486 433 | 94,56 | 14012 |
| STE-U 9919 | 10 093 081 | 3 027 924 300 | 10 061 562 | 2 927 114 428 | 96,67 | 63,65 | 19 757 620 | 97,88 | 43 693 839 | 95,01 | 14106 |
| STE-U 9920 | 9 417 760 | 2 825 328 000 | 9 389 627 | 2 731 631 254 | 96,68 | 59,40 | 18 097 163 | 96,08 | 43 770 775 | 95,18 | 13996 |
| STE-U 9921 | 9 551 257 | 2 865 377 100 | 9 514 578 | 2 767 805 103 | 96,59 | 60,19 | 18 786 262 | 98,34 | 42 906 249 | 93,30 | 13963 |
| STE-U 9922 | 11 182 087 | 3 354 626 100 | 11 136 395 | 3 239 666 388 | 96,57 | 70,45 | 21 700 136 | 97,03 | 43 543 790 | 94,69 | 14047 |
| STE-U 9923 | 10 596 223 | 3 178 866 900 | 10 553 323 | 3 069 941 182 | 96,57 | 66,76 | 20 552 917 | 96,98 | 43 655 287 | 94,93 | 14045 |
| STE-U 9925 | 11 567 361 | 3 470 208 300 | 11 523 082 | 3 352 151 083 | 96,60 | 72,89 | 22 481 693 | 97,18 | 43 751 639 | 95,14 | 14014 |
| STE-U 9927 | 10 316 695 | 3 095 008 500 | 10 284 316 | 2 991 929 487 | 96,67 | 65,06 | 19 776 061 | 95,84 | 43 685 219 | 95,00 | 13945 |
| STE-U 9928 | 6 711 566 | 2 013 469 800 | 6 687 209 | 1 945 383 979 | 96,62 | 42,30 | 13 048 246 | 97,21 | 43 495 532 | 94,58 | 14001 |
| STE-U 9929 | 8 365 683 | 2 509 704 900 | 8 335 758 | 2 424 999 071 | 96,62 | 52,73 | 16 379 564 | 97,90 | 43 730 456 | 95,09 | 14040 |
| STE-U 9930 | 11 623 016 | 3 486 904 800 | 11 577 169 | 3 367 418 500 | 96,57 | 73,23 | 22 405 478 | 96,38 | 43 758 413 | 95,15 | 14043 |
