# Supplementary material for: Whole genome sequencing reveals the genetic diversity and structure of Leptosphaeria maculans populations from the Western Cape province of South Africa
Source: BMC Genomics. 2025 Apr 3;26:334. doi: 10.1186/s12864-025-11413-3 (PMC11966903; doi:10.1186/s12864-025-11413-3)
Supplement: Supplementary file 4 — Supplementary Material 4 [file 12864_2025_11413_MOESM4_ESM.docx]

**Table S4** Statistics for high throughput sequencing data of 205 *Leptosphaeria maculans* isolates downloaded from GenBank, before and after filtering

| **Isolate number** | **Sequence read run (SRR) number** |  | **Before filtering** | | |  | **After filtering** | | |  | **Total data (bp)** | **Theoretical coverage (%)** |
| --- | --- | --- | --- | --- | --- | --- | --- | --- | --- | --- | --- | --- |
|  |  |  | **Number of read pairs** | **Data in one read (bp)** | **Amount of data (bp)** |  | **Number of read pairs** | **Data in forward read (bp)** | **Data in reverse read (bp)** |  |  |  |
| IBCN094 | SRR24874327 |  | 10529835 | 1590005085 | 3180010170 |  | 10487335 | 1524771109 | 1525304210 |  | 3050075319 | 66,32548 |
| IBCN095 | SRR24874326 |  | 8227855 | 1242406105 | 2484812210 |  | 8195497 | 1191492174 | 1192005625 |  | 2383497799 | 51,83041 |
| IBCN096 | SRR24874215 |  | 6372159 | 962196009 | 1924392018 |  | 6354000 | 924087103 | 923750582 |  | 1847837685 | 40,1822 |
| IBCN097 | SRR24874129 |  | 6318059 | 954026909 | 1908053818 |  | 6299776 | 915761018 | 916144326 |  | 1831905344 | 39,83574 |
| IBCN098 | SRR24874174 |  | 5300696 | 800405096 | 1600810192 |  | 5286155 | 768666479 | 768411588 |  | 1537078067 | 33,42457 |
| IBCN099 | SRR24874163 |  | 5179069 | 782039419 | 1564078838 |  | 5168118 | 751480904 | 751464234 |  | 1502945138 | 32,68233 |
| IBCN100 | SRR24874152 |  | 5128485 | 774401235 | 1548802470 |  | 5112289 | 742723652 | 743483367 |  | 1486207019 | 32,31835 |
| IBCN101 | SRR24874291 |  | 5352578 | 808239278 | 1616478556 |  | 5329474 | 774827570 | 774934370 |  | 1549761940 | 33,70038 |
| IBCN102 | SRR24874280 |  | 5173234 | 781158334 | 1562316668 |  | 5160905 | 750469708 | 750422516 |  | 1500892224 | 32,63769 |
| IBCN103 | SRR24874269 |  | 6040730 | 912150230 | 1824300460 |  | 6021479 | 875664198 | 875444403 |  | 1751108601 | 38,07877 |
| IBCN104 | SRR24874325 |  | 6282831 | 948707481 | 1897414962 |  | 6263106 | 910669491 | 910614542 |  | 1821284033 | 39,60477 |
| IBCN105 | SRR24874258 |  | 6104090 | 921717590 | 1843435180 |  | 6084250 | 884519413 | 884753186 |  | 1769272599 | 38,47376 |
| IBCN106 | SRR24874247 |  | 10367694 | 1565521794 | 3131043588 |  | 10328901 | 1501861884 | 1502360122 |  | 3004222006 | 65,32838 |
| IBCN107 | SRR24874198 |  | 7691194 | 1161370294 | 2322740588 |  | 7661786 | 1114032842 | 1114288724 |  | 2228321566 | 48,45602 |
| IBCN108 | SRR24874187 |  | 7938893 | 1198772843 | 2397545686 |  | 7910086 | 1150142575 | 1150412879 |  | 2300555454 | 50,02678 |
| IBCN109 | SRR24874176 |  | 10062487 | 1519435537 | 3038871074 |  | 10025132 | 1457657463 | 1457989424 |  | 2915646887 | 63,40227 |
| IBCN110 | SRR24874315 |  | 8771449 | 1324488799 | 2648977598 |  | 8738374 | 1270091237 | 1270679313 |  | 2540770550 | 55,25038 |
| IBCN111 | SRR24874304 |  | 8634693 | 1303838643 | 2607677286 |  | 8601859 | 1250452831 | 1250991896 |  | 2501444727 | 54,39522 |
| IBCN112 | SRR24874237 |  | 7894053 | 1192002003 | 2384004006 |  | 7862436 | 1142975621 | 1143639422 |  | 2286615043 | 49,72364 |
| IBCN113 | SRR24874226 |  | 8646328 | 1305595528 | 2611191056 |  | 8616793 | 1252701985 | 1253325811 |  | 2506027796 | 54,49489 |
| IBCN114 | SRR24874214 |  | 10211508 | 1541937708 | 3083875416 |  | 10170328 | 1478606327 | 1479198733 |  | 2957805060 | 64,31902 |
| IBCN115 | SRR24874138 |  | 6602364 | 996956964 | 1993913928 |  | 6577627 | 956381304 | 956422442 |  | 1912803746 | 41,59492 |
| IBCN116 | SRR24874137 |  | 6214322 | 938362622 | 1876725244 |  | 6191356 | 899614730 | 900273356 |  | 1799888086 | 39,13951 |
| IBCN118 | SRR24874136 |  | 5219610 | 788161110 | 1576322220 |  | 5197641 | 755592554 | 755764380 |  | 1511356934 | 32,86525 |
| IBCN119 | SRR24874135 |  | 6071711 | 916828361 | 1833656722 |  | 6047052 | 878450704 | 879359264 |  | 1757809968 | 38,2245 |
| IBCN120 | SRR24874134 |  | 6261534 | 945491634 | 1890983268 |  | 6233332 | 906008041 | 906349863 |  | 1812357904 | 39,41067 |
| IBCN125 | SRR24874132 |  | 7498044 | 1132204644 | 2264409288 |  | 7468632 | 1085748212 | 1086235365 |  | 2171983577 | 47,23092 |
| IBCN127 | SRR24874131 |  | 4729683 | 714182133 | 1428364266 |  | 4716107 | 685143336 | 685641897 |  | 1370785233 | 29,80844 |
| IBCN128 | SRR24874130 |  | 4086975 | 617133225 | 1234266450 |  | 4072075 | 592086668 | 592160029 |  | 1184246697 | 25,75206 |
| IBCN129 | SRR24874128 |  | 5456056 | 823864456 | 1647728912 |  | 5441562 | 791083007 | 791181575 |  | 1582264582 | 34,40717 |
| IBCN130 | SRR24874127 |  | 4337814 | 655009914 | 1310019828 |  | 4327036 | 629107999 | 629210319 |  | 1258318318 | 27,36279 |
| IBCN131 | SRR24874126 |  | 5226494 | 789200594 | 1578401188 |  | 5210838 | 757733564 | 757520704 |  | 1515254268 | 32,95 |
| IBCN133 | SRR24874125 |  | 8253195 | 1246232445 | 2492464890 |  | 8224175 | 1195821338 | 1196103822 |  | 2391925160 | 52,01366 |
| IBCN135 | SRR24874124 |  | 8424666 | 1272124566 | 2544249132 |  | 8391674 | 1219817622 | 1220416659 |  | 2440234281 | 53,06417 |
| IBCN136 | SRR24874123 |  | 10419027 | 1573273077 | 3146546154 |  | 10378359 | 1508966989 | 1509438365 |  | 3018405354 | 65,6368 |
| IBCN137 | SRR24874122 |  | 8484453 | 1281152403 | 2562304806 |  | 8451796 | 1228826855 | 1229240070 |  | 2458066925 | 53,45195 |
| IBCN138 | SRR24874121 |  | 7508754 | 1133821854 | 2267643708 |  | 7480226 | 1087455289 | 1087931445 |  | 2175386734 | 47,30492 |
| IBCN139 | SRR24874120 |  | 9903707 | 1495459757 | 2990919514 |  | 9866773 | 1434588728 | 1434993478 |  | 2869582206 | 62,40057 |
| IBCN140 | SRR24874119 |  | 8613419 | 1300626269 | 2601252538 |  | 8579978 | 1247335954 | 1247976720 |  | 2495312674 | 54,26188 |
| IBCN141 | SRR24874173 |  | 7790792 | 1176409592 | 2352819184 |  | 7760421 | 1127653566 | 1128403563 |  | 2256057129 | 49,05914 |
| IBCN142 | SRR24874172 |  | 8906430 | 1344870930 | 2689741860 |  | 8871296 | 1289714624 | 1290314560 |  | 2580029184 | 56,10408 |
| IBCN143 | SRR24874171 |  | 6311672 | 953062472 | 1906124944 |  | 6284395 | 913598331 | 913837439 |  | 1827435770 | 39,73855 |
| IBCN144 | SRR24874170 |  | 6444153 | 973067103 | 1946134206 |  | 6415361 | 932714892 | 932778194 |  | 1865493086 | 40,56612 |
| IBCN145 | SRR24874169 |  | 5487841 | 828663991 | 1657327982 |  | 5464643 | 794457456 | 794696246 |  | 1589153702 | 34,55698 |
| IBCN146 | SRR24874168 |  | 5072607 | 765963657 | 1531927314 |  | 5050092 | 734072388 | 734299637 |  | 1468372025 | 31,93052 |
| IBCN147 | SRR24874167 |  | 5071380 | 765778380 | 1531556760 |  | 5050120 | 734278431 | 734249432 |  | 1468527863 | 31,93391 |
| IBCN148 | SRR24874166 |  | 10645393 | 1607454343 | 3214908686 |  | 10602581 | 1541567330 | 1541719201 |  | 3083286531 | 67,04768 |
| IBCN149 | SRR24874165 |  | 8370493 | 1263944443 | 2527888886 |  | 8339182 | 1212211469 | 1213026097 |  | 2425237566 | 52,73806 |
| IBCN150 | SRR24874164 |  | 8921022 | 1347074322 | 2694148644 |  | 8888666 | 1292131700 | 1293006454 |  | 2585138154 | 56,21518 |
| IBCN151 | SRR24874162 |  | 5777465 | 872397215 | 1744794430 |  | 5755469 | 836246059 | 836958075 |  | 1673204134 | 36,3847 |
| IBCN152 | SRR24874161 |  | 7961260 | 1202150260 | 2404300520 |  | 7930590 | 1152955947 | 1153612830 |  | 2306568777 | 50,15754 |
| IBCN153 | SRR24874160 |  | 6332195 | 956161445 | 1912322890 |  | 6307398 | 916513129 | 917165155 |  | 1833678284 | 39,87429 |
| IBCN154 | SRR24874159 |  | 7010503 | 1058585953 | 2117171906 |  | 6980638 | 1014910902 | 1015005656 |  | 2029916558 | 44,1416 |
| IBCN155 | SRR24874158 |  | 5406057 | 816314607 | 1632629214 |  | 5384468 | 782620287 | 782998687 |  | 1565618974 | 34,0452 |
| IBCN156 | SRR24874157 |  | 4629048 | 698986248 | 1397972496 |  | 4609304 | 670118228 | 670177595 |  | 1340295823 | 29,14543 |
| IBCN157 | SRR24874156 |  | 5716139 | 863136989 | 1726273978 |  | 5691797 | 827491687 | 827566544 |  | 1655058231 | 35,99011 |
| IBCN158 | SRR24874155 |  | 6287809 | 949459159 | 1898918318 |  | 6260814 | 910292087 | 910391087 |  | 1820683174 | 39,59171 |
| IBCN159 | SRR24874154 |  | 6569405 | 991980155 | 1983960310 |  | 6543826 | 951451446 | 951435177 |  | 1902886623 | 41,37927 |
| IBCN160 | SRR24874153 |  | 6232901 | 941168051 | 1882336102 |  | 6206154 | 902220507 | 902356536 |  | 1804577043 | 39,24147 |
| IBCN161 | SRR24874151 |  | 5400951 | 815543601 | 1631087202 |  | 5377139 | 781663629 | 781741462 |  | 1563405091 | 33,99706 |
| IBCN162 | SRR24874150 |  | 5429892 | 819913692 | 1639827384 |  | 5407188 | 786017951 | 786242248 |  | 1572260199 | 34,18962 |
| IBCN163 | SRR24874149 |  | 5663571 | 855199221 | 1710398442 |  | 5638901 | 819690429 | 819719838 |  | 1639410267 | 35,64983 |
| IBCN164 | SRR24874148 |  | 5499880 | 830481880 | 1660963760 |  | 5477679 | 796425837 | 796515551 |  | 1592941388 | 34,63934 |
| IBCN165 | SRR24874147 |  | 6016132 | 908435932 | 1816871864 |  | 5989376 | 870672996 | 870856062 |  | 1741529058 | 37,87046 |
| IBCN166 | SRR24874296 |  | 10398426 | 1570162326 | 3140324652 |  | 10357827 | 1505945971 | 1506458365 |  | 3012404336 | 65,50631 |
| IBCN167 | SRR24874295 |  | 5746958 | 867790658 | 1735581316 |  | 5730276 | 833237181 | 832924376 |  | 1666161557 | 36,23155 |
| IBCN168 | SRR24874294 |  | 5592611 | 844484261 | 1688968522 |  | 5572365 | 810270534 | 810233154 |  | 1620503688 | 35,2387 |
| IBCN169 | SRR24874293 |  | 4951772 | 747717572 | 1495435144 |  | 4931324 | 716648633 | 717148534 |  | 1433797167 | 31,17867 |
| IBCN170 | SRR24874292 |  | 5095923 | 769484373 | 1538968746 |  | 5078632 | 737253614 | 738205549 |  | 1475459163 | 32,08463 |
| IBCN172 | SRR24874290 |  | 4922100 | 743237100 | 1486474200 |  | 4900830 | 712472779 | 712403176 |  | 1424875955 | 30,98467 |
| IBCN173 | SRR24874289 |  | 5010778 | 756627478 | 1513254956 |  | 4989884 | 725328438 | 725456813 |  | 1450785251 | 31,54808 |
| IBCN174 | SRR24874288 |  | 5198443 | 784964893 | 1569929786 |  | 5174576 | 752229451 | 752340976 |  | 1504570427 | 32,71767 |
| IBCN175 | SRR24874287 |  | 14260512 | 2153337312 | 4306674624 |  | 14212419 | 2068751666 | 2066328998 |  | 4135080664 | 89,91949 |
| IBCN176 | SRR24874286 |  | 9723648 | 1468270848 | 2936541696 |  | 9678003 | 1406851602 | 1407165289 |  | 2814016891 | 61,19227 |
| IBCN177 | SRR24874285 |  | 5769172 | 871144972 | 1742289944 |  | 5741602 | 834721457 | 834665286 |  | 1669386743 | 36,30169 |
| IBCN178 | SRR24874284 |  | 5922349 | 894274699 | 1788549398 |  | 5895591 | 856895235 | 857227923 |  | 1714123158 | 37,2745 |
| IBCN179 | SRR24874283 |  | 5437683 | 821090133 | 1642180266 |  | 5413669 | 787053452 | 787199115 |  | 1574252567 | 34,23295 |
| IBCN180 | SRR24874282 |  | 6664983 | 1006412433 | 2012824866 |  | 6634465 | 964405226 | 964668257 |  | 1929073483 | 41,94871 |
| IBCN181 | SRR24874281 |  | 5064681 | 764766831 | 1529533662 |  | 5042994 | 732889198 | 733193790 |  | 1466082988 | 31,88074 |
| IBCN182 | SRR24874279 |  | 7600337 | 1147650887 | 2295301774 |  | 7570608 | 1100796796 | 1100826767 |  | 2201623563 | 47,87546 |
| IBCN183 | SRR24874278 |  | 5016816 | 757539216 | 1515078432 |  | 4994783 | 725539341 | 726334074 |  | 1451873415 | 31,57175 |
| IBCN184 | SRR24874277 |  | 7782031 | 1175086681 | 2350173362 |  | 7748984 | 1126582780 | 1126657325 |  | 2253240105 | 48,99788 |
| IBCN185 | SRR24874276 |  | 5427258 | 819515958 | 1639031916 |  | 5402924 | 785494395 | 785639497 |  | 1571133892 | 34,16513 |
| IBCN187 | SRR24874274 |  | 5703040 | 861159040 | 1722318080 |  | 5678566 | 825614560 | 825559979 |  | 1651174539 | 35,90565 |
| IBCN188 | SRR24874273 |  | 6016259 | 908455109 | 1816910218 |  | 5989083 | 870389702 | 870723439 |  | 1741113141 | 37,86142 |
| IBCN189 | SRR24874272 |  | 5165215 | 779947465 | 1559894930 |  | 5145675 | 748134358 | 748252586 |  | 1496386944 | 32,53972 |
| IBCN190 | SRR24874271 |  | 6325431 | 955140081 | 1910280162 |  | 6297744 | 915592192 | 915666221 |  | 1831258413 | 39,82167 |
| IBCN191 | SRR24874270 |  | 5908640 | 892204640 | 1784409280 |  | 5886210 | 855783576 | 855976001 |  | 1711759577 | 37,22311 |
| IBCN193 | SRR24874211 |  | 5700127 | 860719177 | 1721438354 |  | 5675567 | 825120660 | 825164025 |  | 1650284685 | 35,8863 |
| IBCN194 | SRR24874210 |  | 6550112 | 989066912 | 1978133824 |  | 6526550 | 949032100 | 948924608 |  | 1897956708 | 41,27206 |
| IBCN195 | SRR24874209 |  | 5175555 | 781508805 | 1563017610 |  | 5158429 | 749881460 | 750174160 |  | 1500055620 | 32,61949 |
| IBCN196 | SRR24874208 |  | 4182842 | 631609142 | 1263218284 |  | 4167456 | 605853647 | 606094887 |  | 1211948534 | 26,35445 |
| IBCN197 | SRR24874207 |  | 6968769 | 1052284119 | 2104568238 |  | 6938478 | 1008696547 | 1008626230 |  | 2017322777 | 43,86774 |
| IBCN198 | SRR24874206 |  | 6546040 | 988452040 | 1976904080 |  | 6519856 | 947631281 | 948094153 |  | 1895725434 | 41,22354 |
| IBCN199 | SRR24874205 |  | 7994836 | 1207220236 | 2414440472 |  | 7963275 | 1157203973 | 1158016638 |  | 2315220611 | 50,34568 |
| IBCN200 | SRR24874204 |  | 8356944 | 1261898544 | 2523797088 |  | 8321654 | 1209903805 | 1209608767 |  | 2419512572 | 52,61357 |
| IBCN201 | SRR24874202 |  | 5525730 | 834385230 | 1668770460 |  | 5504458 | 799921853 | 800426425 |  | 1600348278 | 34,80041 |
| IBCN202 | SRR24874268 |  | 3931990 | 593730490 | 1187460980 |  | 3918920 | 569802578 | 569842362 |  | 1139644940 | 24,78218 |
| IBCN203 | SRR24874267 |  | 6077233 | 917662183 | 1835324366 |  | 6052538 | 879925525 | 879932638 |  | 1759858163 | 38,26904 |
| IBCN204 | SRR24874266 |  | 6595918 | 995983618 | 1991967236 |  | 6568225 | 954995780 | 955061130 |  | 1910056910 | 41,53519 |
| IBCN205 | SRR24874265 |  | 4810123 | 726328573 | 1452657146 |  | 4790261 | 696519743 | 696663594 |  | 1393183337 | 30,2955 |
| IBCN206 | SRR24874264 |  | 5506233 | 831441183 | 1662882366 |  | 5484375 | 797288880 | 797491546 |  | 1594780426 | 34,67933 |
| IBCN207 | SRR24874263 |  | 6039821 | 912012971 | 1824025942 |  | 6016210 | 874707820 | 874797503 |  | 1749505323 | 38,04391 |
| IBCN208 | SRR24874262 |  | 8191964 | 1236986564 | 2473973128 |  | 8160031 | 1186430753 | 1186670013 |  | 2373100766 | 51,60432 |
| IBCN209 | SRR24874261 |  | 5533896 | 835618296 | 1671236592 |  | 5511883 | 801092415 | 801460125 |  | 1602552540 | 34,84834 |
| IBCN210 | SRR24874260 |  | 5181234 | 782366334 | 1564732668 |  | 5160138 | 750112176 | 750326180 |  | 1500438356 | 32,62782 |
| IBCN211 | SRR24874259 |  | 5141753 | 776404703 | 1552809406 |  | 5121436 | 744574823 | 744808776 |  | 1489383599 | 32,38743 |
| IBCN212 | SRR24874257 |  | 6825525 | 1030654275 | 2061308550 |  | 6799262 | 988239001 | 988753650 |  | 1976992651 | 42,99074 |
| IBCN213 | SRR24874256 |  | 6385924 | 964274524 | 1928549048 |  | 6358654 | 924463898 | 924595759 |  | 1849059657 | 40,20877 |
| IBCN214 | SRR24874255 |  | 5324557 | 804008107 | 1608016214 |  | 5304361 | 771285614 | 771422179 |  | 1542707793 | 33,54699 |
| IBCN215 | SRR24874254 |  | 6170858 | 931799558 | 1863599116 |  | 6146293 | 893514907 | 893676914 |  | 1787191821 | 38,86342 |
| IBCN216 | SRR24874253 |  | 3648280 | 550890280 | 1101780560 |  | 3634055 | 528242457 | 528431116 |  | 1056673573 | 22,97792 |
| IBCN217 | SRR24874252 |  | 5872347 | 886724397 | 1773448794 |  | 5847789 | 850057169 | 850387669 |  | 1700444838 | 36,97706 |
| IBCN218 | SRR24874251 |  | 5082640 | 767478640 | 1534957280 |  | 5060872 | 735718418 | 735876096 |  | 1471594514 | 32,00059 |
| IBCN224 | SRR24874250 |  | 9102777 | 1374519327 | 2749038654 |  | 9065202 | 1317853862 | 1318512757 |  | 2636366619 | 57,32917 |
| IBCN237 | SRR24874248 |  | 9932590 | 1499821090 | 2999642180 |  | 9892965 | 1438281728 | 1438942489 |  | 2877224217 | 62,56675 |
| IBCN238 | SRR24874246 |  | 12319771 | 1860285421 | 3720570842 |  | 12268490 | 1783734333 | 1784083927 |  | 3567818260 | 77,58407 |
| IBCN239 | SRR24874245 |  | 11459436 | 1730374836 | 3460749672 |  | 11412954 | 1659178151 | 1659923523 |  | 3319101674 | 72,1756 |
| IBCN240 | SRR24874244 |  | 9914043 | 1497020493 | 2994040986 |  | 9875107 | 1435308786 | 1436149123 |  | 2871457909 | 62,44135 |
| IBCN241 | SRR24874243 |  | 9803245 | 1480289995 | 2960579990 |  | 9766208 | 1419374309 | 1420685414 |  | 2840059723 | 61,75858 |
| IBCN242 | SRR24874242 |  | 11385178 | 1719161878 | 3438323756 |  | 11343356 | 1649206725 | 1649858701 |  | 3299065426 | 71,7399 |
| IBCN243 | SRR24874241 |  | 7922902 | 1196358202 | 2392716404 |  | 7893222 | 1147671432 | 1148066006 |  | 2295737438 | 49,92201 |
| IBCN244 | SRR24874203 |  | 7758337 | 1171508887 | 2343017774 |  | 7728189 | 1123559844 | 1123926295 |  | 2247486139 | 48,87276 |
| IBCN245 | SRR24874201 |  | 8990399 | 1357550249 | 2715100498 |  | 8959074 | 1302593824 | 1303192233 |  | 2605786057 | 56,66418 |
| IBCN246 | SRR24874200 |  | 8264878 | 1247996578 | 2495993156 |  | 8237280 | 1197706983 | 1198161646 |  | 2395868629 | 52,09942 |
| IBCN247 | SRR24874199 |  | 9948454 | 1502216554 | 3004433108 |  | 9912159 | 1440762767 | 1441937346 |  | 2882700113 | 62,68582 |
| IBCN249 | SRR24874197 |  | 7922250 | 1196259750 | 2392519500 |  | 7892091 | 1147316394 | 1147751337 |  | 2295067731 | 49,90745 |
| IBCN250 | SRR24874196 |  | 9678551 | 1461461201 | 2922922402 |  | 9641376 | 1401234579 | 1402342720 |  | 2803577299 | 60,96526 |
| IBCN251 | SRR24874195 |  | 10985816 | 1658858216 | 3317716432 |  | 10940807 | 1590702800 | 1589005844 |  | 3179708644 | 69,14443 |
| IBCN252 | SRR24874194 |  | 10195789 | 1539564139 | 3079128278 |  | 10159848 | 1477087428 | 1477799836 |  | 2954887264 | 64,25557 |
| IBCN253 | SRR24874193 |  | 9892856 | 1493821256 | 2987642512 |  | 9855177 | 1432851954 | 1433444189 |  | 2866296143 | 62,32911 |
| IBCN254 | SRR24874192 |  | 10516585 | 1588004335 | 3176008670 |  | 10477051 | 1522687324 | 1523702293 |  | 3046389617 | 66,24534 |
| IBCN255 | SRR24874191 |  | 11461340 | 1730662340 | 3461324680 |  | 11416053 | 1659774673 | 1660274065 |  | 3320048738 | 72,1962 |
| IBCN256 | SRR24874190 |  | 9417510 | 1422044010 | 2844088020 |  | 9381199 | 1363965809 | 1364506596 |  | 2728472405 | 59,33206 |
| IBCN257 | SRR24874189 |  | 9308113 | 1405525063 | 2811050126 |  | 9273004 | 1348207966 | 1348690526 |  | 2696898492 | 58,64547 |
| IBCN258 | SRR24874188 |  | 8017314 | 1210614414 | 2421228828 |  | 7985775 | 1161007470 | 1161604842 |  | 2322612312 | 50,50642 |
| IBCN259 | SRR24874186 |  | 8397845 | 1268074595 | 2536149190 |  | 8364580 | 1216094920 | 1216505205 |  | 2432600125 | 52,89816 |
| IBCN260 | SRR24874185 |  | 7247592 | 1094386392 | 2188772784 |  | 7221797 | 1049827420 | 1050564155 |  | 2100391575 | 45,67411 |
| IBCN261 | SRR24874184 |  | 10839220 | 1636722220 | 3273444440 |  | 10797323 | 1569901510 | 1570388512 |  | 3140290022 | 68,28725 |
| IBCN262 | SRR24874183 |  | 11152623 | 1684046073 | 3368092146 |  | 11112887 | 1615140599 | 1616436962 |  | 3231577561 | 70,27234 |
| IBCN263 | SRR24874182 |  | 9381055 | 1416539305 | 2833078610 |  | 9345075 | 1358464602 | 1359204667 |  | 2717669269 | 59,09714 |
| IBCN264 | SRR24874181 |  | 8401532 | 1268631332 | 2537262664 |  | 8369200 | 1216769914 | 1217333488 |  | 2434103402 | 52,93085 |
| IBCN265 | SRR24874180 |  | 10591532 | 1599321332 | 3198642664 |  | 10551873 | 1534092396 | 1534735575 |  | 3068827971 | 66,73327 |
| IBCN266 | SRR24874179 |  | 9817688 | 1482470888 | 2964941776 |  | 9779855 | 1421694898 | 1422416754 |  | 2844111652 | 61,8467 |
| IBCN267 | SRR24874178 |  | 9176607 | 1385667657 | 2771335314 |  | 9141767 | 1328994381 | 1329511355 |  | 2658505736 | 57,8106 |
| IBCN268 | SRR24874177 |  | 8247400 | 1245357400 | 2490714800 |  | 8216113 | 1194560066 | 1195004601 |  | 2389564667 | 51,96233 |
| IBCN269 | SRR24874175 |  | 8061560 | 1217295560 | 2434591120 |  | 8030178 | 1167333714 | 1168142670 |  | 2335476384 | 50,78616 |
| IBCN270 | SRR24874324 |  | 9429545 | 1423861295 | 2847722590 |  | 9394730 | 1365384172 | 1366410060 |  | 2731794232 | 59,40429 |
| IBCN271 | SRR24874323 |  | 8130544 | 1227712144 | 2455424288 |  | 8100613 | 1177605307 | 1178338063 |  | 2355943370 | 51,23122 |
| IBCN272 | SRR24874322 |  | 8902890 | 1344336390 | 2688672780 |  | 8870441 | 1289633323 | 1290228024 |  | 2579861347 | 56,10043 |
| IBCN273 | SRR24874321 |  | 9153335 | 1382153585 | 2764307170 |  | 9119944 | 1325319542 | 1326677948 |  | 2651997490 | 57,66907 |
| IBCN274 | SRR24874320 |  | 11480362 | 1733534662 | 3467069324 |  | 11436367 | 1662689358 | 1663266465 |  | 3325955823 | 72,32465 |
| IBCN275 | SRR24874319 |  | 9181995 | 1386481245 | 2772962490 |  | 9147778 | 1330090930 | 1330621879 |  | 2660712809 | 57,85859 |
| IBCN276 | SRR24874318 |  | 8457796 | 1277127196 | 2554254392 |  | 8427282 | 1225154397 | 1225699134 |  | 2450853531 | 53,29509 |
| IBCN277 | SRR24874317 |  | 11783432 | 1779298232 | 3558596464 |  | 11738360 | 1706586165 | 1707278685 |  | 3413864850 | 74,23628 |
| IBCN278 | SRR24874316 |  | 11174895 | 1687409145 | 3374818290 |  | 11133882 | 1618804557 | 1619251219 |  | 3238055776 | 70,41322 |
| IBCN279 | SRR24874314 |  | 10562641 | 1594958791 | 3189917582 |  | 10522435 | 1529637034 | 1530743307 |  | 3060380341 | 66,54957 |
| IBCN280 | SRR24874313 |  | 6295359 | 950599209 | 1901198418 |  | 6273795 | 912173617 | 912567358 |  | 1824740975 | 39,67995 |
| IBCN281 | SRR24874312 |  | 8035975 | 1213432225 | 2426864450 |  | 8005182 | 1163731509 | 1164280314 |  | 2328011823 | 50,62383 |
| IBCN282 | SRR24874311 |  | 9425845 | 1423302595 | 2846605190 |  | 9389079 | 1365135104 | 1365633959 |  | 2730769063 | 59,382 |
| IBCN283 | SRR24874310 |  | 6606827 | 997630877 | 1995261754 |  | 6581690 | 956850367 | 957292070 |  | 1914142437 | 41,62403 |
| IBCN284 | SRR24874309 |  | 10709074 | 1617070174 | 3234140348 |  | 10669678 | 1551371437 | 1551827330 |  | 3103198767 | 67,48068 |
| IBCN285 | SRR24874308 |  | 8468319 | 1278716169 | 2557432338 |  | 8434964 | 1226147261 | 1226894579 |  | 2453041840 | 53,34268 |
| IBCN286 | SRR24874307 |  | 8488470 | 1281758970 | 2563517940 |  | 8455521 | 1229290738 | 1229813883 |  | 2459104621 | 53,47452 |
| IBCN288 | SRR24874306 |  | 10676918 | 1612214618 | 3224429236 |  | 10637018 | 1546533411 | 1547029389 |  | 3093562800 | 67,27114 |
| IBCN289 | SRR24874305 |  | 10475717 | 1581833267 | 3163666534 |  | 10440006 | 1517882836 | 1518247581 |  | 3036130417 | 66,02224 |
| IBCN290 | SRR24874303 |  | 8315105 | 1255580855 | 2511161710 |  | 8283281 | 1204187998 | 1204943520 |  | 2409131518 | 52,38783 |
| IBCN291 | SRR24874302 |  | 9442858 | 1425871558 | 2851743116 |  | 9408079 | 1367233075 | 1368540923 |  | 2735773998 | 59,49084 |
| IBCN292 | SRR24874301 |  | 11592124 | 1750410724 | 3500821448 |  | 11545225 | 1678577211 | 1679165563 |  | 3357742774 | 73,01587 |
| IBCN293 | SRR24874300 |  | 6616261 | 999055411 | 1998110822 |  | 6591309 | 958081930 | 958832639 |  | 1916914569 | 41,68431 |
| IBCN294 | SRR24874299 |  | 8084970 | 1220830470 | 2441660940 |  | 8053727 | 1170763774 | 1171375304 |  | 2342139078 | 50,93104 |
| IBCN295 | SRR24874298 |  | 9637278 | 1455228978 | 2910457956 |  | 9602877 | 1396187476 | 1396794666 |  | 2792982142 | 60,73486 |
| IBCN297 | SRR24874297 |  | 9788412 | 1478050212 | 2956100424 |  | 9750074 | 1417592931 | 1418095527 |  | 2835688458 | 61,66353 |
| IBCN299 | SRR24874240 |  | 8449979 | 1275946829 | 2551893658 |  | 8417570 | 1223718275 | 1224410103 |  | 2448128378 | 53,23583 |
| IBCN300 | SRR24874239 |  | 8839916 | 1334827316 | 2669654632 |  | 8806192 | 1280161717 | 1280899786 |  | 2561061503 | 55,69162 |
| IBCN301 | SRR24874238 |  | 11271164 | 1701945764 | 3403891528 |  | 11230049 | 1632708350 | 1633524948 |  | 3266233298 | 71,02595 |
| IBCN302 | SRR24874236 |  | 10267763 | 1550432213 | 3100864426 |  | 10228133 | 1486598600 | 1487680425 |  | 2974279025 | 64,67725 |
| IBCN303 | SRR24874235 |  | 8747734 | 1320907834 | 2641815668 |  | 8714761 | 1267020828 | 1267491873 |  | 2534512701 | 55,1143 |
| IBCN304 | SRR24874234 |  | 10378933 | 1567218883 | 3134437766 |  | 10338778 | 1503144617 | 1503792164 |  | 3006936781 | 65,38741 |
| IBCN305 | SRR24874233 |  | 8766301 | 1323711451 | 2647422902 |  | 8732862 | 1269671796 | 1270175513 |  | 2539847309 | 55,23031 |
| IBCN306 | SRR24874232 |  | 15730817 | 2375353367 | 4750706734 |  | 15687929 | 2281163336 | 2282671405 |  | 4563834741 | 99,24297 |
| IBCN307 | SRR24874231 |  | 15249257 | 2302637807 | 4605275614 |  | 15213176 | 2211927150 | 2213870764 |  | 4425797914 | 96,24129 |
| IBCN308 | SRR24874230 |  | 16051165 | 2423725915 | 4847451830 |  | 16001592 | 2326618897 | 2328118763 |  | 4654737660 | 101,2197 |
| IBCN309 | SRR24874229 |  | 16391613 | 2475133563 | 4950267126 |  | 16340847 | 2375984067 | 2377548759 |  | 4753532826 | 103,3681 |
| IBCN310 | SRR24874228 |  | 13387413 | 2021499363 | 4042998726 |  | 13336098 | 1938943691 | 1940118737 |  | 3879062428 | 84,35224 |
| IBCN311 | SRR24874227 |  | 17572742 | 2653484042 | 5306968084 |  | 17520402 | 2547860991 | 2548975411 |  | 5096836402 | 110,8334 |
| IBCN312 | SRR24874225 |  | 16242052 | 2452549852 | 4905099704 |  | 16185787 | 2353469493 | 2354790633 |  | 4708260126 | 102,3836 |
| IBCN313 | SRR24874224 |  | 16608341 | 2507859491 | 5015718982 |  | 16552378 | 2406727359 | 2408171030 |  | 4814898389 | 104,7025 |
| IBCN314 | SRR24874223 |  | 16499150 | 2491371650 | 4982743300 |  | 16442438 | 2390871860 | 2391978053 |  | 4782849913 | 104,0056 |
| IBCN315 | SRR24874222 |  | 18176836 | 2744702236 | 5489404472 |  | 18121946 | 2634810691 | 2636611791 |  | 5271422482 | 114,6298 |
| IBCN316 | SRR24874221 |  | 18649228 | 2816033428 | 5632066856 |  | 18602124 | 2704717008 | 2707128922 |  | 5411845930 | 117,6834 |
| IBCN317 | SRR24874220 |  | 18206738 | 2749217438 | 5498434876 |  | 18153375 | 2639121886 | 2641474077 |  | 5280595963 | 114,8293 |
| IBCN318 | SRR24874219 |  | 16214429 | 2448378779 | 4896757558 |  | 16151080 | 2348645518 | 2349412503 |  | 4698058021 | 102,1617 |
| IBCN319 | SRR24874218 |  | 17423316 | 2630920716 | 5261841432 |  | 17362698 | 2524377940 | 2525964837 |  | 5050342777 | 109,8223 |
| IBCN320 | SRR24874217 |  | 15454820 | 2333677820 | 4667355640 |  | 15402063 | 2239438744 | 2240885558 |  | 4480324302 | 97,427 |
| IBCN321 | SRR24874216 |  | 19858120 | 2998576120 | 5997152240 |  | 19762913 | 2874281329 | 2874190957 |  | 5748472286 | 125,0035 |
| IBCN322 | SRR24874213 |  | 15821209 | 2389002559 | 4778005118 |  | 15769093 | 2292793056 | 2294148000 |  | 4586941056 | 99,74543 |
| IBCN323 | SRR24874146 |  | 15147447 | 2287264497 | 4574528994 |  | 15102775 | 2195803921 | 2197484724 |  | 4393288645 | 95,53436 |
| IBCN324 | SRR24874145 |  | 17444607 | 2634135657 | 5268271314 |  | 17386314 | 2527938840 | 2529448272 |  | 5057387112 | 109,9755 |
| IBCN325 | SRR24874144 |  | 17761278 | 2681952978 | 5363905956 |  | 17701616 | 2574031281 | 2575242611 |  | 5149273892 | 111,9737 |
| IBCN326 | SRR24874143 |  | 16143341 | 2437644491 | 4875288982 |  | 16094653 | 2340351253 | 2341467885 |  | 4681819138 | 101,8086 |
| IBCN327 | SRR24874142 |  | 17131810 | 2586903310 | 5173806620 |  | 17071794 | 2482426785 | 2483492248 |  | 4965919033 | 107,9865 |
| IBCN328 | SRR24874141 |  | 18228996 | 2752578396 | 5505156792 |  | 18174951 | 2643095975 | 2644311271 |  | 5287407246 | 114,9774 |
| IBCN329 | SRR24874140 |  | 16124888 | 2434858088 | 4869716176 |  | 16070657 | 2336650645 | 2338170670 |  | 4674821315 | 101,6564 |
| IBCN330 | SRR24874139 |  | 18064506 | 2727740406 | 5455480812 |  | 18004791 | 2617823929 | 2619642704 |  | 5237466633 | 113,8915 |
