# Supplementary material for: Whole genome sequencing reveals the genetic diversity and structure of Leptosphaeria maculans populations from the Western Cape province of South Africa
Source: BMC Genomics. 2025 Apr 3;26:334. doi: 10.1186/s12864-025-11413-3 (PMC11966903; doi:10.1186/s12864-025-11413-3)
Supplement: Supplementary file 5 — Supplementary Material 5 [file 12864_2025_11413_MOESM5_ESM.docx]

**Table S5** Information on mating type, and clusters obtained in discriminant analysis of principal components (DAPC) and sparse nonnegative matrix factorisation (SNMF) using 27 419 informative single nucleotide polymorphisms in whole genome data of 264 *Leptosphaeria maculans* isolates from the Western Cape

| **Isolate number^a^** | **Mating type** | **DAPC**  **cluster** | **SNMF**  **cluster** | **SNMF Admixture Coefficients P1** | **SNMF Admixture coefficients P2** |
| --- | --- | --- | --- | --- | --- |
| STE-U 9674 | MAT1-1 | 2 | 2 | 0,0589096 | 0,94109 |
| STE-U 9675 | MAT1-1 | 2 | 2 | 0,0001 | 0,9999 |
| STE-U 9679 | MAT1-1 | 2 | 2 | 0,0551956 | 0,944804 |
| STE-U 9680 | MAT1-1 | 2 | 2 | 0,0910084 | 0,908992 |
| STE-U 9681 | MAT1-1 | 2 | 2 | 0,0443151 | 0,955685 |
| STE-U 9682 | MAT1-1 | 2 | 2 | 0,0848973 | 0,915103 |
| STE-U 9683 | MAT1-2 | 2 | 2 | 0,101721 | 0,898279 |
| STE-U 9684 | MAT1-1 | 2 | 2 | 0,177927 | 0,822073 |
| STE-U 9685 | MAT1-1 | 2 | 2 | 0,109683 | 0,890317 |
| STE-U 9687 | MAT1-2 | 2 | 2 | 0,0196089 | 0,980391 |
| STE-U 9688 | MAT1-1 | 2 | 2 | 0,195474 | 0,804526 |
| STE-U 9689 | MAT1-1 | 2 | 2 | 0,0381168 | 0,961883 |
| STE-U 9690 | MAT1-2 | 2 | 2 | 0,0596978 | 0,940302 |
| STE-U 9691 | MAT1-1 | 2 | 2 | 0,137697 | 0,862303 |
| STE-U 9692 | MAT1-1 | 2 | 2 | 0,132457 | 0,867543 |
| STE-U 9693 | MAT1-2 | 2 | 2 | 0,145626 | 0,854374 |
| STE-U 9694 | MAT1-2 | 2 | 2 | 0,0995424 | 0,900458 |
| STE-U 9695 | MAT1-1 | 2 | 2 | 0,09135 | 0,90865 |
| STE-U 9696 | MAT1-2 | 2 | 2 | 0,237804 | 0,762196 |
| STE-U 9697 | MAT1-1 | 2 | 2 | 0,201921 | 0,798079 |
| STE-U 9698 | MAT1-2 | 2 | 2 | 0,144345 | 0,855655 |
| STE-U 9699 | MAT1-2 | 2 | 2 | 0,169883 | 0,830117 |
| STE-U 9700 | MAT1-1 | 2 | 2 | 0,154921 | 0,845079 |
| STE-U 9701 | MAT1-1 | 2 | 2 | 0,140568 | 0,859432 |
| STE-U 9702 | MAT1-2 | 2 | 2 | 0,172248 | 0,827752 |
| STE-U 9703 | MAT1-2 | 2 | 2 | 0,149001 | 0,850999 |
| STE-U 9704 | MAT1-1 | 2 | 2 | 0,187244 | 0,812756 |
| STE-U 9705 | MAT1-2 | 2 | 2 | 0,0935738 | 0,906426 |
| STE-U 9706^d^ | MAT1-2 | 2 | 2 | 0,115692 | 0,884308 |
| STE-U 9707 | MAT1-2 | 2 | 2 | 0,155474 | 0,844526 |
| STE-U 9708^d^ | MAT1-2 | 2 | 2 | 0,115692 | 0,884308 |
| STE-U 9709 | MAT1-2 | 1 | 1 | 0,9999 | 0,0001 |
| STE-U 9711 | MAT1-1 | 1 | 1 | 0,970006 | 0,0299942 |
| STE-U 9712 | MAT1-2 | 1 | 1 | 0,968918 | 0,031082 |
| STE-U 9715 | MAT1-1 | 1 | 1 | 0,839601 | 0,160399 |
| STE-U 9717 | MAT1-1 | 1 | 1 | 0,801666 | 0,198334 |
| STE-U 9718 | MAT1-2 | 1 | 1 | 0,983397 | 0,0166031 |
| STE-U 9719 | MAT1-2 | 1 | 1 | 0,933497 | 0,0665035 |
| STE-U 9720 | MAT1-2 | 1 | 1 | 0,912817 | 0,0871826 |
| STE-U 9721 | MAT1-2 | 1 | 1 | 0,9999 | 0,0001 |
| STE-U 9722 | MAT1-1 | 1 | 1 | 0,921299 | 0,0787012 |
| STE-U 9723 | MAT1-2 | 1 | 1 | 0,938114 | 0,0618859 |
| STE-U 9724^e^ | MAT1-1 | 1 | 1 | 0,860184 | 0,139816 |
| STE-U 9725^e^ | MAT1-1 | 1 | 1 | 0,860184 | 0,139816 |
| STE-U 9727 | MAT1-2 | 1 | 1 | 0,945604 | 0,054396 |
| STE-U 9729^f^ | MAT1-1 | 1 | 1 | 0,959481 | 0,040519 |
| STE-U 9730^f^ | MAT1-1 | 1 | 1 | 0,959481 | 0,040519 |
| STE-U 9731^f^ | MAT1-1 | 1 | 1 | 0,959481 | 0,040519 |
| STE-U 9732 | MAT1-1 | 2 | 2 | 0,23507 | 0,76493 |
| STE-U 9733 | MAT1-2 | 1 | 1 | 0,953569 | 0,0464311 |
| STE-U 9735^g^ | MAT1-2 | 1 | 1 | 0,9999 | 0,0001 |
| STE-U 9736^h^ | MAT1-2 | 1 | 1 | 0,868747 | 0,131253 |
| STE-U 9737^h^ | MAT1-2 | 1 | 1 | 0,868747 | 0,131253 |
| STE-U 9738^i^ | MAT1-1 | 1 | 1 | 0,790331 | 0,209669 |
| STE-U 9739^i^ | MAT1-1 | 1 | 1 | 0,790331 | 0,209669 |
| STE-U 9740^j^ | MAT1-1 | 1 | 1 | 0,807563 | 0,192437 |
| STE-U 9741^j^ | MAT1-1 | 1 | 1 | 0,807563 | 0,192437 |
| STE-U 9742^j^ | MAT1-1 | 1 | 1 | 0,807563 | 0,192437 |
| STE-U 9743^j^ | MAT1-1 | 1 | 1 | 0,807563 | 0,192437 |
| STE-U 9744 | MAT1-2 | 1 | 1 | 0,9999 | 0,0001 |
| STE-U 9745 | MAT1-1 | 2 | 2 | 0,162675 | 0,837325 |
| STE-U 9746 | MAT1-2 | 2 | 2 | 0,204524 | 0,795476 |
| STE-U 9747 | MAT1-2 | 2 | 2 | 0,157989 | 0,842011 |
| STE-U 9748 | MAT1-1 | 2 | 2 | 0,347365 | 0,652635 |
| STE-U 9749 | MAT1-2 | 2 | 2 | 0,115706 | 0,884294 |
| STE-U 9750 | MAT1-1 | 2 | 2 | 0,150837 | 0,849163 |
| STE-U 9751 | MAT1-2 | 2 | 2 | 0,390044 | 0,609956 |
| STE-U 9752 | MAT1-2 | 2 | 2 | 0,390046 | 0,609954 |
| STE-U 9753 | MAT1-2 | 2 | 2 | 0,132215 | 0,867785 |
| STE-U 9754 | MAT1-1 | 2 | 2 | 0,116826 | 0,883174 |
| STE-U 9755 | MAT1-1 | 2 | 2 | 0,0615232 | 0,938477 |
| STE-U 9756 | MAT1-1 | 2 | 2 | 0,186291 | 0,813709 |
| STE-U 9757 | MAT1-2 | 2 | 2 | 0,0563555 | 0,943644 |
| STE-U 9758 | MAT1-2 | 2 | 2 | 0,232979 | 0,767021 |
| STE-U 9759 | MAT1-1 | 2 | 2 | 0,180415 | 0,819585 |
| STE-U 9760 | MAT1-1 | 2 | 2 | 0,0317473 | 0,968253 |
| STE-U 9761 | MAT1-2 | 2 | 2 | 0,06825 | 0,93175 |
| STE-U 9762 | MAT1-2 | 2 | 2 | 0,106623 | 0,893377 |
| STE-U 9763 | MAT1-1 | 2 | 2 | 0,0193811 | 0,980619 |
| STE-U 9765 | MAT1-1 | 2 | 2 | 0,111871 | 0,888129 |
| STE-U 9766 | MAT1-2 | 2 | 2 | 0,0938048 | 0,906195 |
| STE-U 9767 | MAT1-1 | 2 | 2 | 0,0720111 | 0,927989 |
| STE-U 9768 | MAT1-2 | 2 | 2 | 0,0001 | 0,9999 |
| STE-U 9769 | MAT1-2 | 2 | 2 | 0,0835877 | 0,916412 |
| STE-U 9770 | MAT1-1 | 2 | 2 | 0,157134 | 0,842866 |
| STE-U 9771 | MAT1-1 | 2 | 2 | 0,113228 | 0,886772 |
| STE-U 9772 | MAT1-2 | 2 | 2 | 0,0109313 | 0,989069 |
| STE-U 9773 | MAT1-2 | 2 | 2 | 0,00323399 | 0,996766 |
| STE-U 9774 | MAT1-1 | 2 | 2 | 0,143458 | 0,856542 |
| STE-U 9775 | MAT1-2 | 2 | 2 | 0,0565576 | 0,943442 |
| STE-U 9776 | MAT1-2 | 2 | 2 | 0,136135 | 0,863865 |
| STE-U 9777 | MAT1-2 | 2 | 2 | 0,0447137 | 0,955286 |
| STE-U 9778 | MAT1-2 | 2 | 2 | 0,137312 | 0,862688 |
| STE-U 9779 | MAT1-2 | 2 | 2 | 0,262633 | 0,737367 |
| STE-U 9780 | MAT1-1 | 2 | 2 | 0,0949325 | 0,905067 |
| STE-U 9781 | MAT1-2 | 2 | 2 | 0,169107 | 0,830893 |
| STE-U 9782 | MAT1-2 | 2 | 2 | 0,231795 | 0,768205 |
| STE-U 9783 | MAT1-1 | 2 | 2 | 0,129876 | 0,870124 |
| STE-U 9785 | MAT1-2 | 2 | 2 | 0,245354 | 0,754646 |
| STE-U 9786 | MAT1-2 | 2 | 2 | 0,170579 | 0,829421 |
| STE-U 9787 | MAT1-1 | 2 | 2 | 0,171232 | 0,828768 |
| STE-U 9789 | MAT1-2 | 1 | 1 | 0,963252 | 0,0367484 |
| STE-U 9790 | MAT1-2 | 1 | 1 | 0,976088 | 0,0239123 |
| STE-U 9791 | MAT1-2 | 1 | 1 | 0,950633 | 0,0493674 |
| STE-U 9792 | MAT1-2 | 1 | 1 | 0,9999 | 0,0001 |
| STE-U 9793 | MAT1-2 | 1 | 1 | 0,981369 | 0,0186312 |
| STE-U 9794 | MAT1-2 | 1 | 1 | 0,870484 | 0,129516 |
| STE-U 9795 | MAT1-2 | 1 | 1 | 0,857174 | 0,142826 |
| STE-U 9796 | MAT1-2 | 1 | 1 | 0,974565 | 0,0254349 |
| STE-U 9797 | MAT1-2 | 1 | 1 | 0,9999 | 0,0001 |
| STE-U 9798 | MAT1-2 | 1 | 1 | 0,910214 | 0,0897862 |
| STE-U 9799 | MAT1-1 | 1 | 1 | 0,974243 | 0,0257566 |
| STE-U 9800 | MAT1-1 | 1 | 1 | 0,962984 | 0,037016 |
| STE-U 9801 | MAT1-1 | 1 | 1 | 0,940555 | 0,0594451 |
| STE-U 9802 | MAT1-1 | 1 | 1 | 0,955312 | 0,0446882 |
| STE-U 9803 | MAT1-1 | 2 | 2 | 0,185663 | 0,814337 |
| STE-U 9804 | MAT1-1 | 2 | 2 | 0,205488 | 0,794512 |
| STE-U 9805 | MAT1-1 | 2 | 2 | 0,0890086 | 0,910991 |
| STE-U 9806 | MAT1-1 | 2 | 2 | 0,0545099 | 0,94549 |
| STE-U 9807 | MAT1-1 | 2 | 2 | 0,247931 | 0,752069 |
| STE-U 9808 | MAT1-2 | 2 | 2 | 0,174758 | 0,825242 |
| STE-U 9809 | MAT1-2 | 2 | 2 | 0,173273 | 0,826727 |
| STE-U 9810 | MAT1-2 | 2 | 2 | 0,37883 | 0,62117 |
| STE-U 9811 | MAT1-2 | 1 | 1 | 0,985479 | 0,0145209 |
| STE-U 9812 | MAT1-1 | 1 | 1 | 0,896736 | 0,103264 |
| STE-U 9813 | MAT1-2 | 1 | 1 | 0,918209 | 0,0817911 |
| STE-U 9814 | MAT1-2 | 1 | 1 | 0,934269 | 0,0657305 |
| STE-U 9815 | MAT1-1 | 1 | 1 | 0,9999 | 0,0001 |
| STE-U 9816 | MAT1-2 | 1 | 1 | 0,973531 | 0,0264688 |
| STE-U 9817 | MAT1-2 | 1 | 1 | 0,977189 | 0,0228115 |
| STE-U 9818 | MAT1-1 | 1 | 1 | 0,883242 | 0,116758 |
| STE-U 9819 | MAT1-1 | 1 | 1 | 0,959758 | 0,0402417 |
| STE-U 9820 | MAT1-2 | 1 | 1 | 0,963257 | 0,0367433 |
| STE-U 9821 | MAT1-1 | 1 | 1 | 0,98074 | 0,0192599 |
| STE-U 9822 | MAT1-1 | 1 | 1 | 0,818354 | 0,181646 |
| STE-U 9823 | MAT1-2 | 1 | 1 | 0,929315 | 0,0706845 |
| STE-U 9824 | MAT1-1 | 1 | 1 | 0,800855 | 0,199145 |
| STE-U 9825 | MAT1-2 | 1 | 1 | 0,856948 | 0,143052 |
| STE-U 9827 | MAT1-2 | 1 | 1 | 0,920574 | 0,0794257 |
| STE-U 9828 | MAT1-2 | 1 | 1 | 0,860076 | 0,139924 |
| STE-U 9829 | MAT1-2 | 1 | 1 | 0,883754 | 0,116246 |
| STE-U 9830 | MAT1-2 | 1 | 1 | 0,946909 | 0,0530914 |
| STE-U 9831 | MAT1-1 | 2 | 2 | 0,227355 | 0,772645 |
| STE-U 9832 | MAT1-1 | 1 | 1 | 0,813694 | 0,186306 |
| STE-U 9833^k^ | MAT1-1 | 1 | 1 | 0,88311 | 0,11689 |
| STE-U 9834^k^ | MAT1-1 | 1 | 1 | 0,88311 | 0,11689 |
| STE-U 9836 | MAT1-1 | 1 | 1 | 0,904858 | 0,0951415 |
| STE-U 9837 | MAT1-1 | 1 | 1 | 0,821442 | 0,178558 |
| STE-U 9838 | MAT1-2 | 1 | 1 | 0,763345 | 0,236655 |
| STE-U 9839 | MAT1-2 | 1 | 1 | 0,761478 | 0,238522 |
| STE-U 9840 | MAT1-2 | 2 | 2 | 0,115501 | 0,884499 |
| STE-U 9841 | MAT1-2 | 2 | 2 | 0,0422152 | 0,957785 |
| STE-U 9842 | MAT1-1 | 2 | 2 | 0,0462967 | 0,953703 |
| STE-U 9843 | MAT1-1 | 2 | 2 | 0,138686 | 0,861314 |
| STE-U 9844 | MAT1-1 | 2 | 2 | 0,14807 | 0,85193 |
| STE-U 9845 | MAT1-1 | 2 | 2 | 0,130337 | 0,869663 |
| STE-U 9846 | MAT1-2 | 2 | 2 | 0,0763185 | 0,923681 |
| STE-U 9847 | MAT1-1 | 2 | 2 | 0,198653 | 0,801347 |
| STE-U 9848 | MAT1-2 | 2 | 2 | 0,0509477 | 0,949052 |
| STE-U 9849 | MAT1-2 | 2 | 2 | 0,115197 | 0,884803 |
| STE-U 9850 | MAT1-1 | 2 | 2 | 0,101205 | 0,898795 |
| STE-U 9851 | MAT1-1 | 2 | 2 | 0,128403 | 0,871597 |
| STE-U 9852 | MAT1-1 | 2 | 2 | 0,230685 | 0,769315 |
| STE-U 9854 | MAT1-1 | 2 | 2 | 0,0310496 | 0,96895 |
| STE-U 9855 | MAT1-2 | 2 | 2 | 0,172779 | 0,827221 |
| STE-U 9856 | MAT1-2 | 2 | 2 | 0,210905 | 0,789095 |
| STE-U 9857 | MAT1-1 | 2 | 2 | 0,385803 | 0,614197 |
| STE-U 9858 | MAT1-2 | 2 | 2 | 0,120396 | 0,879604 |
| STE-U 9859 | MAT1-2 | 2 | 2 | 0,186501 | 0,813499 |
| STE-U 9860 | MAT1-2 | 2 | 2 | 0,136175 | 0,863825 |
| STE-U 9861 | MAT1-1 | 2 | 2 | 0,124757 | 0,875243 |
| STE-U 9862 | MAT1-1 | 2 | 2 | 0,194131 | 0,805869 |
| STE-U 9863 | NA | 2 | 2 | 0,0591193 | 0,940881 |
| STE-U 9864 | MAT1-1 | 2 | 2 | 0,162503 | 0,837497 |
| STE-U 9865 | MAT1-1 | 2 | 2 | 0,0111959 | 0,988804 |
| STE-U 9866 | MAT1-2 | 2 | 2 | 0,119284 | 0,880716 |
| STE-U 9867 | MAT1-1 | 2 | 2 | 0,171105 | 0,828895 |
| STE-U 9868 | MAT1-2 | 1 | 1 | 0,973724 | 0,0262755 |
| STE-U 9869 | MAT1-2 | 1 | 1 | 0,871085 | 0,128915 |
| STE-U 9870 | MAT1-2 | 1 | 1 | 0,911039 | 0,0889609 |
| STE-U 9871 | MAT1-2 | 1 | 1 | 0,778371 | 0,221629 |
| STE-U 9872 | MAT1-1 | 1 | 1 | 0,830039 | 0,169961 |
| STE-U 9873 | MAT1-2 | 1 | 1 | 0,914204 | 0,0857961 |
| STE-U 9874 | MAT1-1 | 1 | 1 | 0,98989 | 0,0101103 |
| STE-U 9875 | MAT1-1 | 1 | 1 | 0,811939 | 0,188061 |
| STE-U 9876 | MAT1-1 | 1 | 1 | 0,880257 | 0,119743 |
| STE-U 9880 | MAT1-2 | 1 | 1 | 0,911425 | 0,0885754 |
| STE-U 9881 | MAT1-2 | 1 | 1 | 0,903397 | 0,0966035 |
| STE-U 9882 | MAT1-1 | 1 | 1 | 0,823628 | 0,176372 |
| STE-U 9883 | MAT1-1 | 1 | 1 | 0,853252 | 0,146748 |
| STE-U 9884 | MAT1-2 | 1 | 1 | 0,971552 | 0,0284483 |
| STE-U 9885 | MAT1-1 | 2 | 2 | 0,150942 | 0,849058 |
| STE-U 9886 | MAT1-2 | 2 | 2 | 0,262095 | 0,737905 |
| STE-U 9887 | MAT1-1 | 2 | 2 | 0,274058 | 0,725942 |
| STE-U 9888 | MAT1-2 | 2 | 2 | 0,102521 | 0,897479 |
| STE-U 9889 | MAT1-1 | 2 | 2 | 0,129247 | 0,870753 |
| STE-U 9890 | MAT1-1 | 2 | 2 | 0,267735 | 0,732265 |
| STE-U 9891 | MAT1-1 | 2 | 2 | 0,0924871 | 0,907513 |
| STE-U 9892 | MAT1-1 | 2 | 2 | 0,204655 | 0,795345 |
| STE-U 9893 | MAT1-1 | 2 | 2 | 0,223307 | 0,776693 |
| STE-U 9894 | MAT1-1 | 2 | 2 | 0,109566 | 0,890434 |
| STE-U 9896 | MAT1-2 | 2 | 2 | 0,085848 | 0,914152 |
| STE-U 9897 | MAT1-1 | 1 | 1 | 0,859205 | 0,140795 |
| STE-U 9898 | NA | 1 | 1 | 0,94454 | 0,0554599 |
| STE-U 9899 | MAT1-1 | 1 | 1 | 0,911235 | 0,0887653 |
| STE-U 9900 | MAT1-1 | 1 | 1 | 0,789216 | 0,210784 |
| STE-U 9901 | MAT1-2 | 1 | 1 | 0,791958 | 0,208042 |
| STE-U 9902 | MAT1-1 | 1 | 1 | 0,9999 | 0,0001 |
| STE-U 9903 | MAT1-2 | 1 | 1 | 0,937782 | 0,0622179 |
| STE-U 9904 | MAT1-2 | 1 | 1 | 0,912496 | 0,0875039 |
| STE-U 9905 | MAT1-1 | 1 | 1 | 0,568985 | 0,431015 |
| STE-U 9906 | MAT1-1 | 1 | 1 | 0,978143 | 0,0218571 |
| STE-U 9907 | MAT1-2 | 1 | 1 | 0,974298 | 0,025702 |
| STE-U 9908 | MAT1-2 | 1 | 1 | 0,991895 | 0,00810535 |
| STE-U 9910 | MAT1-1 | 1 | 1 | 0,866056 | 0,133944 |
| STE-U 9912 | MAT1-1 | 1 | 1 | 0,992209 | 0,00779103 |
| STE-U 9914 | MAT1-1 | 1 | 1 | 0,876039 | 0,123961 |
| STE-U 9915 | MAT1-1 | 1 | 1 | 0,863601 | 0,136399 |
| STE-U 9916 | MAT1-1 | 1 | 1 | 0,931057 | 0,0689432 |
| STE-U 9918 | MAT1-2 | 1 | 1 | 0,839471 | 0,160529 |
| STE-U 9919 | MAT1-1 | 1 | 1 | 0,933004 | 0,0669963 |
| STE-U 9920 | MAT1-2 | 1 | 1 | 0,927433 | 0,0725665 |
| STE-U 9921 | MAT1-1 | 1 | 1 | 0,960297 | 0,0397032 |
| STE-U 9922 | MAT1-2 | 1 | 1 | 0,885044 | 0,114956 |
| STE-U 9923 | MAT1-1 | 1 | 1 | 0,664747 | 0,335253 |
| STE-U 9925 | MAT1-1 | 1 | 1 | 0,806935 | 0,193065 |
| STE-U 9927 | MAT1-1 | 1 | 1 | 0,864827 | 0,135173 |
| STE-U 9928 | MAT1-1 | 1 | 1 | 0,789971 | 0,210029 |
| STE-U 9929 | MAT1-1 | 1 | 1 | 0,719696 | 0,280304 |
| STE-U 9930 | MAT1-1 | 1 | 1 | 0,668396 | 0,331604 |
| IBCN241 | MAT1-1 | 1 | 1 | 0,948451 | 0,051549 |
| IBCN242^l^ | MAT1-1 | 1 | 1 | 0,781382 | 0,218618 |
| IBCN243^l^ | MAT1-1 | 1 | 1 | 0,781382 | 0,218618 |
| IBCN244 | MAT1-2 | 1 | 1 | 0,968595 | 0,0314055 |
| IBCN245 | MAT1-2 | 1 | 1 | 0,9999 | 0,0001 |
| IBCN246 | MAT1-2 | 2 | 2 | 0,147966 | 0,852034 |
| IBCN247 | MAT1-2 | 2 | 2 | 0,242891 | 0,757109 |
| IBCN249 | MAT1-1 | 1 | 1 | 0,969633 | 0,0303669 |
| IBCN250 | MAT1-2 | 1 | 1 | 0,843149 | 0,156851 |
| IBCN251^g^ | MAT1-2 | 1 | 1 | 0,9999 | 0,0001 |
| IBCN252 | MAT1-1 | 2 | 2 | 0,0421048 | 0,957895 |
| IBCN253 | MAT1-1 | 1 | 1 | 0,885443 | 0,114557 |
| IBCN254 | MAT1-1 | 2 | 2 | 0,126328 | 0,873672 |
| IBCN255 | MAT1-2 | 2 | 2 | 0,152929 | 0,847071 |
| IBCN256 | MAT1-2 | 2 | 2 | 0,19417 | 0,80583 |
| IBCN257 | MAT1-1 | 2 | 2 | 0,249381 | 0,750619 |
| IBCN258 | MAT1-2 | 2 | 2 | 0,185115 | 0,814885 |
| IBCN259 | MAT1-2 | 2 | 2 | 0,0809623 | 0,919038 |
| IBCN260 | MAT1-2 | 2 | 2 | 0,158338 | 0,841662 |
| IBCN261 | MAT1-1 | 2 | 2 | 0,0476701 | 0,95233 |
| IBCN262 | MAT1-2 | 1 | 1 | 0,980455 | 0,0195446 |
| IBCN263 | MAT1-2 | 1 | 1 | 0,935582 | 0,0644175 |
| IBCN264 | MAT1-1 | 1 | 1 | 0,879341 | 0,120659 |
| IBCN265 | MAT1-2 | 1 | 1 | 0,917171 | 0,0828289 |
| IBCN266 | MAT1-2 | 1 | 1 | 0,932635 | 0,067365 |
| IBCN267 | MAT1-1 | 1 | 1 | 0,836984 | 0,163016 |
| IBCN268 | MAT1-2 | 1 | 1 | 0,9999 | 0,0001 |
| IBCN269 | MAT1-2 | 2 | 2 | 0,0864957 | 0,913504 |
| IBCN270 | MAT1-2 | 2 | 2 | 0,169367 | 0,830633 |
| IBCN271 | MAT1-2 | 2 | 2 | 0,171324 | 0,828676 |
| IBCN272 | MAT1-1 | 2 | 2 | 0,0680443 | 0,931956 |
| IBCN273 | MAT1-1 | 2 | 2 | 0,132883 | 0,867117 |
| IBCN274 | MAT1-1 | 2 | 2 | 0,133275 | 0,866725 |
| IBCN275 | MAT1-2 | 1 | 1 | 0,935329 | 0,0646715 |

^a^Isolates with the same superscript letter was collapsed into one multilocus lineage
