# Supplementary material for: Whole genome sequencing reveals the genetic diversity and structure of Leptosphaeria maculans populations from the Western Cape province of South Africa
Source: BMC Genomics. 2025 Apr 3;26:334. doi: 10.1186/s12864-025-11413-3 (PMC11966903; doi:10.1186/s12864-025-11413-3)
Supplement: Supplementary file 6 — Supplementary Material 6 [file 12864_2025_11413_MOESM6_ESM.docx]

**Table S6** Information on clusters obtained in discriminant analysis of principal components (DAPC) and sparse nonnegative matrix factorisation (SNMF) using 54 864 informative single nucleotide polymorphisms in whole genome data of 435 international *Leptosphaeria maculans* isolates

| **Isolate number^a^** | **DAPC cluster** | **SNMF cluster** | **SNMF Admixture Coefficients P1** | **SNMF Admixture Coefficients P2** | **SNMF Admixture Coefficients P3** | **SNMF Admixture Coefficients P4** | **SNMF Admixture Coefficients P5** |
| --- | --- | --- | --- | --- | --- | --- | --- |
| STE-U 9674 | 5 | 5 | 1E-04 | 0,003775 | 0,010513 | 0,169999 | 0,815613 |
| STE-U 9675 | 5 | 5 | 0,020901 | 0,108022 | 1E-04 | 0,084825 | 0,786152 |
| STE-U 9679 | 5 | 5 | 0,021015 | 0,026309 | 0,051911 | 0,164681 | 0,736084 |
| STE-U 9680 | 5 | 5 | 0,032641 | 0,000762 | 0,006505 | 0,142513 | 0,817578 |
| STE-U 9681 | 5 | 5 | 0,016196 | 1E-04 | 0,026804 | 0,113249 | 0,843652 |
| STE-U 9682 | 5 | 5 | 0,002186 | 0,018954 | 0,051776 | 0,167049 | 0,760035 |
| STE-U 9683 | 5 | 5 | 0,023163 | 0,021726 | 0,035035 | 0,164717 | 0,755359 |
| STE-U 9684 | 5 | 5 | 0,012912 | 0,014021 | 0,017137 | 0,227895 | 0,728034 |
| STE-U 9685 | 5 | 5 | 1E-04 | 1E-04 | 1E-04 | 0,178619 | 0,821081 |
| STE-U 9687 | 5 | 5 | 0,017537 | 0,022072 | 1E-04 | 0,138529 | 0,821762 |
| STE-U 9688 | 5 | 5 | 0,014513 | 1E-04 | 0,051645 | 0,261037 | 0,672705 |
| STE-U 9689 | 5 | 5 | 0,000806 | 0,092907 | 0,029368 | 0,166018 | 0,710902 |
| STE-U 9690 | 5 | 5 | 1E-04 | 0,059651 | 0,03341 | 0,143439 | 0,7634 |
| STE-U 9691 | 5 | 5 | 0,005832 | 0,049969 | 1E-04 | 0,200792 | 0,743308 |
| STE-U 9692 | 5 | 5 | 0,039315 | 0,036325 | 0,011935 | 0,218284 | 0,694142 |
| STE-U 9693 | 5 | 5 | 0,005971 | 0,020537 | 0,070846 | 0,189739 | 0,712906 |
| STE-U 9694 | 5 | 5 | 1E-04 | 1E-04 | 0,011937 | 0,224455 | 0,763409 |
| STE-U 9695 | 5 | 5 | 0,020326 | 0,069548 | 0,007659 | 0,197872 | 0,704595 |
| STE-U 9696 | 5 | 5 | 0,024956 | 1E-04 | 0,026976 | 0,297598 | 0,65037 |
| STE-U 9697 | 5 | 5 | 1E-04 | 0,086348 | 0,024149 | 0,245358 | 0,644045 |
| STE-U 9698 | 5 | 5 | 0,015222 | 0,016885 | 1E-04 | 0,263029 | 0,704765 |
| STE-U 9699 | 5 | 5 | 0,00098 | 1E-04 | 0,013233 | 0,224781 | 0,760906 |
| STE-U 9700 | 5 | 5 | 0,041314 | 0,02005 | 0,009329 | 0,228281 | 0,701025 |
| STE-U 9701 | 5 | 5 | 0,027734 | 0,032373 | 1E-04 | 0,283406 | 0,656388 |
| STE-U 9702 | 5 | 5 | 1E-04 | 0,079769 | 0,0293 | 0,27104 | 0,61979 |
| STE-U 9703 | 5 | 5 | 0,027341 | 0,102168 | 0,017034 | 0,214621 | 0,638836 |
| STE-U 9704 | 5 | 5 | 0,014727 | 1E-04 | 0,025446 | 0,232488 | 0,727239 |
| STE-U 9705 | 5 | 5 | 0,008164 | 0,010604 | 0,043692 | 0,222928 | 0,714613 |
| STE-U 9706^d^ | 5 | 5 | 0,020433 | 0,080854 | 0,032002 | 0,19935 | 0,667361 |
| STE-U 9707 | 5 | 5 | 0,006132 | 1E-04 | 0,00736 | 0,213119 | 0,77329 |
| STE-U 9708^d^ | 5 | 5 | 0,020433 | 0,080854 | 0,032002 | 0,19935 | 0,667361 |
| STE-U 9709 | 1 | 4 | 0,018762 | 0,01052 | 0,019819 | 0,950799 | 1E-04 |
| STE-U 9711 | 1 | 4 | 0,002221 | 0,033076 | 0,015299 | 0,927035 | 0,02237 |
| STE-U 9712 | 1 | 4 | 0,012487 | 0,000272 | 0,014081 | 0,973061 | 1E-04 |
| STE-U 9715 | 1 | 4 | 0,014888 | 0,02695 | 0,074798 | 0,835326 | 0,048039 |
| STE-U 9717 | 1 | 4 | 0,005264 | 1E-04 | 0,01299 | 0,857128 | 0,124519 |
| STE-U 9718 | 1 | 4 | 0,028907 | 0,056354 | 1E-04 | 0,91454 | 1E-04 |
| STE-U 9719 | 1 | 4 | 0,000789 | 0,026135 | 0,024922 | 0,901721 | 0,046433 |
| STE-U 9720 | 1 | 4 | 0,011656 | 0,006659 | 0,029721 | 0,931131 | 0,020832 |
| STE-U 9721 | 1 | 4 | 0,020001 | 1E-04 | 1E-04 | 0,9797 | 1E-04 |
| STE-U 9722 | 1 | 4 | 1E-04 | 0,00942 | 0,00465 | 0,917719 | 0,068111 |
| STE-U 9723 | 1 | 4 | 0,023362 | 1E-04 | 0,017329 | 0,925553 | 0,033657 |
| STE-U 9724^e^ | 1 | 4 | 0,017357 | 0,033959 | 1E-04 | 0,854043 | 0,094541 |
| STE-U 9725^e^ | 1 | 4 | 0,017357 | 0,033959 | 1E-04 | 0,854043 | 0,094541 |
| STE-U 9727 | 1 | 4 | 1E-04 | 0,029428 | 0,018444 | 0,931848 | 0,02018 |
| STE-U 9729^f^ | 1 | 4 | 0,027927 | 0,012662 | 0,052561 | 0,90675 | 1E-04 |
| STE-U 9730^f^ | 1 | 4 | 0,027927 | 0,012662 | 0,052561 | 0,90675 | 1E-04 |
| STE-U 9731^f^ | 1 | 4 | 0,027927 | 0,012662 | 0,052561 | 0,90675 | 1E-04 |
| STE-U 9732 | 5 | 5 | 0,001356 | 0,063425 | 0,040746 | 0,278133 | 0,61634 |
| STE-U 9733 | 1 | 4 | 1E-04 | 1E-04 | 0,022331 | 0,871564 | 0,105905 |
| STE-U 9735^g^ | 1 | 4 | 0,005157 | 0,026448 | 0,002057 | 0,966238 | 1E-04 |
| STE-U 9736^h^ | 1 | 4 | 0,016445 | 0,041069 | 0,000933 | 0,848954 | 0,092599 |
| STE-U 9737^h^ | 1 | 4 | 0,016445 | 0,041069 | 0,000933 | 0,848954 | 0,092599 |
| STE-U 9738^i^ | 1 | 4 | 0,004649 | 0,062235 | 1E-04 | 0,809283 | 0,123733 |
| STE-U 9739^i^ | 1 | 4 | 0,004649 | 0,062235 | 1E-04 | 0,809283 | 0,123733 |
| STE-U 9740^j^ | 1 | 4 | 0,000128 | 0,067344 | 0,034097 | 0,799931 | 0,098499 |
| STE-U 9741^j^ | 1 | 4 | 0,000128 | 0,067344 | 0,034097 | 0,799931 | 0,098499 |
| STE-U 9742^j^ | 1 | 4 | 0,000128 | 0,067344 | 0,034097 | 0,799931 | 0,098499 |
| STE-U 9743^j^ | 1 | 4 | 0,000128 | 0,067344 | 0,034097 | 0,799931 | 0,098499 |
| STE-U 9744 | 1 | 4 | 0,001966 | 1E-04 | 0,035848 | 0,911011 | 0,051075 |
| STE-U 9745 | 5 | 5 | 0,013774 | 0,054035 | 0,029056 | 0,26577 | 0,637365 |
| STE-U 9746 | 5 | 5 | 0,00285 | 0,034758 | 0,000596 | 0,260976 | 0,700819 |
| STE-U 9747 | 5 | 5 | 1E-04 | 0,043734 | 1E-04 | 0,231556 | 0,724509 |
| STE-U 9748 | 5 | 5 | 0,047793 | 1E-04 | 0,019165 | 0,415554 | 0,517388 |
| STE-U 9749 | 5 | 5 | 0,01095 | 0,034647 | 0,032584 | 0,209593 | 0,712226 |
| STE-U 9750 | 5 | 5 | 0,001692 | 0,029887 | 0,009924 | 0,25442 | 0,704077 |
| STE-U 9751 | 5 | 5 | 1E-04 | 1E-04 | 0,023339 | 0,458828 | 0,517634 |
| STE-U 9752 | 5 | 5 | 1E-04 | 1E-04 | 0,044484 | 0,439053 | 0,516264 |
| STE-U 9753 | 5 | 5 | 0,020672 | 0,021218 | 0,02699 | 0,233592 | 0,697529 |
| STE-U 9754 | 5 | 5 | 0,008001 | 0,042895 | 0,064956 | 0,189437 | 0,694711 |
| STE-U 9755 | 5 | 5 | 0,027614 | 1E-04 | 0,027664 | 0,207679 | 0,736943 |
| STE-U 9756 | 5 | 5 | 1E-04 | 0,044766 | 0,022042 | 0,203219 | 0,729873 |
| STE-U 9757 | 5 | 5 | 0,001285 | 0,013137 | 0,032516 | 0,169151 | 0,783911 |
| STE-U 9758 | 5 | 5 | 0,042497 | 1E-04 | 0,008245 | 0,324051 | 0,625106 |
| STE-U 9759 | 5 | 5 | 0,019966 | 1E-04 | 0,025045 | 0,314568 | 0,640322 |
| STE-U 9760 | 5 | 5 | 0,008202 | 1E-04 | 0,000234 | 0,131375 | 0,860089 |
| STE-U 9761 | 5 | 5 | 0,031081 | 0,016127 | 0,087515 | 0,126453 | 0,738825 |
| STE-U 9762 | 5 | 5 | 1E-04 | 0,079282 | 0,014714 | 0,209724 | 0,69618 |
| STE-U 9763 | 5 | 5 | 1E-04 | 1E-04 | 1E-04 | 0,135028 | 0,864672 |
| STE-U 9765 | 5 | 5 | 0,005937 | 0,052228 | 0,029705 | 0,217237 | 0,694893 |
| STE-U 9766 | 5 | 5 | 0,019859 | 0,019151 | 0,035217 | 0,199219 | 0,726553 |
| STE-U 9767 | 5 | 5 | 1E-04 | 0,002516 | 1E-04 | 0,216825 | 0,78046 |
| STE-U 9768 | 5 | 5 | 0,019423 | 0,013196 | 0,000733 | 0,141508 | 0,82514 |
| STE-U 9769 | 5 | 5 | 0,012956 | 1E-04 | 0,009804 | 0,187431 | 0,789709 |
| STE-U 9770 | 5 | 5 | 1E-04 | 0,012451 | 1E-04 | 0,250759 | 0,73659 |
| STE-U 9771 | 5 | 5 | 1E-04 | 0,034602 | 0,015965 | 0,18251 | 0,766823 |
| STE-U 9772 | 5 | 5 | 0,001353 | 0,067546 | 0,010876 | 0,123963 | 0,796263 |
| STE-U 9773 | 5 | 5 | 0,018364 | 0,01901 | 0,001399 | 0,118946 | 0,842281 |
| STE-U 9774 | 5 | 5 | 0,002577 | 0,009139 | 0,008521 | 0,242127 | 0,737636 |
| STE-U 9775 | 5 | 5 | 0,006191 | 0,029701 | 0,020893 | 0,168023 | 0,775191 |
| STE-U 9776 | 5 | 5 | 1E-04 | 0,06616 | 0,011829 | 0,202628 | 0,719283 |
| STE-U 9777 | 5 | 5 | 0,008974 | 0,129131 | 0,020973 | 0,152732 | 0,68819 |
| STE-U 9778 | 5 | 5 | 1E-04 | 0,089998 | 0,033488 | 0,213961 | 0,662453 |
| STE-U 9779 | 5 | 5 | 0,036756 | 0,041789 | 1E-04 | 0,328294 | 0,593061 |
| STE-U 9780 | 5 | 5 | 1E-04 | 0,013172 | 1E-04 | 0,194441 | 0,792187 |
| STE-U 9781 | 5 | 5 | 0,000576 | 0,032576 | 1E-04 | 0,262536 | 0,704213 |
| STE-U 9782 | 5 | 5 | 0,027746 | 0,071744 | 1E-04 | 0,351184 | 0,549226 |
| STE-U 9783 | 5 | 5 | 0,022336 | 0,047099 | 0,033137 | 0,23198 | 0,665449 |
| STE-U 9785 | 5 | 5 | 0,038819 | 0,091862 | 1E-04 | 0,296394 | 0,572826 |
| STE-U 9786 | 5 | 5 | 0,00456 | 0,045603 | 0,015267 | 0,271465 | 0,663106 |
| STE-U 9787 | 5 | 5 | 0,015873 | 0,041243 | 0,003664 | 0,25998 | 0,679239 |
| STE-U 9789 | 1 | 4 | 0,008276 | 1E-04 | 1E-04 | 0,931187 | 0,060337 |
| STE-U 9790 | 1 | 4 | 0,036088 | 0,002219 | 0,017791 | 0,942712 | 0,001189 |
| STE-U 9791 | 1 | 4 | 0,001148 | 0,018746 | 0,015349 | 0,947687 | 0,01707 |
| STE-U 9792 | 1 | 4 | 1E-04 | 1E-04 | 0,012886 | 0,986815 | 1E-04 |
| STE-U 9793 | 1 | 4 | 0,006749 | 0,027706 | 0,000236 | 0,936075 | 0,029235 |
| STE-U 9794 | 1 | 4 | 0,015939 | 1E-04 | 0,053871 | 0,881236 | 0,048855 |
| STE-U 9795 | 1 | 4 | 0,002875 | 0,065636 | 1E-04 | 0,868699 | 0,06269 |
| STE-U 9796 | 1 | 4 | 1E-04 | 0,049845 | 0,007344 | 0,942611 | 1E-04 |
| STE-U 9797 | 1 | 4 | 1E-04 | 1E-04 | 1E-04 | 0,972323 | 0,027378 |
| STE-U 9798 | 1 | 4 | 1E-04 | 1E-04 | 0,02707 | 0,888456 | 0,084275 |
| STE-U 9799 | 1 | 4 | 0,015647 | 0,015837 | 0,00973 | 0,958686 | 1E-04 |
| STE-U 9800 | 1 | 4 | 1E-04 | 1E-04 | 0,032652 | 0,924967 | 0,042181 |
| STE-U 9801 | 1 | 4 | 0,0254 | 1E-04 | 0,026926 | 0,923117 | 0,024457 |
| STE-U 9802 | 1 | 4 | 1E-04 | 0,006008 | 1E-04 | 0,948163 | 0,04563 |
| STE-U 9803 | 5 | 5 | 0,003891 | 0,06443 | 0,018539 | 0,247755 | 0,665386 |
| STE-U 9804 | 5 | 5 | 0,024047 | 0,065456 | 0,002109 | 0,350283 | 0,558104 |
| STE-U 9805 | 5 | 5 | 1E-04 | 0,051849 | 1E-04 | 0,218143 | 0,729809 |
| STE-U 9806 | 5 | 5 | 0,013066 | 0,032757 | 0,030737 | 0,197173 | 0,726268 |
| STE-U 9807 | 5 | 5 | 1E-04 | 0,004106 | 0,010977 | 0,305766 | 0,679052 |
| STE-U 9808 | 5 | 5 | 0,010653 | 0,025154 | 0,011436 | 0,290505 | 0,662252 |
| STE-U 9809 | 5 | 5 | 0,010698 | 0,075334 | 0,000168 | 0,244753 | 0,669048 |
| STE-U 9810 | 5 | 4 | 0,015586 | 0,088467 | 1E-04 | 0,468959 | 0,426888 |
| STE-U 9811 | 1 | 4 | 0,008211 | 1E-04 | 0,038421 | 0,953168 | 1E-04 |
| STE-U 9812 | 1 | 4 | 0,026063 | 0,045282 | 0,010853 | 0,917702 | 1E-04 |
| STE-U 9813 | 1 | 4 | 0,023682 | 0,009771 | 0,024841 | 0,887945 | 0,053761 |
| STE-U 9814 | 1 | 4 | 0,039545 | 0,024355 | 1E-04 | 0,918794 | 0,017206 |
| STE-U 9815 | 1 | 4 | 0,020122 | 1E-04 | 1E-04 | 0,979578 | 1E-04 |
| STE-U 9816 | 1 | 4 | 0,011851 | 1E-04 | 0,004359 | 0,949367 | 0,034323 |
| STE-U 9817 | 1 | 4 | 1E-04 | 1E-04 | 0,034736 | 0,949443 | 0,015621 |
| STE-U 9818 | 1 | 4 | 0,020325 | 0,005046 | 0,013885 | 0,888648 | 0,072095 |
| STE-U 9819 | 1 | 4 | 0,018336 | 0,002662 | 0,02912 | 0,949782 | 1E-04 |
| STE-U 9820 | 1 | 4 | 0,026495 | 1E-04 | 0,028049 | 0,945256 | 1E-04 |
| STE-U 9821 | 1 | 4 | 0,016553 | 0,002521 | 0,008236 | 0,959676 | 0,013014 |
| STE-U 9822 | 1 | 4 | 0,000394 | 0,022178 | 0,035945 | 0,795084 | 0,146399 |
| STE-U 9823 | 1 | 4 | 0,022462 | 0,021648 | 1E-04 | 0,902771 | 0,05302 |
| STE-U 9824 | 1 | 4 | 0,001852 | 0,016114 | 1E-04 | 0,837996 | 0,143938 |
| STE-U 9825 | 1 | 4 | 0,006889 | 0,004891 | 0,045041 | 0,844883 | 0,098296 |
| STE-U 9827 | 1 | 4 | 0,002882 | 1E-04 | 0,030167 | 0,891268 | 0,075583 |
| STE-U 9828 | 1 | 4 | 1E-04 | 0,083163 | 0,040429 | 0,865876 | 0,010432 |
| STE-U 9829 | 1 | 4 | 0,013491 | 1E-04 | 0,029065 | 0,878493 | 0,078851 |
| STE-U 9830 | 1 | 4 | 1E-04 | 1E-04 | 0,033504 | 0,959203 | 0,007093 |
| STE-U 9831 | 5 | 5 | 0,029822 | 0,055038 | 0,016625 | 0,268007 | 0,630508 |
| STE-U 9832 | 1 | 4 | 0,004166 | 0,036949 | 0,033482 | 0,782395 | 0,143008 |
| STE-U 9833^k^ | 1 | 4 | 0,008407 | 1E-04 | 0,029147 | 0,930442 | 0,031904 |
| STE-U 9834^k^ | 1 | 4 | 0,008407 | 1E-04 | 0,029147 | 0,930442 | 0,031904 |
| STE-U 9836 | 1 | 4 | 0,001181 | 1E-04 | 0,016021 | 0,891267 | 0,091431 |
| STE-U 9837 | 1 | 4 | 0,017056 | 0,030708 | 1E-04 | 0,825339 | 0,126797 |
| STE-U 9838 | 1 | 4 | 0,032844 | 0,031958 | 1E-04 | 0,751835 | 0,183263 |
| STE-U 9839 | 1 | 4 | 1E-04 | 0,042028 | 0,019469 | 0,801328 | 0,137075 |
| STE-U 9840 | 5 | 5 | 0,013651 | 0,033492 | 0,03152 | 0,182845 | 0,738491 |
| STE-U 9841 | 5 | 5 | 0,023789 | 0,060183 | 0,014457 | 0,101689 | 0,799881 |
| STE-U 9842 | 5 | 5 | 0,021631 | 0,049728 | 1E-04 | 0,179949 | 0,748592 |
| STE-U 9843 | 5 | 5 | 1E-04 | 0,074383 | 0,01969 | 0,282491 | 0,623336 |
| STE-U 9844 | 5 | 5 | 1E-04 | 0,027187 | 1E-04 | 0,232729 | 0,739885 |
| STE-U 9845 | 5 | 5 | 1E-04 | 0,113146 | 0,024392 | 0,210669 | 0,651693 |
| STE-U 9846 | 5 | 5 | 0,000728 | 0,044762 | 0,014254 | 0,179235 | 0,761021 |
| STE-U 9847 | 5 | 5 | 1E-04 | 0,028524 | 0,043483 | 0,273451 | 0,654442 |
| STE-U 9848 | 5 | 5 | 0,022445 | 1E-04 | 1E-04 | 0,152795 | 0,824561 |
| STE-U 9849 | 5 | 5 | 1E-04 | 0,036068 | 0,032199 | 0,221595 | 0,710038 |
| STE-U 9850 | 5 | 5 | 1E-04 | 1E-04 | 0,035282 | 0,218643 | 0,745875 |
| STE-U 9851 | 5 | 5 | 0,039237 | 0,023443 | 0,018467 | 0,218044 | 0,70081 |
| STE-U 9852 | 5 | 5 | 0,015149 | 0,020432 | 0,038481 | 0,281861 | 0,644077 |
| STE-U 9854 | 5 | 5 | 0,00706 | 1E-04 | 0,055122 | 0,117185 | 0,820533 |
| STE-U 9855 | 5 | 5 | 0,044019 | 1E-04 | 0,028519 | 0,274241 | 0,653121 |
| STE-U 9856 | 5 | 5 | 0,013254 | 0,002457 | 0,023523 | 0,25351 | 0,707255 |
| STE-U 9857 | 5 | 5 | 0,027548 | 1E-04 | 0,040655 | 0,430167 | 0,50153 |
| STE-U 9858 | 5 | 5 | 0,02142 | 0,047057 | 0,016646 | 0,29753 | 0,617347 |
| STE-U 9859 | 5 | 5 | 1E-04 | 0,080197 | 1E-04 | 0,262943 | 0,65666 |
| STE-U 9860 | 5 | 5 | 0,006067 | 1E-04 | 0,01907 | 0,185461 | 0,789302 |
| STE-U 9861 | 5 | 5 | 0,034336 | 0,040537 | 0,020944 | 0,19049 | 0,713692 |
| STE-U 9862 | 5 | 5 | 0,019254 | 0,014895 | 0,043027 | 0,270942 | 0,651882 |
| STE-U 9863 | 5 | 5 | 0,001001 | 0,081695 | 0,032974 | 0,186407 | 0,697923 |
| STE-U 9864 | 5 | 5 | 0,034988 | 1E-04 | 0,038729 | 0,238568 | 0,687615 |
| STE-U 9865 | 5 | 5 | 1E-04 | 1E-04 | 0,014066 | 0,106464 | 0,87927 |
| STE-U 9866 | 5 | 5 | 0,013925 | 0,082281 | 0,014969 | 0,274087 | 0,614739 |
| STE-U 9867 | 5 | 5 | 0,01945 | 0,025787 | 0,02589 | 0,274516 | 0,654357 |
| STE-U 9868 | 1 | 4 | 1E-04 | 0,025233 | 1E-04 | 0,942365 | 0,032202 |
| STE-U 9869 | 1 | 4 | 0,015262 | 0,03034 | 1E-04 | 0,878476 | 0,075821 |
| STE-U 9870 | 1 | 4 | 0,005338 | 0,032959 | 0,021148 | 0,879021 | 0,061534 |
| STE-U 9871 | 1 | 4 | 0,013643 | 0,042334 | 0,026789 | 0,814768 | 0,102466 |
| STE-U 9872 | 1 | 4 | 1E-04 | 0,065945 | 0,025616 | 0,89059 | 0,017748 |
| STE-U 9873 | 1 | 4 | 1E-04 | 0,054192 | 1E-04 | 0,875743 | 0,069865 |
| STE-U 9874 | 1 | 4 | 0,011108 | 0,01852 | 1E-04 | 0,942277 | 0,027995 |
| STE-U 9875 | 1 | 4 | 0,017588 | 0,067661 | 0,030713 | 0,781377 | 0,102661 |
| STE-U 9876 | 1 | 4 | 0,010539 | 0,02139 | 0,017897 | 0,890457 | 0,059718 |
| STE-U 9880 | 1 | 4 | 0,000783 | 0,099654 | 1E-04 | 0,899363 | 1E-04 |
| STE-U 9881 | 1 | 4 | 0,026392 | 0,020053 | 0,03225 | 0,877525 | 0,043782 |
| STE-U 9882 | 1 | 4 | 0,036076 | 1E-04 | 0,002625 | 0,86579 | 0,095409 |
| STE-U 9883 | 1 | 4 | 1E-04 | 0,02053 | 0,00682 | 0,866176 | 0,106374 |
| STE-U 9884 | 1 | 4 | 0,014768 | 0,035293 | 0,000917 | 0,948923 | 1E-04 |
| STE-U 9885 | 5 | 5 | 0,029133 | 0,113291 | 0,03971 | 0,198973 | 0,618894 |
| STE-U 9886 | 5 | 5 | 0,022645 | 1E-04 | 1E-04 | 0,331586 | 0,645569 |
| STE-U 9887 | 5 | 5 | 0,000145 | 1E-04 | 0,0351 | 0,33778 | 0,626876 |
| STE-U 9888 | 5 | 5 | 0,001933 | 0,062052 | 1E-04 | 0,228635 | 0,707279 |
| STE-U 9889 | 5 | 5 | 0,017075 | 0,109916 | 0,033628 | 0,215776 | 0,623605 |
| STE-U 9890 | 5 | 5 | 0,035857 | 0,050345 | 0,008619 | 0,343296 | 0,561883 |
| STE-U 9891 | 5 | 5 | 1E-04 | 0,058389 | 0,013062 | 0,136374 | 0,792074 |
| STE-U 9892 | 5 | 5 | 0,020358 | 0,056697 | 0,004692 | 0,307248 | 0,611006 |
| STE-U 9893 | 5 | 5 | 0,023001 | 0,114184 | 0,014969 | 0,292796 | 0,55505 |
| STE-U 9894 | 5 | 5 | 0,01188 | 0,000988 | 0,015284 | 0,240441 | 0,731407 |
| STE-U 9896 | 5 | 5 | 0,024661 | 1E-04 | 0,028728 | 0,175993 | 0,770518 |
| STE-U 9897 | 1 | 4 | 0,03608 | 0,003228 | 1E-04 | 0,854593 | 0,105999 |
| STE-U 9898 | 1 | 4 | 0,000964 | 0,005116 | 0,027471 | 0,91461 | 0,051838 |
| STE-U 9899 | 1 | 4 | 0,02459 | 0,00029 | 0,066212 | 0,850776 | 0,058132 |
| STE-U 9900 | 1 | 4 | 1E-04 | 0,065715 | 0,001277 | 0,814391 | 0,118516 |
| STE-U 9901 | 1 | 4 | 0,003113 | 0,041511 | 1E-04 | 0,818793 | 0,136484 |
| STE-U 9902 | 1 | 4 | 1E-04 | 0,006269 | 1E-04 | 0,954616 | 0,038915 |
| STE-U 9903 | 1 | 4 | 0,013418 | 0,024108 | 1E-04 | 0,93624 | 0,026135 |
| STE-U 9904 | 1 | 4 | 1E-04 | 0,05256 | 0,002737 | 0,908219 | 0,036383 |
| STE-U 9905 | 1 | 4 | 1E-04 | 0,077747 | 1E-04 | 0,585712 | 0,336341 |
| STE-U 9906 | 1 | 4 | 0,006304 | 0,057587 | 1E-04 | 0,935909 | 1E-04 |
| STE-U 9907 | 1 | 4 | 0,005033 | 0,045697 | 1E-04 | 0,94907 | 1E-04 |
| STE-U 9908 | 1 | 4 | 0,013399 | 1E-04 | 1E-04 | 0,986301 | 1E-04 |
| STE-U 9910 | 1 | 4 | 1E-04 | 0,038867 | 0,085034 | 0,845072 | 0,030927 |
| STE-U 9912 | 1 | 4 | 1E-04 | 0,064004 | 0,000896 | 0,934901 | 1E-04 |
| STE-U 9914 | 1 | 4 | 0,014388 | 0,000874 | 0,005732 | 0,881862 | 0,097145 |
| STE-U 9915 | 1 | 4 | 0,022272 | 1E-04 | 1E-04 | 0,866019 | 0,111509 |
| STE-U 9916 | 1 | 4 | 0,00661 | 0,066879 | 0,014745 | 0,911666 | 1E-04 |
| STE-U 9918 | 1 | 4 | 0,039356 | 1E-04 | 0,015712 | 0,825696 | 0,119136 |
| STE-U 9919 | 1 | 4 | 0,003568 | 1E-04 | 1E-04 | 0,899731 | 0,096501 |
| STE-U 9920 | 1 | 4 | 0,020731 | 1E-04 | 0,070866 | 0,849808 | 0,058495 |
| STE-U 9921 | 1 | 4 | 0,018864 | 0,032378 | 0,068241 | 0,880417 | 1E-04 |
| STE-U 9922 | 1 | 4 | 0,016743 | 0,031619 | 1E-04 | 0,898118 | 0,05342 |
| STE-U 9923 | 1 | 4 | 0,016962 | 0,127493 | 0,006881 | 0,681462 | 0,167201 |
| STE-U 9925 | 1 | 4 | 0,007975 | 1E-04 | 0,047439 | 0,771876 | 0,172611 |
| STE-U 9927 | 1 | 4 | 0,025948 | 0,016027 | 1E-04 | 0,896388 | 0,061537 |
| STE-U 9928 | 1 | 4 | 0,013667 | 0,031228 | 0,057968 | 0,769525 | 0,127612 |
| STE-U 9929 | 1 | 4 | 0,015987 | 1E-04 | 0,024175 | 0,761965 | 0,197773 |
| STE-U 9930 | 1 | 4 | 0,013104 | 0,049738 | 0,030439 | 0,673821 | 0,232898 |
| IBCN094 | 2 | 2 | 0,013655 | 0,923253 | 1E-04 | 0,062892 | 1E-04 |
| IBCN095 | 2 | 2 | 0,027611 | 0,917315 | 0,009227 | 0,045747 | 1E-04 |
| IBCN096 | 2 | 2 | 0,259445 | 0,689284 | 1E-04 | 0,036288 | 0,014883 |
| IBCN097 | 2 | 2 | 0,353295 | 0,597851 | 0,010869 | 0,03147 | 0,006516 |
| IBCN098 | 2 | 2 | 0,251044 | 0,673183 | 0,002729 | 0,047287 | 0,025757 |
| IBCN099 | 2 | 2 | 0,267658 | 0,645093 | 1E-04 | 0,056374 | 0,030774 |
| IBCN100 | 2 | 2 | 0,237729 | 0,694273 | 1E-04 | 0,029341 | 0,038557 |
| IBCN101^l^ | 3 | 1 | 0,9996 | 1E-04 | 1E-04 | 1E-04 | 1E-04 |
| IBCN102^l^ | 3 | 1 | 0,9996 | 1E-04 | 1E-04 | 1E-04 | 1E-04 |
| IBCN103^l^ | 3 | 1 | 0,9996 | 1E-04 | 1E-04 | 1E-04 | 1E-04 |
| IBCN104^l^ | 3 | 1 | 0,9996 | 1E-04 | 1E-04 | 1E-04 | 1E-04 |
| IBCN105 | 3 | 1 | 0,998873 | 0,000827 | 1E-04 | 1E-04 | 1E-04 |
| IBCN106 | 3 | 1 | 0,970911 | 1E-04 | 0,001094 | 0,026044 | 0,001852 |
| IBCN107^l^ | 3 | 1 | 0,9996 | 1E-04 | 1E-04 | 1E-04 | 1E-04 |
| IBCN108^l^ | 3 | 1 | 0,9996 | 1E-04 | 1E-04 | 1E-04 | 1E-04 |
| IBCN109^l^ | 3 | 1 | 0,9996 | 1E-04 | 1E-04 | 1E-04 | 1E-04 |
| IBCN110 | 3 | 1 | 0,891516 | 1E-04 | 0,101633 | 1E-04 | 0,006651 |
| IBCN111^l^ | 3 | 1 | 0,9996 | 1E-04 | 1E-04 | 1E-04 | 1E-04 |
| IBCN112 | 3 | 1 | 0,9996 | 1E-04 | 1E-04 | 1E-04 | 1E-04 |
| IBCN113 | 3 | 1 | 0,884091 | 1E-04 | 0,104678 | 1E-04 | 0,011031 |
| IBCN114^l^ | 3 | 1 | 0,9996 | 1E-04 | 1E-04 | 1E-04 | 1E-04 |
| IBCN115^l^ | 3 | 1 | 0,9996 | 1E-04 | 1E-04 | 1E-04 | 1E-04 |
| IBCN116^l^ | 3 | 1 | 0,9996 | 1E-04 | 1E-04 | 1E-04 | 1E-04 |
| IBCN118^l^ | 3 | 1 | 0,9996 | 1E-04 | 1E-04 | 1E-04 | 1E-04 |
| IBCN119 | 2 | 2 | 0,305979 | 0,448181 | 0,11032 | 0,13542 | 1E-04 |
| IBCN120 | 2 | 1 | 0,507285 | 0,292559 | 0,079224 | 0,086293 | 0,034639 |
| IBCN125 | 2 | 2 | 1E-04 | 0,827226 | 0,038692 | 0,011948 | 0,122034 |
| IBCN127 | 2 | 2 | 0,00442 | 0,904377 | 1E-04 | 1E-04 | 0,091003 |
| IBCN128 | 2 | 2 | 0,019269 | 0,94737 | 0,029435 | 1E-04 | 0,003825 |
| IBCN129 | 2 | 2 | 0,009267 | 0,858746 | 1E-04 | 0,054771 | 0,077115 |
| IBCN130 | 2 | 2 | 0,022461 | 0,943475 | 1E-04 | 0,02721 | 0,006754 |
| IBCN131 | 2 | 2 | 0,036377 | 0,863313 | 0,000463 | 1E-04 | 0,099747 |
| IBCN133 | 2 | 2 | 0,011957 | 0,935711 | 1E-04 | 0,009036 | 0,043196 |
| IBCN135 | 2 | 2 | 0,018577 | 0,855879 | 0,015815 | 1E-04 | 0,109629 |
| IBCN136 | 2 | 2 | 1E-04 | 0,930898 | 0,051466 | 0,017435 | 1E-04 |
| IBCN137 | 2 | 2 | 1E-04 | 0,930527 | 0,019761 | 0,006918 | 0,042694 |
| IBCN138 | 2 | 2 | 0,008049 | 0,88047 | 0,050627 | 0,002702 | 0,058152 |
| IBCN139 | 2 | 2 | 0,010632 | 0,873883 | 0,034146 | 1E-04 | 0,081239 |
| IBCN140 | 2 | 2 | 0,003503 | 0,887897 | 0,034028 | 0,039982 | 0,03459 |
| IBCN141 | 2 | 2 | 0,013696 | 0,87625 | 0,024955 | 0,020839 | 0,06426 |
| IBCN142 | 2 | 2 | 0,001673 | 0,91532 | 0,017883 | 0,030907 | 0,034218 |
| IBCN143 | 4 | 3 | 0,007015 | 0,036248 | 0,901479 | 0,001595 | 0,053662 |
| IBCN144 | 4 | 3 | 1E-04 | 0,035576 | 0,839756 | 0,014021 | 0,110547 |
| IBCN145 | 4 | 3 | 0,009141 | 1E-04 | 0,972784 | 0,017874 | 1E-04 |
| IBCN146 | 2 | 3 | 0,287213 | 0,251937 | 0,370686 | 0,055473 | 0,034691 |
| IBCN147 | 2 | 3 | 0,198954 | 0,266152 | 0,437939 | 0,029839 | 0,067116 |
| IBCN148 | 4 | 3 | 0,016188 | 0,287209 | 0,519263 | 0,050099 | 0,127241 |
| IBCN149 | 4 | 3 | 0,045064 | 0,308164 | 0,505284 | 0,141389 | 1E-04 |
| IBCN150 | 4 | 3 | 0,006242 | 0,063719 | 0,898956 | 0,030984 | 1E-04 |
| IBCN151 | 4 | 3 | 0,01204 | 1E-04 | 0,987661 | 1E-04 | 1E-04 |
| IBCN152 | 4 | 3 | 0,00945 | 0,119853 | 0,840222 | 0,030375 | 1E-04 |
| IBCN153 | 4 | 3 | 0,001786 | 0,028969 | 0,952103 | 1E-04 | 0,017042 |
| IBCN154 | 4 | 3 | 0,003324 | 1E-04 | 0,996376 | 1E-04 | 1E-04 |
| IBCN155 | 4 | 3 | 0,002676 | 1E-04 | 0,994868 | 0,00217 | 0,000186 |
| IBCN156 | 4 | 3 | 1E-04 | 1E-04 | 0,994407 | 1E-04 | 0,005293 |
| IBCN157 | 4 | 3 | 0,009585 | 0,026337 | 0,963878 | 1E-04 | 1E-04 |
| IBCN158 | 4 | 3 | 0,025725 | 0,278516 | 0,566056 | 0,053385 | 0,076319 |
| IBCN159 | 4 | 3 | 0,006176 | 0,062496 | 0,920946 | 0,010283 | 1E-04 |
| IBCN160 | 2 | 3 | 0,289481 | 0,239759 | 0,377302 | 0,093358 | 1E-04 |
| IBCN161 | 4 | 3 | 1E-04 | 1E-04 | 0,9996 | 1E-04 | 1E-04 |
| IBCN162^m^ | 4 | 3 | 1E-04 | 1E-04 | 0,987874 | 0,011827 | 1E-04 |
| IBCN163^m^ | 4 |  | 1E-04 | 1E-04 | 0,987874 | 0,011827 | 1E-04 |
| IBCN164 | 4 | 3 | 0,004372 | 0,017212 | 0,964766 | 1E-04 | 0,013551 |
| IBCN165 | 4 | 3 | 1E-04 | 0,031797 | 0,925546 | 0,012524 | 0,030033 |
| IBCN166 | 4 | 3 | 0,002485 | 0,047704 | 0,914136 | 1E-04 | 0,035575 |
| IBCN167 | 4 | 3 | 0,009724 | 0,049301 | 0,866883 | 0,006097 | 0,067995 |
| IBCN168 | 4 | 3 | 0,031827 | 1E-04 | 0,906594 | 0,010492 | 0,050987 |
| IBCN169^n^ | 4 | 3 | 1E-04 | 0,012982 | 0,949885 | 1E-04 | 0,036933 |
| IBCN170^n^ | 4 |  | 1E-04 | 0,012982 | 0,949885 | 1E-04 | 0,036933 |
| IBCN172 | 2 | 2 | 0,023409 | 0,951862 | 1E-04 | 0,0049 | 0,019729 |
| IBCN173 | 2 | 2 | 0,020705 | 0,862444 | 0,006663 | 0,014405 | 0,095784 |
| IBCN174 | 2 | 2 | 0,017937 | 0,895037 | 1E-04 | 1E-04 | 0,086826 |
| IBCN175 | 2 | 2 | 0,012597 | 0,89381 | 1E-04 | 1E-04 | 0,093393 |
| IBCN176 | 2 | 2 | 0,019959 | 0,840054 | 0,040917 | 1E-04 | 0,09897 |
| IBCN177 | 2 | 2 | 1E-04 | 0,917593 | 0,015553 | 0,03289 | 0,033864 |
| IBCN178 | 2 | 2 | 1E-04 | 0,881825 | 0,00522 | 1E-04 | 0,112756 |
| IBCN179 | 2 | 2 | 0,01641 | 0,841631 | 0,051479 | 0,045641 | 0,044839 |
| IBCN180 | 2 | 2 | 0,002152 | 0,894301 | 1E-04 | 0,006815 | 0,096631 |
| IBCN181 | 2 | 2 | 0,028116 | 0,879164 | 0,015628 | 0,07681 | 0,000282 |
| IBCN182 | 2 | 2 | 0,020673 | 0,827837 | 0,009118 | 0,049699 | 0,092672 |
| IBCN183 | 2 | 2 | 0,023958 | 0,78578 | 0,037508 | 0,010091 | 0,142663 |
| IBCN184 | 2 | 2 | 0,020116 | 0,917588 | 0,012814 | 0,022419 | 0,027064 |
| IBCN185 | 2 | 2 | 1E-04 | 0,963347 | 1E-04 | 0,036353 | 1E-04 |
| IBCN187^o^ | 2 | 2 | 1E-04 | 0,831239 | 0,033993 | 0,03354 | 0,101128 |
| IBCN188^o^ | 2 | 2 | 1E-04 | 0,831239 | 0,033993 | 0,03354 | 0,101128 |
| IBCN189^o^ | 2 | 2 | 1E-04 | 0,831239 | 0,033993 | 0,03354 | 0,101128 |
| IBCN190 | 2 | 2 | 1E-04 | 0,844734 | 0,009892 | 1E-04 | 0,145174 |
| IBCN191 | 2 | 2 | 0,003685 | 0,858491 | 1E-04 | 1E-04 | 0,137624 |
| IBCN193 | 2 | 2 | 1E-04 | 0,835994 | 0,047096 | 1E-04 | 0,116711 |
| IBCN194^l^ | 3 | 1 | 0,9996 | 1E-04 | 1E-04 | 1E-04 | 1E-04 |
| IBCN195^l^ | 3 | 1 | 0,9996 | 1E-04 | 1E-04 | 1E-04 | 1E-04 |
| IBCN196^p^ | 2 | 2 | 0,15384 | 0,721045 | 0,046995 | 0,073829 | 0,004291 |
| IBCN197^p^ | 2 | 2 | 0,15384 | 0,721045 | 0,046995 | 0,073829 | 0,004291 |
| IBCN198 | 2 | 2 | 0,097642 | 0,760447 | 0,064446 | 0,060492 | 0,016973 |
| IBCN199 | 2 | 2 | 0,169946 | 0,678744 | 0,088062 | 0,017532 | 0,045716 |
| IBCN200 | 3 | 1 | 0,89104 | 0,062789 | 1E-04 | 0,020272 | 0,025799 |
| IBCN201^l^ | 3 | 1 | 0,9996 | 1E-04 | 1E-04 | 1E-04 | 1E-04 |
| IBCN202^l^ | 3 | 1 | 0,9996 | 1E-04 | 1E-04 | 1E-04 | 1E-04 |
| IBCN203^l^ | 3 | 1 | 0,9996 | 1E-04 | 1E-04 | 1E-04 | 1E-04 |
| IBCN204^l^ | 3 | 1 | 0,9996 | 1E-04 | 1E-04 | 1E-04 | 1E-04 |
| IBCN205 | 3 | 1 | 0,734331 | 0,113805 | 0,11723 | 0,015431 | 0,019203 |
| IBCN206^l^ | 3 | 1 | 0,9996 | 1E-04 | 1E-04 | 1E-04 | 1E-04 |
| IBCN207^l^ | 3 | 1 | 0,9996 | 1E-04 | 1E-04 | 1E-04 | 1E-04 |
| IBCN208 | 3 | 1 | 0,933721 | 1E-04 | 0,061616 | 0,004463 | 1E-04 |
| IBCN209 | 3 | 1 | 0,906725 | 0,023978 | 0,022488 | 0,008008 | 0,0388 |
| IBCN210 | 3 | 1 | 0,95822 | 1E-04 | 0,037151 | 0,00443 | 1E-04 |
| IBCN211 | 2 | 1 | 0,464452 | 0,323053 | 0,102693 | 0,107194 | 0,002609 |
| IBCN212 | 3 | 1 | 0,77032 | 0,143562 | 0,083122 | 1E-04 | 0,002896 |
| IBCN213 | 3 | 1 | 0,961797 | 1E-04 | 0,037903 | 1E-04 | 1E-04 |
| IBCN214 | 3 | 1 | 0,862415 | 0,010169 | 0,078983 | 0,026952 | 0,021482 |
| IBCN215 | 3 | 1 | 0,908858 | 0,00269 | 0,061272 | 0,02708 | 1E-04 |
| IBCN216 | 3 | 1 | 0,798834 | 0,046184 | 0,114563 | 0,040319 | 1E-04 |
| IBCN217 | 3 | 1 | 0,934708 | 0,007946 | 0,057146 | 1E-04 | 1E-04 |
| IBCN218 | 3 | 1 | 0,99394 | 1E-04 | 1E-04 | 0,00576 | 1E-04 |
| IBCN224^r^ | 3 | 1 | 0,9996 | 1E-04 | 1E-04 | 1E-04 | 1E-04 |
| IBCN237^q^ | 3 | 1 | 0,937707 | 0,061668 | 1E-04 | 0,000425 | 1E-04 |
| IBCN238^q^ | 3 | 1 | 0,937707 | 0,061668 | 1E-04 | 0,000425 | 1E-04 |
| IBCN239^r^ | 3 | 1 | 0,9996 | 1E-04 | 1E-04 | 1E-04 | 1E-04 |
| IBCN240^r^ | 3 | 1 | 0,9996 | 1E-04 | 1E-04 | 1E-04 | 1E-04 |
| IBCN241 | 1 | 4 | 1E-04 | 0,077473 | 0,004586 | 0,863922 | 0,053919 |
| IBCN242^l^ | 1 | 4 | 0,021336 | 1E-04 | 0,038826 | 0,77609 | 0,163648 |
| IBCN243^l^ | 1 | 4 | 0,021336 | 1E-04 | 0,038826 | 0,77609 | 0,163648 |
| IBCN244 | 1 | 4 | 0,006041 | 0,050125 | 1E-04 | 0,943633 | 1E-04 |
| IBCN245 | 1 | 4 | 0,003127 | 1E-04 | 0,005769 | 0,957825 | 0,03318 |
| IBCN246 | 5 | 5 | 0,010312 | 0,010576 | 1E-04 | 0,194425 | 0,784587 |
| IBCN247 | 5 | 5 | 0,005403 | 0,073952 | 0,020947 | 0,272753 | 0,626945 |
| IBCN249 | 1 | 4 | 0,015934 | 1E-04 | 0,001408 | 0,925059 | 0,057499 |
| IBCN250 | 1 | 4 | 0,007262 | 0,108011 | 0,075021 | 0,809607 | 1E-04 |
| IBCN251^g^ | 1 | 4 | 0,005157 | 0,026448 | 0,002057 | 0,966238 | 1E-04 |
| IBCN252 | 5 | 5 | 0,033827 | 0,070172 | 0,025507 | 0,183912 | 0,686583 |
| IBCN253 | 1 | 4 | 1E-04 | 0,071858 | 1E-04 | 0,894641 | 0,033301 |
| IBCN254 | 5 | 5 | 0,010108 | 0,064438 | 0,004872 | 0,261976 | 0,658606 |
| IBCN255 | 5 | 5 | 0,005897 | 0,115377 | 0,034524 | 0,209564 | 0,634639 |
| IBCN256 | 5 | 5 | 0,011854 | 0,020889 | 0,083366 | 0,247839 | 0,636052 |
| IBCN257 | 5 | 5 | 0,019405 | 0,018981 | 0,001969 | 0,330815 | 0,62883 |
| IBCN258 | 5 | 5 | 0,009629 | 0,097527 | 0,004413 | 0,247743 | 0,640688 |
| IBCN259 | 5 | 5 | 0,014264 | 0,010962 | 0,002252 | 0,163222 | 0,809301 |
| IBCN260 | 5 | 5 | 0,012281 | 0,048018 | 0,022474 | 0,203899 | 0,713328 |
| IBCN261 | 5 | 5 | 0,02026 | 0,017088 | 0,04064 | 0,168565 | 0,753447 |
| IBCN262 | 1 | 4 | 1E-04 | 0,03071 | 0,00889 | 0,9602 | 1E-04 |
| IBCN263 | 1 | 4 | 0,008169 | 0,018787 | 0,031872 | 0,921181 | 0,019992 |
| IBCN264 | 1 | 4 | 0,001766 | 0,081961 | 1E-04 | 0,916073 | 1E-04 |
| IBCN265 | 1 | 4 | 0,004628 | 1E-04 | 1E-04 | 0,927127 | 0,068045 |
| IBCN266 | 1 | 4 | 0,011962 | 0,056036 | 0,01098 | 0,920922 | 1E-04 |
| IBCN267 | 1 | 4 | 0,026069 | 0,009488 | 0,019264 | 0,831854 | 0,113325 |
| IBCN268 | 1 | 4 | 0,004574 | 1E-04 | 0,028153 | 0,967072 | 1E-04 |
| IBCN269 | 5 | 5 | 0,003409 | 0,07521 | 1E-04 | 0,156622 | 0,764659 |
| IBCN270 | 5 | 5 | 0,024095 | 1E-04 | 0,021116 | 0,219336 | 0,735354 |
| IBCN271 | 5 | 5 | 0,017939 | 0,028396 | 0,007806 | 0,259448 | 0,686411 |
| IBCN272 | 5 | 5 | 0,009539 | 0,062793 | 1E-04 | 0,188832 | 0,738737 |
| IBCN273 | 5 | 5 | 0,026804 | 0,086153 | 0,011119 | 0,230297 | 0,645628 |
| IBCN274 | 5 | 5 | 1E-04 | 0,063267 | 0,008844 | 0,245657 | 0,682132 |
| IBCN275 | 1 | 4 | 1E-04 | 0,06183 | 0,013608 | 0,886385 | 0,038077 |
| IBCN276 | 2 | 2 | 0,031794 | 0,820543 | 0,011286 | 0,018045 | 0,118332 |
| IBCN277 | 2 | 2 | 0,008284 | 0,877722 | 0,032444 | 0,013096 | 0,068455 |
| IBCN278 | 2 | 2 | 0,0171 | 0,879959 | 0,00945 | 0,093392 | 1E-04 |
| IBCN279 | 2 | 2 | 1E-04 | 0,859146 | 0,022903 | 0,074542 | 0,043309 |
| IBCN280 | 2 | 2 | 0,011461 | 0,871808 | 0,036537 | 1E-04 | 0,080095 |
| IBCN281 | 2 | 2 | 0,021841 | 0,851476 | 0,013738 | 0,075579 | 0,037366 |
| IBCN282 | 2 | 2 | 0,005505 | 0,953407 | 0,01463 | 0,026357 | 1E-04 |
| IBCN283 | 2 | 2 | 0,029516 | 0,79996 | 0,005174 | 0,000932 | 0,164418 |
| IBCN284 | 2 | 2 | 0,011935 | 0,944043 | 1E-04 | 0,043822 | 1E-04 |
| IBCN285 | 2 | 2 | 0,008126 | 0,852546 | 0,043204 | 0,030309 | 0,065814 |
| IBCN286 | 2 | 2 | 0,001073 | 0,862986 | 0,038801 | 0,091002 | 0,006138 |
| IBCN288 | 2 | 2 | 0,008168 | 0,857476 | 0,014317 | 0,052048 | 0,067992 |
| IBCN289 | 2 | 2 | 0,001903 | 0,827656 | 0,025666 | 1E-04 | 0,144676 |
| IBCN290 | 2 | 2 | 0,020246 | 0,921967 | 0,017053 | 0,040635 | 1E-04 |
| IBCN291 | 2 | 2 | 0,026964 | 0,826128 | 0,062112 | 0,027061 | 0,057736 |
| IBCN292 | 2 | 2 | 0,027861 | 0,814039 | 0,011427 | 0,035749 | 0,110925 |
| IBCN293 | 2 | 2 | 0,002856 | 0,831678 | 0,042308 | 0,037065 | 0,086093 |
| IBCN294 | 2 | 2 | 0,006227 | 0,809995 | 0,038878 | 0,068443 | 0,076458 |
| IBCN295 | 2 | 2 | 0,00623 | 0,863498 | 0,044727 | 0,066813 | 0,018732 |
| IBCN297 | 2 | 2 | 1E-04 | 0,930706 | 0,02 | 0,045738 | 0,003457 |
| IBCN299 | 2 | 2 | 0,015279 | 0,884259 | 1E-04 | 0,04107 | 0,059292 |
| IBCN300 | 2 | 2 | 0,023742 | 0,876912 | 0,043316 | 0,032093 | 0,023938 |
| IBCN301 | 2 | 2 | 1E-04 | 0,8886 | 0,040654 | 0,070547 | 1E-04 |
| IBCN302 | 2 | 2 | 0,038736 | 0,826068 | 0,010101 | 0,066065 | 0,05903 |
| IBCN303 | 2 | 2 | 0,029115 | 0,838309 | 0,066715 | 0,044089 | 0,021772 |
| IBCN304 | 2 | 2 | 1E-04 | 0,920328 | 0,008624 | 0,031687 | 0,039261 |
| IBCN305 | 2 | 2 | 0,03693 | 0,863895 | 0,006232 | 0,065125 | 0,027818 |
| IBCN306 | 1 | 4 | 0,037923 | 0,079813 | 0,101726 | 0,60765 | 0,172889 |
| IBCN307 | 1 | 4 | 0,033617 | 0,118393 | 0,152257 | 0,695634 | 1E-04 |
| IBCN308 | 1 | 4 | 0,004403 | 0,11043 | 0,122496 | 0,703618 | 0,059053 |
| IBCN309 | 1 | 4 | 0,030609 | 0,071975 | 0,116704 | 0,738117 | 0,042595 |
| IBCN310 | 1 | 4 | 0,034914 | 0,073469 | 0,169763 | 0,637457 | 0,084397 |
| IBCN311 | 1 | 4 | 0,00875 | 0,173511 | 0,105448 | 0,709205 | 0,003086 |
| IBCN312 | 1 | 4 | 0,024108 | 0,08307 | 0,147567 | 0,623803 | 0,121451 |
| IBCN313 | 1 | 4 | 0,012209 | 0,140025 | 0,124221 | 0,658632 | 0,064914 |
| IBCN314 | 1 | 4 | 0,012521 | 0,216708 | 0,075739 | 0,694933 | 1E-04 |
| IBCN315 | 1 | 4 | 0,045603 | 0,228649 | 0,080411 | 0,645237 | 1E-04 |
| IBCN316 | 1 | 4 | 0,004448 | 0,221987 | 0,070494 | 0,702971 | 1E-04 |
| IBCN317 | 1 | 4 | 0,016164 | 0,089018 | 0,162449 | 0,702731 | 0,029639 |
| IBCN318 | 1 | 4 | 0,032853 | 0,138916 | 0,070004 | 0,732009 | 0,026218 |
| IBCN319 | 1 | 4 | 0,029205 | 0,066365 | 0,16838 | 0,633217 | 0,102833 |
| IBCN320 | 1 | 4 | 0,044583 | 0,102241 | 0,083322 | 0,658844 | 0,11101 |
| IBCN321 | 1 | 4 | 0,022443 | 0,030487 | 0,179862 | 0,643131 | 0,124077 |
| IBCN322 | 1 | 4 | 0,030568 | 0,212044 | 0,059464 | 0,644062 | 0,053862 |
| IBCN323 | 1 | 4 | 0,028931 | 0,099713 | 0,114601 | 0,61291 | 0,143845 |
| IBCN324 | 1 | 4 | 0,006961 | 0,117406 | 0,116673 | 0,692106 | 0,066854 |
| IBCN325 | 1 | 4 | 0,028527 | 0,08246 | 0,127277 | 0,695815 | 0,065921 |
| IBCN326 | 1 | 4 | 0,007577 | 0,194103 | 0,10476 | 0,69346 | 1E-04 |
| IBCN327 | 1 | 4 | 0,020116 | 0,170905 | 0,115551 | 0,646415 | 0,047013 |
| IBCN328 | 1 | 4 | 0,01662 | 0,15574 | 0,156503 | 0,653961 | 0,017177 |
| IBCN329 | 1 | 4 | 0,0284 | 0,12958 | 0,116391 | 0,651298 | 0,074331 |
| IBCN330 | 1 | 4 | 0,054468 | 0,077792 | 0,132999 | 0,636821 | 0,09792 |

^a^Isolates with the same superscript letter collapsed into one multilocus lineage at a distance threshold of 0.01
