# Supplementary material for: Whole genome sequencing reveals the genetic diversity and structure of Leptosphaeria maculans populations from the Western Cape province of South Africa
Source: BMC Genomics. 2025 Apr 3;26:334. doi: 10.1186/s12864-025-11413-3 (PMC11966903; doi:10.1186/s12864-025-11413-3)
Supplement: Supplementary file 7 — Supplementary Material 7 [file 12864_2025_11413_MOESM7_ESM.docx]

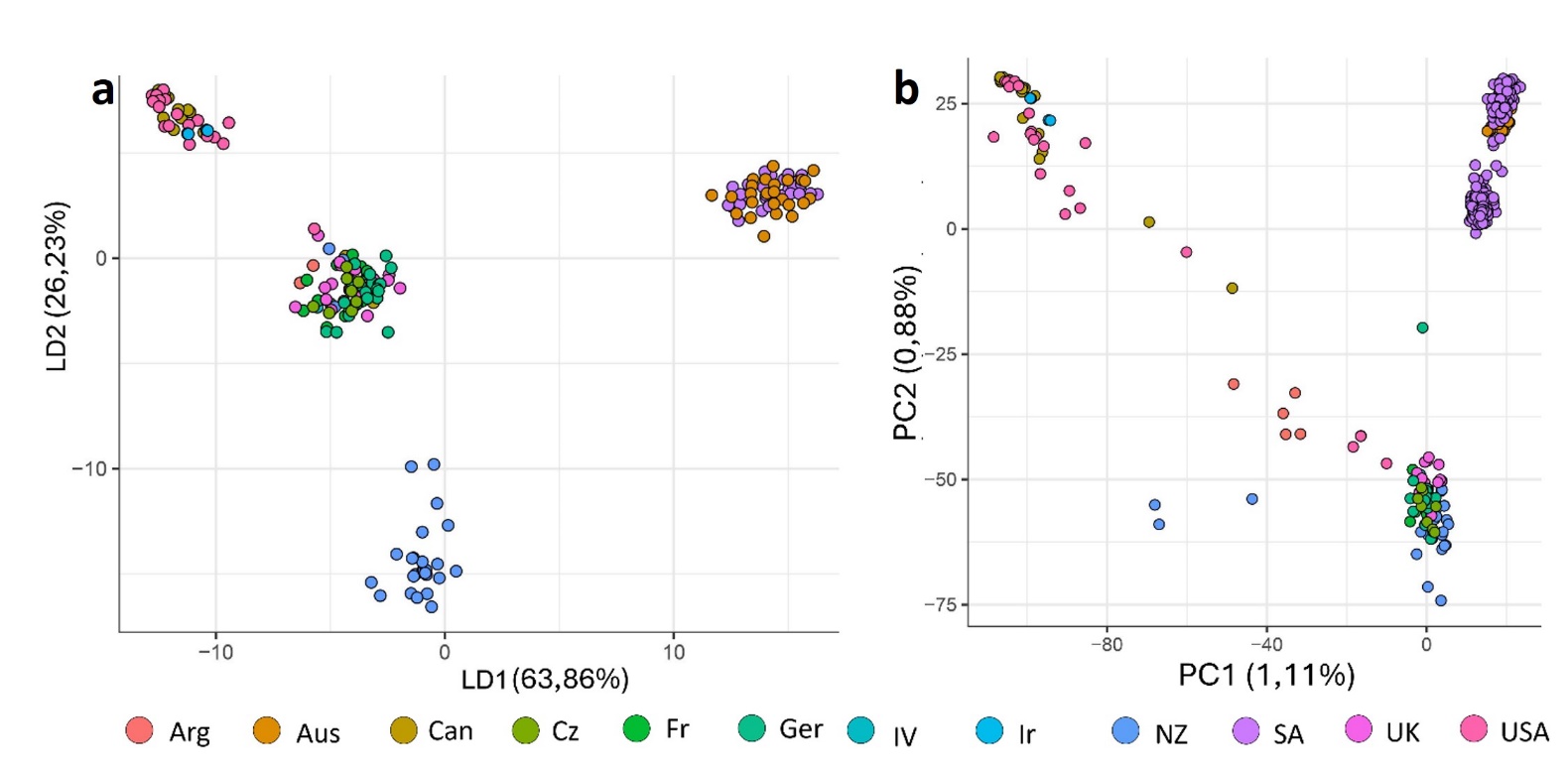


**Fig. S1** Visualisations of population analyses conducted on an international *Leptosphaeria maculans* isolate collection using informative single nucleotide polymorphisms (SNPs). Each point represents an individual isolate and colours denote their location of origin. Arg = Argentina; Aus = Australia; Can = Canada; Cz = Czech Republic; Fr = France; Ger = Germany; IV = In vitro; Ir = Iran; Nz = New Zealand; SA = South Africa; UK = United Kingdom; USA = United States of America **a)** Scatterplot generated from the discriminant analysis of principal components (DAPC) on 205 isolates using the first 20 principal components (PCs) and three discriminant functions (LD). **b)** Scatterplot generated from the principal component analysis (PCA) on the 205 isolates used in a) and an additional 230 South African isolates.
